# Synthesis and Anticancer Properties of New 3-Methylidene-1-sulfonyl-2,3-dihydroquinolin-4(1*H*)-ones

**DOI:** 10.3390/molecules27113597

**Published:** 2022-06-03

**Authors:** Agata Jaskulska, Katarzyna Gach-Janczak, Joanna Drogosz-Stachowicz, Tomasz Janecki, Anna Ewa Janecka

**Affiliations:** 1 Institute of Organic Chemistry, Faculty of Chemistry, Lodz University of Technology, Żeromskiego 116, 90-924 Lodz, Poland; agata.jaskulska@dokt.p.lodz.pl; 2 Department of Biomolecular Chemistry, Faculty of Medicine, Medicinal University of Lodz, Mazowiecka 6/8, 92-215 Lodz, Poland; katarzyna.gach@umed.lodz.pl (K.G.-J.); joanna.drogosz@stud.umed.lodz.pl (J.D.-S.)

**Keywords:** Horner–Wadsworth–Emmons olefination, structure–activity relationship, cytotoxicity, apoptosis

## Abstract

Quinolinones have been known for a long time as broad-spectrum synthetic antibiotics. More recently, the anticancer potential of this group of compounds has been investigated. Following this direction, we obtained a small library of 3-methylidene-1-sulfonyl-2,3-dihydroquinolin-4(1*H*)-ones with various substituents at positions 1, 2, 6, and 7 of the quinolinone ring system. The cytotoxic activity of the synthesized analogs was tested in the MTT assay on two cancer cell lines in order to determine the structure–activity relationship. All compounds produced high cytotoxic effects in MCF-7, and even higher in HL-60 cells. 2-Ethyl-3-methylidene-1-phenylsulfonyl-2,3-dihydroquinolin-4(1*H*)-one, which was over 5-fold more cytotoxic for HL-60 than for normal HUVEC cells, was selected for further tests. This analog was shown to inhibit proliferation and induce DNA damage and apoptosis in HL-60 cells.

## 1. Introduction

Natural products, defined as chemical substances produced by living organisms, were generated for various purposes, the main of which was to develop and maintain different forms of life. According to the published data, 73% of currently available medications are either natural compounds isolated from plants, bacteria, or marine invertebrates or their chemically modified analogs [1]. The remaining 27% are totally synthetic drugs found by random screening.

**Figure 1:**
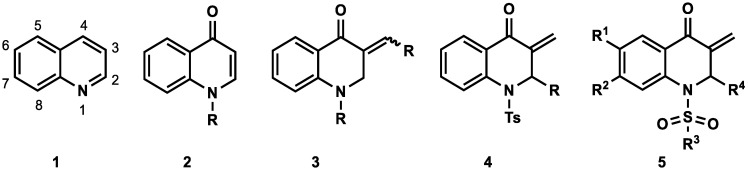
Chemical structure of quinoline, 4-quinolinones, and some diversely substituted analogs with quinolone skeleton.

Quinoline **1** is a compound found in coal tar. Numerous derivatives of quinoline, modified in one or both rings, were isolated from plants. A distinct group of natural compounds derived from quinoline are substituted quinolin-4-ones, also referred to as quinol-4-ones **2** (Figure 1) [1,2,3]. These compounds exhibit interesting biological activities and were used as intermediates in the synthesis of antibacterial, antifungal, and antimalarial agents [3,4,5,6]. More recently, the anticancer properties of synthetic, diversely substituted quinolin-4-ones have been extensively studied [7,8,9,10].

It is well known that the presence, position, and character of substituents play an important role in defining the biological activity of quinolinone molecules [11]. Substitution on nitrogen atom at position 1 is essential for overall potency, as well as the presence of a carbonyl group at position 4. The nature of substituents at positions 5–8 can affect the configuration of quinolinone molecules and influence anticancer activity. The substituent at position 3 should be co-planar with quinolinone moiety and the position 2 substituent should not disturb this co-planarity. One group of quinolinones, in which the condition of co-planarity is evidently fulfilled, are 3-alkylidenequinolin-4-ones **3**. Furthermore, an *exo*-cyclic alkylidene moiety conjugated with a carbonyl group present in analogs **3** is a pharmacophoric unit known to be responsible for the cytotoxic properties of many natural products, including sesquiterpene lactones [12,13,14]. The cytotoxicity of compounds with an α,β-unsaturated carbonyl group is the result of their ability to alkylate cellular thiols in enzymes, other functional proteins, and in free intracellular glutathione, which disrupts some major processes in the cell, leading to inhibition of proliferation and the induction of apoptosis [15].

In our earlier paper, we introduced an *exo*-cyclic methylidene fragment into a quinolinone moiety, preparing 3-methylidene-2,3-dihydroquinolin-4-ones **4** (Figure 1) which were strongly cytotoxic against several cancer cell lines [16].

Encouraged by these results, herein, we describe the synthesis and cytotoxic activity of a series of 3-methylidene-1-sulfonyl-2,3-dihydroquinolin-4(1*H*)-ones **5** (Figure 1) which were obtained in order to establish how various substituents in positions 1, 2, 6, and 7 of the quinolinone molecule can influence the in vitro cytotoxicity against promyelocytic leukemia HL-60 and breast cancer adenocarcinoma MCF-7. Normal human umbilical vein endothelial cells HUVEC were used for comparison. The most promising analog in terms of cytotoxicity and selectivity, 2-ethyl-3-methylidene-1-(phenylsulfonyl)-2,3-dihydroquinolin-4(1*H*)-one (**5a**), was evaluated for its ability to inhibit cell proliferation, induce DNA damage and apoptosis, and influence the mRNA level of an ABCB1 transporter that may be responsible for multidrug resistance in cancer cells.

## 2. Results and Discussion

### 2.1. Chemistry

**Scheme 1:**
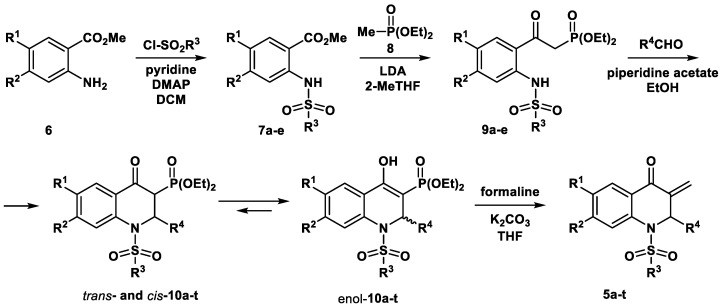
Synthesis of 3-methylidene-1-sulfonyl-2,3-dihydroquinolin-4(1*H*)-ones **5a-t**.

The target compounds were synthesized by applying a modified methodology originally reported for the synthesis of 3-methylidene-1-tosyl-2,3-dihydroquinolin-4(1*H*)-ones [16]. Starting materials, methyl 2-(arylsulfonylamino)benzoates **7a-e**, were synthesized by *N*-sulfonylation of the corresponding methyl 2-aminobenzoates **6** with arylsulfonyl chlorides. After crystallization with methanol, we obtained pure products **7a-e** in good-to-moderate yields (58–85%) (Scheme 1). Next, we performed acylation of diethyl methylphosphonate (**8**) with obtained benzoates **7a-e** in the presence of three equivalents of LDA and expected diethyl 2-oxo-2-[(2-arylsulfonylamino)phenyl]ethylphosphonate **9a-e** were formed in good yields (62–87%). Condensation of **9a-e** with selected alkyl and aryl aldehydes followed by the spontaneous intramolecular aza-Michael addition delivered 2-substituted 3-diethoxyphosphoryl-1-sulfonyl-2,3-dihydroquinolin-4(1*H*)-ones **10a-t**. The piperidine acetate was used as a catalyst and the reaction mixtures were stirred for 24–48 h at room temperature. Progress of the reaction was monitored by ^31^P-NMR. The crude products were purified by column chromatography. ^1^H, ^13^C, and ^31^P NMR spectra showed that obtained compounds were formed as mixtures of *trans*- and *cis*-2,3-dihydroquinolin-4(1*H*)-ones *trans*- and *cis*-**10a-t** and 3-diethoxyphosphoryl-1-sulfonyl-2,3-dihydroquinolin-4(1*H*)-ols **10a-t,** enol-**10a-t**, with the enol form strongly predominating (70–99%). Ketones **10** were formed as single *trans*-isomers or as mixtures of *cis-* and *trans*-isomers. Careful analysis of the ^1^H NMR spectra of the obtained compounds revealed characteristic singlets or doublets with chemical shifts in the range of 10.57–10.90 ppm and ^4^*J*_H-_*p* = 0.9–1.1 Hz, which were attributed to the protons of the hydroxyl group of the enol form. In case of ketones, configurational assignments were made on the bases of characteristic ^3^*J*_H2-H3_ coupling constants which were in the range of 1.1–1.5 Hz for *trans* isomers and 4.5–5.8 Hz for *cis* isomers. Thorough NMR analysis of 2-substituted 3-diethoxyphosphoryl-2,3-dihydroquinolinones was presented in our previous paper [16], and the results obtained in this work are with full agreement with that analysis. Table 1 shows *trans*- and *cis*-**10a-t** and enol-**10a-t** ratios, as well as yields of the synthesized products.

In the final step, mixtures of *trans*- and *cis*-2,3-dihydroquinolin-4(1*H*)-ones *trans*- and *cis*-**10a-t** and 2,3-dihydroquinolin-4(1*H*)-ols enol-**10a-t** were successfully used as Horner–Wadsworth–Emmons reagents in the olefination of formaldehyde. We performed the reaction using the standard procedure, with 30% formalin as a source of formaldehyde, in the presence of K_2_CO_3_ as a base. Progress of the reaction was monitored by TLC chromatography. We noticed that the time needed for completion of the reaction was significantly longer for **10** with alkyl substituents in position 2 (16 h) in comparison to aryl ones (3 h). After standard work-up and column chromatography of the crude products, the pure analogs **5a-t** were obtained in yields given in Table 1.

### 2.2. Biology

#### 2.2.1. In Vitro Cytotoxic Activity

The cytotoxicity of a series of 3-methylidene-1-sulfonyl-2,3-dihydroquinolin-4(1*H*)-ones **5a-t** was tested against HL-60 and MCF-7 cell lines using the MTT assay. Carboplatin was used as a reference compound. The obtained results are summarized in Table 2. After 48 h incubation, all new analogs showed cytotoxic activity in low µM range against both cancer cell lines. The tested compounds were more cytotoxic for HL-60 than for MCF-7 cells. Analysis of the structure activity relationship revealed that quinolin-4(1*H*)-ones with alkyl substituent in position 2 were more potent than those bearing an aryl substituent in that position. In general, with one exception (**5i**), the highest activity was observed for analogs containing *i*-propyl substituent in position 2. In HL-60 cells, the most active analogs **5b**, **5f**, and **5r** had IC_50_ values below 0.3 µM. Compounds **5m-p**, with chlorine at position 6, were less but still very cytotoxic. The substituent on the nitrogen atom did not significantly affect the activity of the tested compounds.

**Figure 2:**
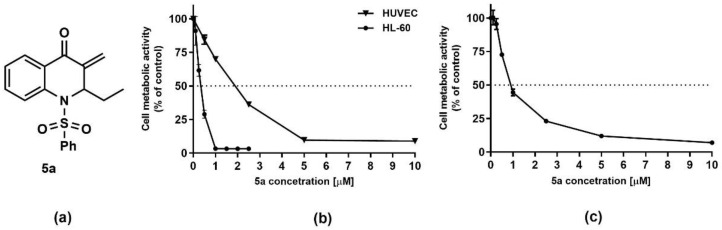
Structure of the selected compound **5a** (**a**). Metabolic activity of (**b**) HL-60 and HUVEC cells treated with various concentrations of **5a** for 48 h; (**c**) HL-60 cells treated with various concentration of **5a** for 24 h, measured by MTT assay.

In order to evaluate the influence of the new analogs on normal, non-cancerous cells, the selected compounds were tested on the HUVEC cell line. In most cases, the differences in IC_50_ values between normal and cancer cells were not very significant. Analog **5a** (Figure 2a) showed the highest selectivity and was over 5-fold more cytotoxic for HL-60 than for HUVEC cells (Figure 2b). For comparison, the HUVEC/HL-60 IC_50_ ratio for carboplatin was 1.41.

Compound **5a** was chosen for a more detailed evaluation of its anticancer potential in the HL-60 cell line. The MTT assay was performed for **5a** after 24 h incubation and the IC_50_ value was 0.91 ± 0.03 µM (Figure 2c). Based on this data, three concentrations (0.9, 1.35 and 1.8 µM) were chosen for further experiments.

#### 2.2.2. Inhibition of Cell Proliferation, Generation of DNA Damage, and Induction of Apoptosis by **5a**

**Figure 3:**
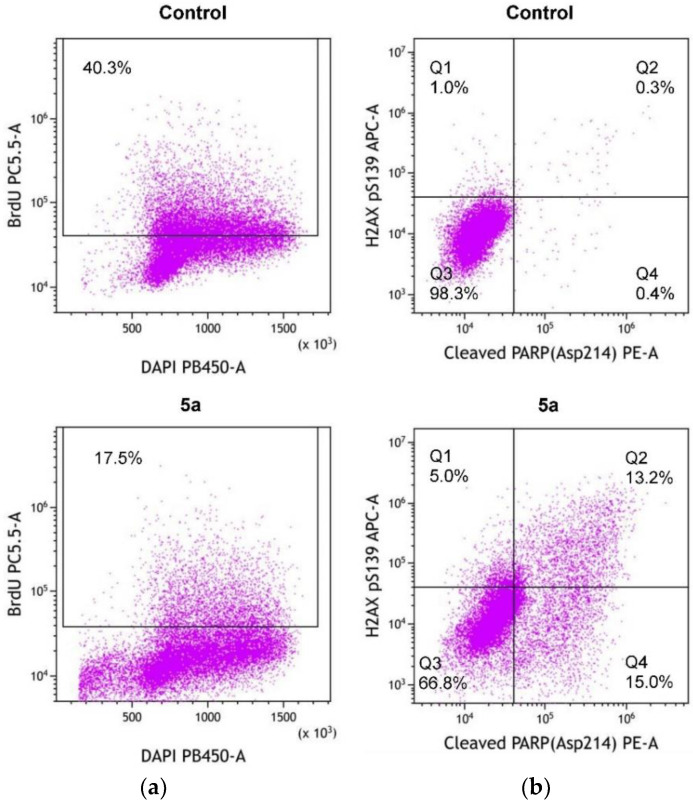
Representative scattered blots of multiparameter flow cytometry analysis of cell proliferation, DNA damage and apoptosis in HL-60 cells treated with **5a** (1.8µM) for 24 h. Panel (**a**): DAPI vs. BrdU PerCP-Cy.5.5 staining profile. BrdU positive cells are in the inside frame. Panel (**b**): cleaved PARP (Asp214) PE vs. H2AX (pS139) Alexa profile. Squares Q1 + Q2 represent H2AX (pS139) Alexa positive cells, squares Q2 + Q4 represent cleaved PARP (Asp214) PE positive cells, whereas square Q3 represents H2AX (pS139) Alexa and cleaved PARP (Asp214) PE negative cells.

To evaluate the anticancer potential of **5a**, the ability of this compound to inhibit cell proliferation, induce apoptotic cell death, and generate DNA damage was examined by flow cytometry using the ‘Apoptosis, DNA Damage, and Cell Proliferation Kit’ (BD Bioscience). After 24 h treatment with **5a** in increasing concentrations, HL-60 cells were exposed to bromodeoxyuridine (BrdU) for additional 8 h. Then, the cells were simultaneously stained with fluorochrome-labeled anti-BrdU, anti-cleaved PARP (Asp214) and anti-H2AX (pS139). The representative results of multiparameter flow cytometry analysis are shown in Figure 3.

**Figure 4:**
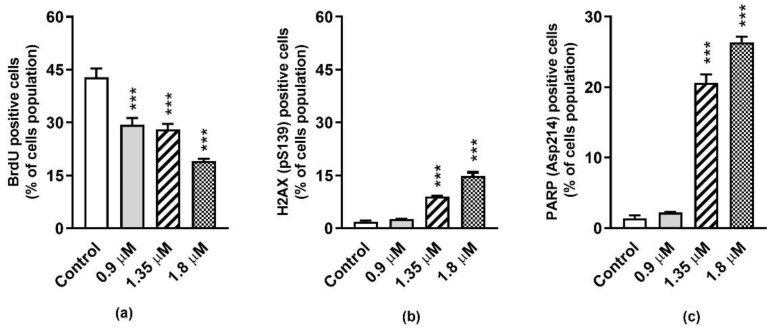
Quantitative analysis of inhibition of cell proliferation (BrdU test); (**a**) induction of apoptosis (PARP test) (**b**) and induction of DNA damage (H2AX test) (**c**) by **5a** (1.8 µM) in HL-60 cells. Data are presented as mean ± SEM of three independent experiments. Statistical significance was assessed using one-way ANOVA and a post-hoc multiple comparison Student–Newman–Keuls test. *** *p* < 0.001 vs. control.

BrdU is an analog of thymidine, and its incorporation into DNA was used as an index of cell proliferation. **5a** dose-dependently inhibited cell proliferation. At 1.8 µM concentration, this analog caused a 2.2-fold decrease in BrdU incorporation (Figure 4a).

To check whether the cytotoxic effect of **5a** was associated with the induction of apoptosis, the pro-apoptotic activity of this compound was investigated. Apoptosis regulation is triggered by the activation of caspases [17]. Caspase-3, an effector caspase, cleaves a number of vital proteins, leading to cell death. The activity of caspase-3 was assessed by flow cytometry using fluorochrome-labeled antibodies recognizing 89 kDa-cleaved PARP fragments released from PARP by caspase-3 in the executive stage of apoptosis. Incubation of HL-60 cells with **10a** (at 1.35 and 1.8 µM) for 24 h led to a significant increase in the number of apoptotic cells, whose population raised from 1.4% for control to 20.6% and 26.4%, respectively (Figure 4b).

It is well documented that apoptosis induced by many anticancer drugs may be a consequence of DNA damage [18]. Chemical genotoxins that target DNA can inhibit DNA replication, which leads to the collapse of replication forks and the formation of DNA double strand breaks (DSBs). The generation of DNA damage was evaluated using anti-H2AX (pS139) antibodies directed against the phosphorylated form of human H2AX protein at the pS139 residue that is considered to be a biomarker for DNA DSBs. The treatment of HL-60 cells with **5a** increased the levels of phosphorylated H2AX by 4.7- and 7.8-fold (to 1.35 µM and 1.9 µM, respectively), indicating the dose-dependent genotoxic effect of the tested compound (Figure 4c).

**Figure 5:**
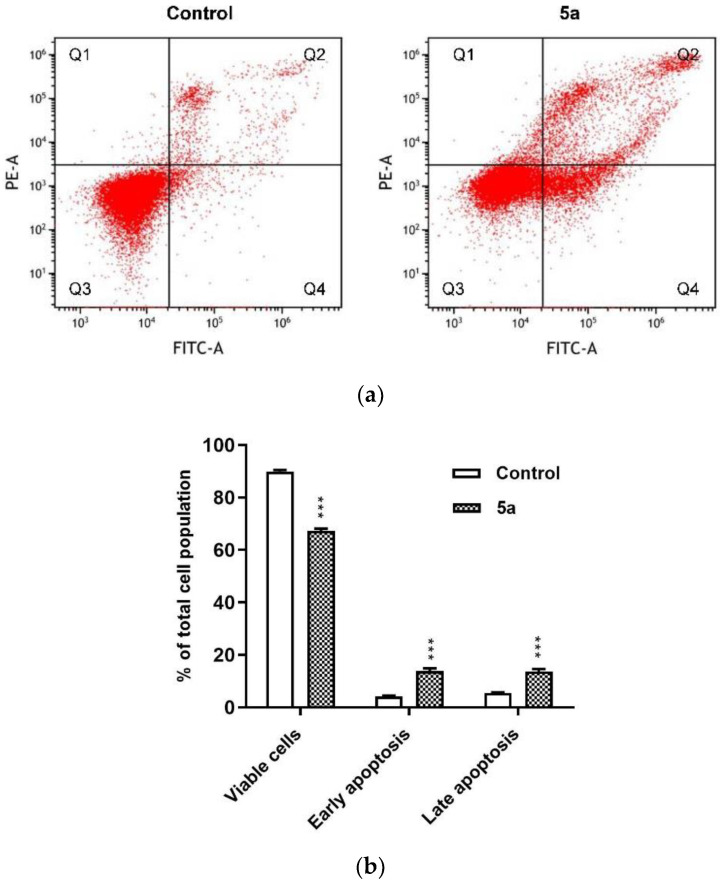
Induction of apoptosis in HL-60 cells treated with **5a** (1.8 µM) for 24 h: (**a**) Representative scattered blots of flow cytometry analysis of apoptosis by double-staining with FITC-Annexin-V and PI. The percentage of viable cells is shown in quadrant Q3. Quadrant Q4 indicates the percentage of early apoptotic cells (Annexin V-positive cells), whereas quadrant Q2 shows the percentage of late apoptotic/death cells (Annexin V-and PI-positive cells). (**b**) Quantitative analysis of apoptotic cell death by Annexin V and PI assay. Data are presented as mean ±SEM of three independent experiments. Statistical significance was assessed using Student’s *t* test. *** *p* < 0.001 vs. control.

Pro-apoptotic activity of **5a** was also confirmed using FITC-Annexin V and PI double-staining, based on the loss of plasma membrane asymmetry (phosphatidylserine externalization), which is a marker of the earlier stages of apoptosis. Flow cytometry analysis showed that **5a** (1.8 µM) increased early and late apoptotic cell fractions by up to 13.9% and 13.7% of the cell population, respectively (Figure 5).

#### 2.2.3. Influence of **5a** on ABCB1 Gene Expression

**Figure 6:**
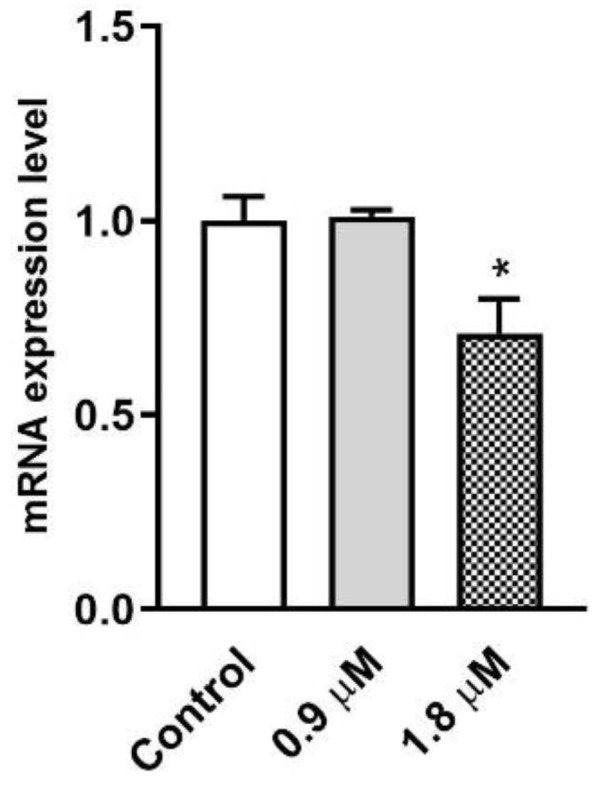
Real-time PCR analysis of *ABCB1* mRNA levels in HL-60 cells treated with **5a** for 24 h. Data are presented as mean ± SEM of three independent experiments. Statistical significance was assessed using one-way ANOVA and a post-hoc multiple comparison Student–Newman–Keuls test. * *p* < 0.05 vs. control.

Overexpression of ATP-binding cassette (ABC) transporters causing multidrug resistance (MDR) in cancer cells is one of the major problems associated with poor therapeutic outcome of anticancer drugs [19]. ABC transporters reduce the cellular uptake of drugs into cancer cells, defending them from medical interventions. In leukemia cells ABCB1 (P-glycoprotein), overexpression has been observed [20,21]. In our previous study [16], we demonstrated that some selected 3-methylidenequinolin-4-ones significantly decreased the expression of *ABCB1* gene in MCF-7 cells. We observed a 2-fold down regulation of this gene as compared with control, indicating that compounds with such a skeleton might have potential as ABCB1 transporter inhibitors, able to prevent MDR in MCF-7 cells. It seemed interesting to determine if 3-methylidenequinolin-4-one **5a** would also down-regulate *ABCB1* mRNA levels in leukemia HL-60 cells. Here, we tested the *ABCB1* gene expression of **5a** at two concentrations, 0.9 and 1.8 µM. Compound **5a** decreased the *ABCB1* mRNA level at a higher concentration (Figure 6).

## 3. Materials and Methods

### 3.1. Chemistry

#### 3.1.1. General

Reagents and starting materials were purchased from commercial vendors and used without further purification. All organic solvents were dried over appropriate drying agents and distilled prior to use. Standard syringe techniques were used for transferring dry solvents. NMR spectra were recorded on a Bruker UltraShield 700 instrument, running at 700 MHz for ^1^H, 176 MHz for ^13^C, and 283 MHz for ^31^P. Chemical shifts (δ) are reported in ppm relative to residual solvent signals (CDCl_3_: 7.26 ppm for ^1^H, 77.16 ppm for ^13^C NMR). ^31^P NMR spectra were recorded using broadband proton decoupling. Melting points were determined in open capillaries and are uncorrected. Column chromatography was performed on Aldrich^®^ silica gel 60 (230–400 mesh). Thin-layer chromatography was performed with precoated TLC sheets of silica gel 60 F254 (Aldrich^®^) and visualized by ultraviolet irradiation.

General procedures and characterization data for compounds **9a-e** and **10a-t** as well as ^1^H- and ^13^C NMR spectra of compounds **5a-t** are given in Supplementary Materials.

#### 3.1.2. Experimental Procedure for the Synthesis of 3-Methylidene-1-sulfonyl-2,3-dihydroquinolin-4(1H)-ones **5a-t**

A solution of the corresponding diethyl (4-hydroxy-1-sulfonyl-1,2-dihydro-3-yl)phosphonate **10a-t** (1.0 mmol) and potassium carbonate (414 mg, 3.0 mmol) in THF (10 mL) was stirred at room temperature for 30 min under argon atmosphere. Then, formaldehyde solution, 36% in water (410 µL, 5.0 mmol), was added in one portion, and the solution was heated to 50 °C. The mixture was stirred for 3 h when the 2-aryl substituted substrate was used, and for 16 h in the case of the 2-alkyl substituent. The reaction was quenched with brine (10 mL) and the mixture was extracted with ethyl acetate (3 × 20 mL). The organic layers were combined, washed with 10% potassium carbonate, water, and brine (30 mL) and were dried over MgSO_4_. The evaporation of the solvent gave crude product, which was purified by column chromatography (eluent: hexane/ethyl acetate 3:1).

2-Ethyl-3-methylidene-1-(phenylsulfonyl)-2,3-dihydroquinolin-4(1*H*)-one (**5a**). Yield: 210 mg (64%). White solid. *R*_f_ (hexane/ethyl acetate 3:1) 0.44, mp: 110 °C. ^1^H-NMR (700 MHz, CDCl_3_): δ 7.90 (ddd, *J* = 7.8, 1.7, 0.5 Hz, 1H), 7.84 (ddd, *J* = 8.2, 1.1, 0.5 Hz, 1H), 7.63 (ddd, *J* = 8.2, 7.3, 1.7 Hz, 1H), 7.45 (ddt, *J* = 8.7, 7.3, 1.1 Hz, 1H), 7.38–7.36 (m, 2H), 7.35–7.33 (m, H), 7.29–7.26 (m, 2H), 5.96 (d, *J* = 1.0 Hz, 1H), 5.22 (t, *J* = 1.0 Hz, 1H), 4.94 (dd, *J* = 9.2, 6.7 Hz, 1H), 1.68 (ddq, *J* = 14.4, 9.2, 7.3 Hz, 1H), 1.47 (ddd, *J* = 14.4, 7.3, 6.7 Hz, 1H), 0.97 (t, *J* = 7.3 Hz, 3H). ^13^C NMR (176 MHz, CDCl_3_): δ 181.92, 141.25, 139.74, 137.78, 134.72, 133.36, 129.00, 128.40, 128.22, 128.09, 127.70, 127.15, 124.07, 63.16, 28.44, 10.70. ESI-MS: 328.0 ([M+H]^+^). Anal. Calcd. for C_18_H_17_NO_3_S: C, 66.04; H, 5.23. Found: C, 66.19; H, 5.21.

3-Methylidene-1-(phenylsulfonyl)-2-(propan-2-yl)-2,3-dihydroquinolin-4(1*H*)-one (**5b**). Yield: 100 mg (29%). White solid. *R*_f_ (hexane/ethyl acetate 3:1) 0.49, mp: 86 °C. ^1^H-NMR (700 MHz, CDCl_3_): δ 7.90 (dd, *J* = 7.6, 1.7 Hz, 1H), 7.86 (dd, *J* = 8.2, 1.2 Hz, 1H), 7.63 (ddd, *J* = 8.2, 7.6, 1.7 Hz, 1H), 7.45 (tt, *J* = 7.4, 1.2 Hz, 1H), 7.40–7.35 (m, 2H), 7.34 (td, *J* = 7.6, 1.2 Hz, 1H), 7.29–7.26 (m, 2H), 5.99 (d, *J* = 1.1 Hz, 1H), 5.17 (t, *J* = 1.1 Hz, 1H), 4.54 (d, *J* = 10.3 Hz, 1H), 1.61 (dp, *J* = 10.3, 6.6 Hz, 1H), 1.08 (d, *J* = 6.6 Hz, 3H), 0.82 (d, *J* = 6.6 Hz, 3H). ^13^C NMR (176 MHz, CDCl_3_): δ 182.36, 140.19, 139.92, 138.15, 134.80, 133.31, 128.98, 128.32, 128.14, 128.04, 127.04, 125.35, 68.23, 30.64, 19.83, 19.35. Anal. Calcd. for C_19_H_19_NO_3_S: C, 66.84; H, 5.61. Found: C, 66.63; H, 5.52.

3-Methylidene-2-phenyl-1-(phenylsulfonyl)-2,3-dihydroquinolin-4(1*H*)-one (**5c**). Yield: 190 mg (51%). White solid. *R*_f_ (hexane/ethyl acetate 3:1) 0.49, mp: 150 °C. ^1^H-NMR (700 MHz, CDCl_3_): δ 7.83 (ddd, *J* = 9.4, 8.0, 1.3 Hz, 2H), 7.53 (ddd, *J* = 8.2, 7.3, 1.7 Hz, 1H), 7.50 (ddt, *J* = 8.7, 7.4, 1.3 Hz, 1H), 7.48–7.46 (m, 2H), 7.35–7.29 (m, 4H), 7.25–7.10 (m, 3H), 7.19–7.15 (m, 1H), 6.32 (s, 1H), 6.23 (d, *J* = 1.0 Hz, 1H), 5.43 (t, *J* = 1.0 Hz, 1H). ^13^C NMR (176 MHz, CDCl_3_): δ 182.08, 140.00, 138.86, 137.91, 137.41, 134.87, 133.54, 129.10, 128.65, 128.16, 128.14, 128.10, 128.08, 127.77, 127.08, 126.37, 63.63. Anal. Calcd. for C_22_H_17_NO_3_S: C, 70.38; H, 4.56. Found: C, 70.19; H, 4.50.

2-(4-Methoxyphenyl)-3-methylidene-1-(phenylsulfonyl)-2,3-dihydroquinolin-4(1*H*)-one (**5d**). Yield: 210 mg (52%). White solid. *R*_f_ (hexane/ethyl acetate 3:1) 0.36, mp: 152 °C. ^1^H-NMR (700 MHz, CDCl_3_): δ 7.84 (ddd, *J* = 7.8, 1.7, 0.5 Hz, 1H), 7.79 (ddd, *J* = 8.2, 1.2, 0.5 Hz, 1H), 7.53 (ddd, *J* = 8.2, 7.3, 1.7 Hz, 1H), 7.49 (ddt, *J* = 8.7, 7.6, 1.2 Hz, 1H), 7.47–7.44 (m, 2H), 7.34–7.31 (m, 2H), 7.24 (ddd, *J* = 7.8, 7.3, 1.1 Hz, 1H), 7.22–7.18 (m, 2H), 6.74–6.71 (m, 2H), 6.26 (s, 1H), 6.19 (d, *J* = 1.0 Hz, 1H), 5.39 (t, *J* = 1.0 Hz, 1H), 3.69 (s, 3H). ^13^C NMR (176 MHz, CDCl_3_): δ 182.20, 159.38, 139.97, 139.05, 137.95, 134.83, 133.51, 129.40, 129.09, 128.36, 128.22, 128.19, 128.03, 127.76, 127.05, 126.07, 114.01, 63.25, 55.27. Anal. Calcd. for C_23_H_19_NO_4_S: C, 68.13; H, 4.72. Found: C, 68.41; H, 4.83.

1-((4-Chlorophenyl)sulfonyl)-2-ethyl-3-methylidene-2,3-dihydroquinolin-4(1*H*)-one (**5e**). Yield: 340 mg (94%). White solid. *R*_f_ (hexane/ethyl acetate 3:1) 0.56, mp: 118 °C. ^1^H-NMR (700 MHz, CDCl_3_): δ 7.91 (dd, *J* = 7.8, 1.7 Hz, 1H), 7.82 (dd, *J* = 8.2, 1.1 Hz, 1H), 7.63 (ddd, *J* = 8.2, 7.3, 1.7 Hz, 1H), 7.36 (td, *J* = 7.5, 1.1 Hz, 1H), 7.32–7.28 (m, 2H), 7.26–7.23 (m, 2H), 6.03 (d, *J* = 1.0 Hz, 1H), 5.27 (t, *J* = 1.0 Hz, 1H), 4.94 (dd, *J* = 9.2, 6.6 Hz, 1H), 1.67 (ddq, *J* = 14.4, 9.2, 7.3 Hz, 1H), 1.47 (dp, *J* = 14.4, 7.3 Hz, 1H), 0.96 (t, *J* = 7.3 Hz, 3H). ^13^C NMR (176 MHz, CDCl_3_): δ 181.76, 141.22, 139.99, 139.40, 136.38, 134.82, 129.28, 129.07, 128.26, 128.13, 127.34, 124.24, 63.29, 28.39, 10.66. ESI-MS: 362.0 ([M+H]^+^). Anal. Calcd. for C_18_H_16_ClNO_3_S: C, 59.75; H, 4.46. Found: C, 59.54; H, 4.57.

1-((4-Chlorophenyl)sulfonyl)-3-methylidene-2-(propan-2-yl)-2,3-dihydroquinolin-4(1*H*)-one (**5f**). Yield: 300 mg (80%). White solid. *R*_f_ (hexane/ethyl acetate 3:1) 0.56, mp: 126 °C. ^1^H-NMR (700 MHz, CDCl_3_): δ 7.91 (dd, *J* = 7.6, 1.7 Hz, 1H), 7.83 (dd, *J* = 8.2, 1.1 Hz, 1H), 7.63 (ddd, *J* = 8.2, 7.6, 1.7 Hz, 1H), 7.35 (td, *J* = 7.6, 1.1 Hz, 1H), 7.31–7.29 (m, 2H), 7.26–7.23 (m, 2H), 6.06 (d, *J* = 1.0 Hz, 1H), 5.22 (t, *J* = 1.0 Hz, 1H), 4.53 (d, *J* = 10.3 Hz, 1H), 1.60 (dp, *J* = 10.3, 6.6 Hz, 1H), 1.06 (d, *J* = 6.6 Hz, 3H), 0.81 (d, *J* = 6.6 Hz, 3H). ^13^C NMR (176 MHz, CDCl_3_): δ 182.13, 139.89, 139.83, 139.76, 136.62, 134.87, 129.23, 129.01, 128.25, 128.17, 127.86, 127.21, 125.49, 68.30, 30.55, 19.75, 19.27. Anal. Calcd. for C_19_H_18_ClNO_3_S: C, 60.72; H, 4.83. Found: C, 60.65; H, 4.89.

1-((4-Chlorophenyl)sulfonyl)-3-methylidene-2-phenyl-1-(phenylsulfonyl)-2,3-dihydroquinolin-4(1*H*)-one (**5g**). Yield: 230 mg (56%). White solid. *R*_f_ (hexane/ethyl acetate 3:1) 0.54, mp: 178 °C. ^1^H-NMR (700 MHz, CDCl_3_): δ 7.85 (dd, *J* = 7.6, 1.7 Hz, 1H), 7.80 (dd, *J* = 8.2, 1.1 Hz, 1H), 7.54 (ddd, *J* = 8.2, 7.6, 1.7 Hz, 1H), 7.42–7.38 (m, 2H), 7.33–7.28 (m, 4H), 7.25 (td, *J* = 7.6, 1.1 Hz, 1H), 7.25–7.19 (m, 2H), 7.20–7.15 (m, 1H), 6.33 (s, 1H), 6.31 (d, *J* = 1.0 Hz, 1H), 5.48 (t, *J* = 1.0 Hz, 1H). ^13^C NMR (176 MHz, CDCl_3_): δ 181.95, 140.22, 139.70, 138.93, 137.20, 136.48, 134.97, 129.41, 129.16, 128.72, 128.27, 128.20, 128.10, 128.06, 127.30, 127.05, 126.50, 63.78. Anal. Calcd. for C_22_H_16_ClNO_3_S: C, 64.47; H, 3.93. Found: C, 64.66; H, 3.92.

1-((4-Chlorophenyl)sulfonyl)-2-(4-methoxyphenyl)-3-methylidene-2,3-dihydroquinolin-4(1*H*)-one (**5h**). Yield: 420 mg (95%). White solid. *R*_f_ (hexane/ethyl acetate 3:1) 0.35, mp: 140 °C. ^1^H-NMR (700 MHz, CDCl_3_): δ 7.85 (dd, *J* = 7.5, 1.7 Hz, 1H), 7.77 (dd, *J* = 8.3, 1.1 Hz, 1H), 7.53 (ddd, *J* = 8.3, 7.5, 1.7 Hz, 1H), 7.40–7.37 (m, 2H), 7.31–7.28 (m, 2H), 7.24 (td, *J* = 7.5, 1.1 Hz, 1H), 7.20–7.18 (m, 2H), 6.75–6.71 (m, 2H), 6.27 (s, 2H), 5.45 (d, *J* = 1.0 Hz, 1H), 3.68 (s, 3H). ^13^C NMR (176 MHz, CDCl_3_): δ 182.05, 159.44, 140.14, 139.64, 139.07, 136.50, 134.91, 129.37, 129.15, 129.13, 128.31, 128.18, 128.11, 128.09, 127.23, 126.20, 114.06, 63.37, 55.26. Anal. Calcd. for C_23_H_18_ClNO_4_S: C, 62.80; H, 4.12. Found: C, 62.83; H, 4.19.

2-Ethyl-6,7-dimethoxy-3-methylidene-1-tosyl-2,3-dihydroquinolin-4(1*H*)-one (**5i**). Yield: 170 mg (42%). White solid. *R*_f_ (hexane/ethyl acetate 3:1) 0.10, mp: 168 °C. ^1^H-NMR (700 MHz, CDCl_3_): δ 7.31 (s, 1H), 7.30 (s, 1H), 7.26–7.23 (m, 2H), 7.07–7.04 (m, 2H), 5.91 (d, *J* = 1.1 Hz, 1H), 5.17 (t, *J* = 1.1 Hz, 1H), 4.89 (dd, *J* = 9.0, 7.0 Hz, 1H), 4.01 (s, 3H), 3.90 (s, 3H), 2.30 (s, 3H), 1.70 (ddq, *J* = 14.6, 9.0, 7.0 Hz, 1H), 1.45 (dp, *J* = 14.2, 7.0 Hz, 1H), 0.96 (t, *J* = 7.0 Hz, 3H). ^13^C NMR (176 MHz, CDCl_3_): δ 180.88, 154.38, 148.23, 144.22, 141.15, 135.19, 134.60, 129.52, 127.73, 123.40, 121.15, 110.63, 108.43, 63.47, 56.64, 56.20, 28.36, 21.61, 10.78. ESI-MS: 402.0 ([M+H]^+^). Anal. Calcd. for C_21_H_23_NO_5_S: C, 62.83; H, 5.77. Found: C, 62.87; H, 5.83.

6,7-Dimethoxy-3-methylidene-2-(propan-2-yl)-1-tosyl-2,3-dihydroquinolin-4(1*H*)-one (**5j**). Yield: 350 mg (85%). White solid. *R*_f_ (hexane/ethyl acetate 3:1) 0.10, mp: 150 °C. ^1^H-NMR (700 MHz, CDCl_3_): δ 7.31 (s, 1H), 7.30 (s, 1H), 7.25–7.23 (m, 2H), 7.06–7.03 (m, 2H), 5.92 (d, *J* = 1.3 Hz, 1H), 5.12 (dd, *J* = 1.3, 0.7 Hz, 1H), 4.48 (d, *J* = 10.1 Hz, 1H), 4.01 (s, 3H), 3.88 (s, 3H), 2.29 (s, 3H), 1.63 (dhept, *J* = 10.1, 6.6 Hz, 1H), 1.06 (d, *J* = 6.6 Hz, 3H), 0.78 (d, *J* = 6.6 Hz, 3H). ^13^C NMR (176 MHz, CDCl_3_): δ 181.30, 154.39, 148.12, 144.11, 139.77, 135.57, 134.87, 129.46, 127.65, 124.66, 121.20, 110.21, 108.40, 68.46, 56.64, 56.16, 30.52, 21.57, 19.99, 19.36. Anal. Calcd. for C_22_H_25_NO_5_S: C, 63.60; H, 6.06. Found: C, 63.56; H, 6.04.

6,7-Dimethoxy-3-methylidene-2-phenyl-1-tosyl-2,3-dihydroquinolin-4(1*H*)-one (**5k**). Yield: 340 mg (76%). Pale yellow solid. *R*_f_ (hexane/ethyl acetate 3:1) 0.12, mp: 196 °C. ^1^H-NMR (700 MHz, CDCl_3_): δ 7.38–7.33 (m, 2H), 7.31 (dt, *J* = 8.1, 1.2 Hz, 2H), 7.29 (s, 1H), 7.25 (s, 1H), 7.25–7.20 (m, 2H), 7.20–7.15 (m, 1H), 7.14–7.10 (m, 2H), 6.26 (s, 1H), 6.18 (d, *J* = 1.1 Hz, 1H), 5.39 (t, *J* = 1.1 Hz, 1H), 3.97 (s, 3H), 3.83 (s, 3H), 2.34 (s, 3H). ^13^C NMR (176 MHz, CDCl_3_): δ 181.06, 154.48, 148.15, 144.48, 138.86, 137.89, 135.46, 134.73, 129.63, 128.00, 127.81, 127.02, 125.71, 121.28, 110.35, 108.34, 63.91, 56.60, 56.07, 21.65. Anal. Calcd. for C_25_H_23_NO_5_S: C, 66.80; H, 5.16. Found: C, 66.96; H, 5.18.

6,7-Dimethoxy-2-(4-methoxyphenyl)-3-methylidene-1-tosyl-2,3-dihydroquinolin-4(1*H*)-one (**5l**). Yield: 360 mg (75%). Pale yellow solid. *R*_f_ (hexane/ethyl acetate 3:1) 0.06, mp: 196 °C. ^1^H-NMR (700 MHz, CDCl_3_): δ 7.37–7.32 (m, 2H), 7.26 (s, 1H), 7.25 (s, 1H), 7.23–7.19 (m, 2H), 7.14–7.09 (m, 2H), 6.76–6.72 (m, 2H), 6.21 (s, 1H), 6.15 (d, *J* = 1.1 Hz, 1H), 5.35 (t, *J* = 1.1 Hz, 1H), 3.97 (s, 3H), 3.84 (s, 3H), 3.70 (s, 3H), 2.34 (s, 3H). ^13^C NMR (176 MHz, CDCl_3_): δ 181.18, 159.32, 154.45, 148.12, 144.43, 139.04, 135.41, 134.76, 129.91, 129.62, 128.29, 127.80, 125.45, 121.32, 113.93, 110.42, 108.31, 63.52, 56.60, 56.08, 55.26, 21.66. Anal. Calcd. for C_26_H_25_NO_6_S: C, 65.12; H, 5.25. Found: C, 65.21; H, 5.29.

6-Chloro-2-ethyl-3-methylidene-1-tosyl-2,3-dihydroquinolin-4(1*H*)-one (**5m**). Yield: 50 mg (13%). White solid. *R*_f_ (hexane/ethyl acetate 3:1) 0.53, mp: 126 °C. ^1^H-NMR (700 MHz, CDCl_3_): δ 7.88 (d, *J* = 2.6 Hz, 1H), 7.83 (d, *J* = 8.7 Hz, 1H), 7.59 (dd, *J* = 8.7, 2.6 Hz, 1H), 7.31–7.27 (m, 2H), 7.14–7.10 (m, 2H), 6.03–6.00 (m, 1H), 5.28 (t, *J* = 0.9 Hz, 1H), 4.94 (dd, *J* = 9.2, 6.7 Hz, 1H), 2.35 (s, 3H), 1.69 (ddq, *J* = 14.4, 9.2, 7.3 Hz, 1H), 1.48 (ddd, *J* = 14.4, 7.3, 6.7 Hz, 1H), 0.99 (t, *J* = 7.3 Hz, 4H). ^13^C NMR (176 MHz, CDCl_3_): δ 181.05, 144.60, 140.79, 138.36, 134.71, 134.59, 133.15, 129.79, 129.76, 129.08, 127.74, 127.72, 124.76, 63.05, 28.43, 21.68, 10.68. ESI-MS: 375.9 ([M+H]^+^). Anal. Calcd. for C_19_H_18_ClNO_3_S: C, 60.72; H, 4.83. Found: C, 60.67; H, 4.94.

6-Chloro-3-methylidene-2-(propan-2-yl)-1-tosyl-2,3-dihydroquinolin-4(1*H*)-one (**5n**). Yield: 130 mg (33%). White solid. *R*_f_ (hexane/ethyl acetate 3:1) 0.56, mp: 130 °C. ^1^H-NMR (700 MHz, CDCl_3_): δ 7.86 (d, *J* = 2.6 Hz, 1H), 7.82 (d, *J* = 8.7 Hz, 1H), 7.57 (dd, *J* = 8.7, 2.6 Hz, 1H), 7.29–7.24 (m, 2H), 7.11–7.08 (m, 2H), 6.02 (d, *J* = 1.1 Hz, 1H), 5.21 (t, *J* = 1.1 Hz, 1H), 4.53 (d, *J* = 10.2 Hz, 1H), 2.33 (s, 3H), 1.59 (dp, *J* = 10.2, 6.6 Hz, 1H), 1.06 (d, *J* = 6.6 Hz, 3H), 0.81 (d, *J* = 6.6 Hz, 3H). ^13^C NMR (176 MHz, CDCl_3_): δ 181.44, 144.51, 139.41, 138.73, 135.00, 134.63, 133.01, 129.72, 129.39, 129.13, 127.74, 127.66, 126.05, 68.03, 30.62, 21.66, 19.79, 19.28. Anal. Calcd. for C_20_H_20_ClNO_3_S: C, 61.61; H, 5.17. Found: C, 61.49; H, 5.25.

6-Chloro-3-methylidene-2-phenyl-1-tosyl-2,3-dihydroquinolin-4(1*H*)-one (**5o**). Yield: 220 mg (52%). White solid. *R*_f_ (hexane/ethyl acetate 3:1) 0.58, mp: 162 °C. ^1^H-NMR (700 MHz, CDCl_3_): δ 7.79 (d, *J* = 2.6 Hz, 1H), 7.78 (d, *J* = 8.8 Hz, 1H), 7.47 (dd, *J* = 8.7, 2.6 Hz, 1H), 7.38–7.35 (m, 2H), 7.28 (dt, *J* = 8.3, 1.2 Hz, 2H), 7.25–7.22 (m, 2H), 7.22–7.18 (m, 1H), 7.17–7.14 (m, 2H), 6.30 (s, 1H), 6.26 (d, *J* = 1.0 Hz, 1H), 5.47 (t, *J* = 1.0 Hz, 1H), 2.37 (s, 3H). ^13^C NMR (176 MHz, CDCl_3_): δ 181.22, 144.81, 138.60, 138.42, 137.16, 134.82, 134.71, 133.05, 129.85, 129.54, 129.01, 128.76, 128.26, 127.79, 127.70, 127.05, 127.00, 63.49, 21.68. Anal. Calcd. for C_23_H_18_ClNO_3_S: C, 65.17; H, 4.28. Found: C, 64.98; H, 4.39.

6-Chloro-2-(4-methoxyphenyl)-3-methylidene-1-tosyl-2,3-dihydroquinolin-4(1*H*)-one (**5p**). Yield: 340 mg (75%). White solid. *R*_f_ (hexane/ethyl acetate 3:1) 0.36, mp: 150°C. ^1^H-NMR (700 MHz, CDCl_3_): δ 7.78 (d, *J* = 2.6 Hz, 1H), 7.75 (d, *J* = 8.7 Hz, 1H), 7.46 (dd, *J* = 8.7, 2.6 Hz, 1H), 7.37–7.34 (m, 2H), 7.19–7.16 (m, 2H), 7.15–7.13 (m, 2H), 6.76–6.72 (m, 2H), 6.24 (d, *J* = 1.0 Hz, 1H), 6.22 (d, *J* = 1.0 Hz, 1H), 5.43 (t, *J* = 1.0 Hz, 1H), 3.70 (s, 3H), 2.35 (s, 3H). ^13^C NMR (176 MHz, CDCl_3_): δ 181.35, 159.50, 144.76, 138.61, 138.57, 134.87, 134.66, 133.00, 129.83, 129.59, 129.10, 129.05, 128.33, 127.78, 127.64, 126.70, 114.12, 63.12, 55.30, 21.68. Anal. Calcd. for C_24_H_20_ClNO_4_S: C, 63.50; H, 4.44. Found: C, 63.37; H, 4.53.

6. -Bromo-2-ethyl-3-methylidene-1-tosyl-2,3-dihydroquinolin-4(1*H*)-one (**5q**). Yield: 270 mg (64%). White solid. *R*_f_ (hexane/ethyl acetate 3:1) 0.52, mp: 132 °C. ^1^H-NMR (700 MHz, CDCl_3_): δ 8.02 (dd, *J* = 2.3, 0.5 Hz, 1H), 7.74 (dd, *J* = 8.7, 0.5 Hz, 1H), 7.72 (dd, *J* = 8.7, 2.3 Hz, 1H), 7.29–7.26 (m, 2H), 7.12–7.09 (m, 2H), 5.99 (d, *J* = 1.0 Hz, 1H), 5.26 (t, *J* = 1.0 Hz, 1H), 4.92 (dd, *J* = 9.2, 6.7 Hz, 1H), 2.34 (s, 3H), 1.67 (ddq, *J* = 14.4, 9.2, 7.3 Hz, 1H), 1.47 (ddd, *J* = 14.4, 7.3, 6.7 Hz, 1H), 0.97 (t, *J* = 7.3 Hz, 3H). ^13^C NMR (176 MHz, CDCl_3_): δ 180.98, 144.63, 140.85, 138.91, 137.50, 134.80, 130.84, 129.96, 129.80, 129.32, 127.74, 124.74, 120.85, 63.07, 28.45, 21.68, 10.68. ESI-MS: 419.9 ([M+H]^+^). Anal. Calcd. for C_19_H_18_BrNO_3_S: C, 54.29; H, 4.32. Found: C, 54.32; H, 4.30.

6-Bromo-3-methylidene-2-(propan-2-yl)-1-tosyl-2,3-dihydroquinolin-4(1*H*)-one (**5r**). Yield: 210 mg (48%). White solid. *R*_f_ (hexane/ethyl acetate 3:1) 0.56, mp: 118 °C. ^1^H-NMR (700 MHz, CDCl_3_): δ 8.01 (d, *J* = 2.4 Hz, 1H), 7.76 (d, *J* = 8.7 Hz, 1H), 7.71 (dd, *J* = 8.7, 2.4 Hz, 1H), 7.30–7.25 (m, 2H), 7.12–7.08 (m, 2H), 6.02 (d, *J* = 1.1 Hz, 1H), 5.21 (t, *J* = 1.1 Hz, 1H), 4.53 (d, *J* = 10.2 Hz, 1H), 2.33 (s, 3H), 1.60 (dp, *J* = 10.2, 6.6 Hz, 1H), 1.06 (d, *J* = 6.6 Hz, 3H), 0.82 (d, *J* = 6.6 Hz, 3H). ^13^C NMR (176 MHz, CDCl_3_): δ 181.37, 144.54, 139.47, 139.28, 137.54, 135.08, 130.84, 129.75, 129.57, 129.38, 127.68, 126.01, 120.70, 68.05, 30.68, 21.67, 19.81, 19.30. Anal. Calcd. for C_20_H_20_BrNO_3_S: C, 55.31; H, 4.64. Found: C, 55.38; H, 4.63.

6-Bromo-3-methylidene-2-phenyl-1-tosyl-2,3-dihydroquinolin-4(1*H*)-one (**5s**). Yield: 240 mg (51%). White solid. *R*_f_ (hexane/ethyl acetate 3:1) 0.57, mp: 162 °C. ^1^H-NMR (700 MHz, CDCl_3_): δ 7.94 (d, *J* = 2.4 Hz, 1H), 7.71 (d, *J* = 8.7 Hz, 1H), 7.62 (dd, *J* = 8.7, 2.4 Hz, 1H), 7.38–7.35 (m, 2H), 7.28 (ddt, *J* = 7.6, 2.4, 1.0 Hz, 2H), 7.24 (ddd, *J* = 7.6, 6.7, 1.3 Hz, 2H), 7.22–7.18 (m, 1H), 7.17–7.14 (m, 2H), 6.29 (t, *J* = 0.9 Hz, 1H), 6.25 (d, *J* = 1.0 Hz, 1H), 5.46 (t, *J* = 0.9 Hz, 1H), 2.37 (s, 3H). ^13^C NMR (176 MHz, CDCl_3_): δ 181.17, 144.87, 139.16, 138.52, 137.63, 137.20, 134.93, 130.82, 129.90, 129.73, 129.27, 128.82, 128.32, 127.83, 127.10, 126.96, 120.83, 63.54, 21.73. Anal. Calcd. for C_23_H_18_BrNO_3_S: C, 58.98; H, 3.87. Found: C, 58.79; H, 3.96.

6-Bromo-2-(4-methoxyphenyl)-3-methylidene-1-tosyl-2,3-dihydroquinolin-4(1*H*)-one (**5t**). Yield: 190 mg (38%). White solid. *R*_f_ (hexane/ethyl acetate 3:1) 0.38, mp: 168 °C. ^1^H-NMR (700 MHz, CDCl_3_): δ 7.94 (d, *J* = 2.4 Hz, 1H), 7.69 (d, *J* = 8.7 Hz, 1H), 7.61 (dd, *J* = 8.7, 2.4 Hz, H), 7.38–7.34 (m, 2H), 7.20–7.15 (m, 2H), 7.16–7.14 (m, 2H), 6.77–6.72 (m, 2H), 6.24 (s, 1H), 6.22 (d, *J* = 1.0 Hz, 1H), 5.43 (t, *J* = 1.0 Hz, 1H), 3.71 (s, 3H), 2.36 (s, 3H). ^13^C NMR (176 MHz, CDCl_3_): δ 181.27, 159.56, 144.79, 139.11, 138.66, 137.56, 134.95, 130.74, 129.86, 129.76, 129.29, 129.13, 128.36, 127.80, 126.66, 120.75, 114.17, 63.14, 55.33, 21.70. Anal.Calcd. for C_24_H_20_BrNO_4_S: C, 57.84; H, 4.05. Found: C, 57.71; H, 4.09.

### 3.2. Biology

#### 3.2.1. Cell Lines and Cell Culture

The promyelocytic leukemia HL-60 and breast cancer adenocarcinoma MCF-7 cell lines were purchased from the European Collection of Cell Cultures (ECACC). Leukemia cells were cultured in RPMI 1640 plus GlutaMax I medium (Gibco/Life Technologies, Carlsbad, CA, USA). MCF-7 cells were maintained in Minimum Essential Medium Eagle (Sigma Aldrich, St. Louis, MO, USA) and supplemented with 2 mM glutamine and men non-essential amino acid solution (Sigma Aldrich, St. Louis, MO, USA). Both media were supplemented with 10% heat-inactivated fetal bovine serum (Biological Industries, Beit-Haemek, Israel) and antibiotics (100 U/mL penicillin and 100 μg/mL streptomycin) (Sigma-Aldrich, St. Louis, MO, USA). Human umbilical vein endothelial cells (HUVECs) were purchased from the American Type Culture Collection (ATCC). Cells were cultured using EGM-2 Endothelial Medium BulletKit purchased from Lonza (Lonza, Walkersville, MD, USA). Cells were maintained at 37 °C in 5% CO_2_ atmosphere and grown until 80% confluent.

The tested compounds were dissolved in sterile dimethyl sulfoxide (DMSO) and further diluted with culture medium. The final concentration of DMSO in cell cultures was less than 0.1% *v*/*v*. Controls without and with 0.1% DMSO were performed in each experiment. At the used concentration, DMSO had no effect on the observed parameters.

#### 3.2.2. In Vitro Cytotoxicity Assay (MTT)

The MTT (3-(4,5-dimethylthiazol-2-yl)-2,5-diphenyltetrazolium bromide) assay, which measures the activity of cellular dehydrogenases, was performed according to the Mosmann method [22]. Exponentially growing cells were seeded into 24-well plates at a density of 8 × 10^4^ cells/mL and left to grow for 24 h. After 24 h or 48 h of incubation with various concentrations of the tested compounds (dissolved in DMSO and diluted with complete culture medium), cells were incubated with 0.1 mL of MTT solution (5 mg/mL in phosphate buffered saline) for 1.5 h. The metabolically active cells reduced MTT to blue formazan crystals. The plates were centrifuged, and the supernatant was discarded. DMSO (1 mL) was added to each well to dissolve the crystals, whose absorbance was measured at 560 nm using FlexStation 3 Multi-Mode Microplate Reader (Molecular Devices, LLC, San Jose, CA, USA). The untreated cells were used as control. The IC_50_ values were calculated from the concentration–response curves. The data were expressed as mean ± SEM of three independent experiments.

#### 3.2.3. Analysis of Apoptosis by Flow Cytometry Using FITC-Annexin V and Propidium Iodide (PI) Double-Staining

Apoptotic cell death was determined using FITC-Annexin V Apoptosis Detection Kit I (BD Bioscience, San Jose, CA, USA). Briefly, HL-60 cells were seeded in the 6-well plates (4.0 × 10^5^ cells/well) in 2 mL of the culture medium and treated with **10a** for 24 h. Afterwards, the cells were collected by centrifugation (300× *g*, 5 min), washed with PBS, re-suspended in binding buffer and stained with FITC-conjugated annexin V and propidium iodide (PI) according to the manufacturer guidelines (15 min, at room temperature). The samples were analyzed by flow cytometry using CytoFLEX (Beckman Coulter, Inc., Brea, CA, USA). Data analysis was performed using Kaluza Analysis Software v2 (Beckman Coulter, Inc., Brea, CA, USA).

#### 3.2.4. Analysis of Cell Proliferation, Apoptosis, and DNA Damage by Flow Cytometry

Cell proliferation, DNA damage, and apoptotic cell death were determined using Apoptosis, DNA Damage, and Cell Proliferation Kit (BD Bioscience, San Jose, CA, USA), according to the manufacturer protocol. Briefly, HL-60 cells were seeded in 6-well plates (4.0 × 10^5^ cells/mL) in 2 mL of growth medium and treated with **5a** for 24 h. Afterwards, the bromodeoxyuridine (BrdU, 10 µM) was added and the cells were incubated for additional 8 h. Next, the cells were collected by centrifugation (300× *g*, 5 min), counted, fixed, and permeabilized according to the manufacturer’s instructions. The cells were incubated with DNase (300 µg/mL in PBS) for 1 h at 37 °C and then simultaneously stained with fluorochrome-labeled anti-BrdU, H2AX (pS139), and cleaved PARP (Asp214) antibodies, for 20 min at room temperature. After washing, DNA staining with DAPI (1 µg/mL of the staining buffer) was performed, and the cells were analyzed by flow cytometry using CytoFLEX (Beckman Coulter, Inc., Brea, CA, USA).

#### 3.2.5. Real-Time PCR

The mRNA level of the *ABCB1* gene was assessed using quantitative real-time PCR. Briefly, HL-60 cells were seeded in 6-well plates (4.0 × 10^5^ cells/mL) in 2 mL of growth medium and treated with **5a** for 24 h. Total RNA was extracted using the Total RNA Mini Kit (A&A Biotechnology, Gdynia, Poland) according to the manufacturer’s protocol. The concentration of RNA used for further experiments was 500 ng. cDNA was synthesized using Transcriba Kit (A&A Biotechnology, Gdynia, Poland). The amplification of cDNA was performed using RT PCR Mix SYBR (A&A Biotechnology, Gdynia, Poland) and gene specific primers (Table 3) in the Stratagene Mx3005P QPCR System (Agilent Technologies, Inc. Santa Clara, CA, USA) according to the manufacturer’s guidelines. Glyceraldehyde 3-phosphate dehydrogenase (*GAPDH*) was used as a reference gene. The expression level of *ABCB1* gene was determined by the 2^−∆∆CT^ method [23].

#### 3.2.6. Statistical Analysis

All statistical calculations were performed using Prism 5.0 software (GraphPad Software Inc., San Diego, CA, USA). Results from three independent experiments performed in triplicate were expressed as mean ± SEM. Statistical significance was assessed using Student’s *t*-test (for comparisons of two treatment groups) or one-way ANOVA followed by a post-hoc multiple comparison Student–Newman–Keuls test (for comparisons of three or more groups). *p*-values < 0.05 were considered statistically significant.

## 4. Conclusions

Quinolinones have been known for years as efficient, broad spectrum antibiotics. In the last decade, yet another important activity of these compounds, their ability to inhibit cancer cell proliferation, has been investigated. In order to obtain a wide spectrum of diversely substituted quinolinone derivatives, the development of synthetic methods leading to such compounds has been pursued. Here, we used the well-recognized Horner–Wadsworth–Emmons (HWE) olefination in order to introduce the *exo*-methylidene group onto the heterocyclic ring of dihydroquinolin-4(1*H*)-ones. The α,β-unsaturated carbonyl function is a structural element responsible for interaction with various cellular proteins, which disturbs many cellular processes.

The aim of this study was to establish the structure–activity relationship of the new series of dihydroquinolin-4(1*H*)-ones. Using the routine MTT assay, the cytotoxicity of the analogs was assessed on two cancer cell types: an adherent breast cancer MCF-7 and a non-adherent leukemia HL-60. All new analogs were more cytotoxic for HL-60 than for MCF-7 cells. Analysis of the structure–activity relationship revealed that introducing any groups into the aromatic ring (R^1^ and R^2^) was not advantageous for activity. The substituent on the nitrogen atom (R^3^) did not significantly affect the activity of the tested compounds. The most important turned out to be position 2 (R^4^). The quinolin-4(1*H*)-ones with an alkyl substituent in that position were more potent than those bearing an aryl group. In general, *iso*-propyl as R^4^ generated more cytotoxic analogs than ethyl.

The ideal anticancer drug candidates should be highly cytotoxic for cancer cells but much less for normal, healthy cells. The selectivity of the new analogs, expressed as the IC_50_ ratio of HUVEC/HL60 cells, was calculated for the selected analogs, and values between 1 and 5.5 were obtained. These values showed that very high cytotoxicity against cancer cells did not go hand-in-hand with high selectivity. However, even approved drugs used for cancer treatment are in most cases strongly cytotoxic for normal cells. The difference between healthy and cancer cells is that the latter proliferate much faster and therefore are more susceptible to the action of drugs. The analog **5a**, 2-ethyl-3-methylidene-1-phenylsulfonyl-2,3-dihydroquinolin-4(1*H*)-one, which was 5-fold more cytotoxic for HL-60 than for HUVEC cells, was further evaluated in terms of its mode of action. This compound inhibited proliferation and induced apoptosis in HL-60 cells, which could be attributed to its ability to induce DNA damage. Additionally, the **5a** down-regulated ABCB1 mRNA level decreased the risk of MDR development. Based on these findings, the described structurally diversified dihydroquinolin-4(1*H*)-ones can serve as lead structures and can be further optimized with the purpose of finding new possibilities for cancer treatment.

## Figures and Tables

**Table 1:** Yields of *trans*- and *cis*-3-(diethoxyphosphoryl)-1-sulfonyl-2,3-dihydroquinolin-4-ones *trans*- and *cis*-**10a-t**, 3-(diethoxyphosphoryl)-1-sulfonyl-2,3-dihydroquinolin-4(1*H*)-ols *enol*-**10a-t**, and 3-methylidene-1-sulfonyl-2,3-dihydroquinolin-4(1*H*)-ones **5a-t**.

Compound	R^1^	R^2^	R^3^	R^4^	10		5
					*trans*-10/*cis*-10/ *enol*-10 ^1^	Yield [%] ^2^	Yield [%] ^2^
**a**	H	H	Phenyl	Et	2.0/4.0/94.0	46	64
**b**				*i*-Pr	2.0/1.0/97.0	72	29
**c**				Phenyl	1.0/–/99.0	71	51
**d**				4-Methoxyphenyl	1.0/–/99.0	87	52
**e**	H	H	4-Chlorophenyl	Et	1.5/2.5/96.0	80	94
**f**				*i*-Pr	2.5/0.5/97.0	54	80
**g**				Phenyl	1.0/–/99.0	73	56
**h**				4-Methoxyphenyl	1.0/–/99.0	77	95
**i**	OMe	OMe	4-Methylphenyl	Et	5.0/25.0/70.0	65	42
**j**				*i*-Pr	8.0/3.0/89.0	51	85
**k**				Phenyl	3.0/2.0/95.0	69	76
**l**				4-Methoxyphenyl	2.0/2.0/96.0	57	75
**m**	Cl	H	4-Methylphenyl	Et	1.0/2.0/97.0	88	13
**n**				*i*-Pr	1.5/0.5/98.0	85	33
**o**				Phenyl	1.0/–/99.0	75	52
**p**				4-Methoxyphenyl	1.0/–/99.0	63	75
**q**	Br	H	4-Methylphenyl	Et	1.0/3.0/96.0	60	64
**r**				*i*-Pr	1.5/0.5/98.0	77	48
**s**				Phenyl	1.0/–/99.0	76	51
**t**				4-Methoxyphenyl	1.0/–/99.0	73	38

^1^ Determined from ^31^P-NMR spectra of the product. ^2^ Yield of pure isolated product, based on **9** and **10**, respectively.

**Table 2:** In vitro cytotoxic activity of 3-methylidene-1-sulfonyl-2,3-dihydroquinolin-4(1*H*)-ones **5a-t** tested on two cancer (MCF-7, HL-60) and one normal (HUVEC) cell lines.

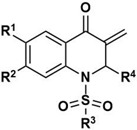 5a-t								
Compound	R^1^	R^2^	R^3^	R^4^	IC_50_ [µM] ^1^			
					MCF-7	HL-60	HUVEC	HUVEC/HL-60 IC_50_ Ratio
**5a**	H	H	Phenyl	Et	2.74 ± 0.07	0.34 ± 0.03	1.87 ± 0.02	5.50
**5b**				*i*-Pr	0.83 ± 0.02	0.29 ± 0.02	0.78 ± 0.21	2.67
**5c**				Phenyl	2.25 ± 0.20	1.36 ± 0.13		
**5d**				4-Methoxyphenyl	1.21 ± 0.24	1.12 ± 0.37		
**5e**	H	H	4-Chlorophenyl	Et	1.61 ± 0.07	0.37 ± 0.05	0.75 ± 0.00	2.03
**5f**				*i*-Pr	0.86 ± 0.02	0.23 ± 0.01	0.99 ± 0.07	4.30
**5g**				Phenyl	1.89 ± 0.03	0.78 ± 0.07		
**5h**				4-Methoxyphenyl	2.10 ± 0.16	0.92 ± 0.01		
**5i**	OMe	OMe	4-Methylphenyl	Et	1.01 ± 0.05	0.37 ± 0.02	0.42 ± 0.08	1.14
**5j**				*i*-Pr	1.44 ± 0.07	0.43 ± 0.02	0.99 ± 0.07	2.30
**5k**				Phenyl	1.46 ± 0.09	0.58 ± 0.02		
**5l**				4-Methoxyphenyl	1.15 ± 0.04	1.48 ± 0.06		
**5m**	Cl	H	4-Methylphenyl	Et	3.48 ± 0.42	1.75 ± 0.03		
**5n**				*i*-Pr	1.65 ± 0.05	0.67 ± 0.01	0.68 ± 0.03	1.01
**5o**				Phenyl	3.67 ± 0.41	2.04 ± 0.05		
**5p**				4-Methoxyphenyl	3.60 ± 0.19	2.07 ± 0.02		
**5q**	Br	H	4-Methylphenyl	Et	1.83 ± 0.15	0.61 ± 0.04	1.00 ± 0.22	1.64
**5r**				*i*-Pr	0.83 ± 0.02	0.19 ± 0.03	0.47 ± 0.03	2.47
**5s**				Phenyl	2.67 ± 0.05	0.62 ± 0.01		
**5t**				4-Methoxyphenyl	2.81 ± 0.05	0.74 ± 0.06		
Carboplatin					2.90 ± 0.10	3.80 ± 0.45	5.35± 0.05	1.41

^1^ Compound concentration required to inhibit metabolic activity by 50%. Values are expressed as mean ± SEM from concentration–response curves of at least three experiments using a nonlinear estimation (quasi-Newton algorithm) method.

**Table 3:** Primer sequences for real-time PCR reaction.

Gene	Primer Sequences	
	Forward Primer	Reverse Primer
*GAPDH*	5′ GTCGCTGTTGAAGTCAGAGGAG 3′	5′ CGTGTCAGTGGTGGACCTGAC 3′
*ABCB1*	5′ GTGGGGCAAGTCAGTTCATT 3′	5′ TCTTCACCTCCAGGCTCAGT 3′

## Data Availability

Not applicable.

## Supplementary Materials

The following supporting information can be downloaded at: https://www.mdpi.com/article/10.3390/molecules27113597/s1, general procedures and characterization data for compounds **9a-e** and **10a-t**, ^1^H- and ^13^C-NMR spectra of compounds **5a-t**.

## Sample Availability

Samples of the compounds are available from the authors.
