# Supplementary material for: Synthesis and Anticancer Properties of New 3-Methylidene-1-sulfonyl-2,3-dihydroquinolin-4(1H)-ones
Source: Molecules. 2022 Jun 3;27(11):3597. doi: 10.3390/molecules27113597 (PMC9181899; doi:10.3390/molecules27113597)

Synthesis and Anticancer Properties of New 3-Methylidene-1-sulfonyl-2,3-dihydroquinolin-4(1*H*)-ones

Agata Jaskulska [1], Katarzyna Gach-Janczak [2], Joanna Drogosz-Stachowicz [2], Tomasz Janecki [1*] and Anna Ewa Janecka [2,*]

[1] Institute of Organic Chemistry, Faculty of Chemistry, Lodz University of Technology, Żeromskiego 116, 90-924, Lodz, Poland; agata.jaskulska@dokt.p.lodz.pl (A.J.), tomasz.janecki@p.lodz.pl (T.J.)
[2] Department of Biomolecular Chemistry, Faculty of Medicine, Medicinal University of Lodz, Mazowiecka 6/8, 92-215, Lodz, Poland, katarzyna.gach@umed.lodz.pl (K.G.-J.), joanna.drogosz@stud.umed.lodz.pl (J. D.-S.), anna.janecka@umed.lodz.pl (A.E. J.)
* Correspondence: anna.janecka@umed.lodz.pl (A.E.J); tomasz.janecki@p.lodz.pl (T.J); Tel.: +48-422725706 (A.E.J); +48-426313220 (T.J.)

Supporting Information

Table of content

1. General information 2

2. General procedures and characterization data 2

2.1. Synthesis of diethyl (2-(2-(sulfonamido)phenyl)-2-oxoethyl)phosphonate (**9a-e**) 2

2.2. Synthesis of diethyl (4-hydroxy-1-sulfonyl-1,2-dihydroquinolin-3-yl)phosphonate (**10a-t**) 3

3. Copies of NMR spectra of 3-methylidene-1-sulfonyl-2,3-dihydroquinolin-4(1*H*)-ones (5a-t) 8

1. General information

Reagents and starting materials were purchased from commercial vendors and used without further purification. All organic solvents were dried over appropriate drying agents and distilled prior to use. Standard syringe techniques were used for transferring dry solvents. NMR spectra were recorded on a Bruker UltraShield 700 instrument, running at 700 MHz for ^{1}H, 176 MHz for ^{13}C, and 283 MHz for ^{31}P. Chemical shifts (δ) are reported in ppm relative to residual solvent signals ($CDCl_3$: 7.26 ppm for ^{1}H, 77.16 ppm for ^{13}C NMR). ^{31}P NMR spectra were recorded using broadband proton decoupling. Melting points were determined in open capillaries and are uncorrected. Column chromatography was performed on Aldrich® silica gel 60 (230–400 mesh). Thin-layer chromatography was performed with precoated TLC sheets of silica gel 60 F254 (Aldrich®) and visualized by ultraviolet irradiation.

2. General procedures and characterization data

2.1. *Synthesis of diethyl (2-(2-(sulfonamido)phenyl)-2-oxoethyl)phosphonate* (**9a-e**)

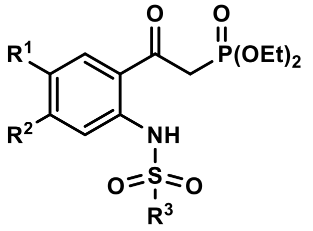

A solution of diisopropylamine (14 mL, 10.1 g, 99.9 mmol, 3.33 eq.) in 2-MeTHF (25.0 mL) under argon atmosphere was cooled to -50°C and *n*-butyllithium (45 mL of 2.1 M solution in hexanes, 94.5 mmol, 3.15 eq.) was added dropwise. The solution was warmed to -10 °C for 10 minutes and the mixture was re-cooled to -50 °C and stirred vigorously at that temperature for 15 minutes. The solution of diethyl methylphosphonate (5.0 g, 33.0 mmol, 1.1 eq.) in 2-MeTHF (20 mL) was added dropwise over 10 minutes and the mixture was stirred for 30 min at -50°C. Next, the solution of corresponding methyl 2-(sulfonylamino)benzoate (30.0 mmol, 1.0 equiv.) in 2-MeTHF (20 mL) was added slowly to the orange suspension. The solution was allowed to warm to room temperature overnight and quenched by adding aqueous 1 M HCl (100 mL), extracted with $CHCl_3$ (2 × 100 mL). The organic layers were combined, washed with water, brine and dried over anhydrous $MgSO_4$. The evaporation of the solvent gave crude product, which was purified by column chromatography (eluent: chloroform/acetone 30:1).

Diethyl (2-oxo-2-(2-(phenylsulfonamido)phenyl)ethyl)phosphonate (**9a**)

Yield: 9.4 g, 76% as a white solid. R_f (chloroform/acetone 30:1) 0.28, mp 59-60°C.
^{31}P NMR (284 MHz, $CDCl_3$) δ 18.88.
^{1}H NMR (700 MHz, $CDCl_3$) δ 11.27 (s, 1H), 7.90 (dd, *J* = 8.0, 1.5 Hz, 1H), 7.85 (dq, *J* = 7.6, 1.0 Hz, 2H), 7.67 (dd, *J* = 8.4, 1.0 Hz, 1H), 7.50 (ddt, *J* = 7.8, 6.9, 1.2 Hz, 1H), 7.45 (ddd, *J* = 8.6, 7.3, 1.5 Hz, 1H), 7.45 – 7.40 (m, 2H), 7.07 (ddd, *J* = 8.0, 7.3, 1.1 Hz, 1H), 4.09 (dqd, *J* = 7.8, 7.1, 0.9 Hz, 4H), 3.54 (d, *J* = 22.7 Hz, 2H), 1.25 (t, *J* = 7.1 Hz, 4H).
^{13}C NMR (176 MHz, $CDCl_3$) δ 196.11 (d, *J* = 6.3 Hz), 140.43, 139.48, 135.58, 133.12, 132.95, 129.13, 127.25, 122.73, 121.97 (d, *J* = 2.0 Hz), 118.83, 62.84 (d, *J* = 6.5 Hz), 39.87 (d, *J* = 130.0 Hz), 16.26 (d, *J* = 6.3 Hz).
Anal.Calcd. for $C_{18}H_{22}NO_6PS$: C, 52.55; H, 5.39. Found: C, 52.73; H, 5.48.

Diethyl (2-(2-((4-chlorophenyl)sulfonamido)phenyl)-2-oxoethyl)phosphonate (**9b**)

Yield: 9.4 g, 70% as a white solid. R_f (chloroform/acetone 30:1) 0.30, mp 63-64°C.
^{31}P NMR (284 MHz, $CDCl_3$) δ 18.74.
^{1}H NMR (700 MHz, $CDCl_3$) δ 11.28 (s, 1H), 7.91 (dd, *J* = 8.1, 1.5 Hz, 1H), 7.79 – 7.75 (m, 2H), 7.66 (dd, *J* = 8.4, 1.1 Hz, 1H), 7.47 (ddd, *J* = 8.4, 7.3, 1.5 Hz, 1H), 7.40 – 7.36 (m, 2H), 7.10 (ddd, *J* = 8.0, 7.3, 1.1 Hz, 1H), 4.12 – 4.05 (m, 4H), 3.55 (d, *J* = 22.8 Hz, 2H), 1.24 (t, *J* = 7.1 Hz, 6H).
^{13}C NMR (176 MHz, $CDCl_3$) δ 196.27 (d, *J* = 6.3 Hz), 140.21, 139.68, 138.04, 135.71, 133.11, 129.48, 128.79, 123.06, 122.07 (d, *J* = 2.3 Hz), 118.93, 62.91 (d, *J* = 6.3 Hz), 39.93 (d, *J* = 129.5 Hz), 16.30 (d, *J* = 6.3 Hz).
Anal.Calcd. for $C_{18}H_{21}ClNO_6PS$: C, 48.49; H, 4.75. Found: C, 48.37; H, 4.87.

Diethyl (2-(4,5-dimethoxy-2-((4-methylphenyl)sulfonamido)phenyl)-2-oxoethyl)phosphonate (**9c**)

Yield: 11.7 g, 80% as a pale orange solid. R_f (chloroform/acetone 30:1) 0.25, mp 130-132°C.
^{31}P NMR (284 MHz, $CDCl_3$) δ 19.59.
^{1}H NMR (700 MHz, $CDCl_3$) δ 11.37 (s, 1H), 7.71 – 7.64 (m, 2H), 7.36 (s, 1H), 7.26 (s, 1H), 7.21 – 7.17 (m, 2H), 4.13 – 4.01 (m, 4H), 3.89 (s, 3H), 3.84 (s, 3H), 3.44 (d, *J* = 22.8 Hz, 2H), 2.34 (s, 3H), 1.24 (td, *J* = 7.0, 0.5 Hz, 6H).

^{13}C NMR (176 MHz, $CDCl_3$) δ 193.78 (d, *J* = 5.8 Hz), 155.06, 144.29, 144.01, 137.00, 136.58, 129.73, 127.35, 114.89 (d, *J* = 1.8 Hz), 114.50, 102.31, 62.90 (d, *J* = 6.3 Hz), 56.40, 56.35, 40.35 (d, *J* = 129.0 Hz), 21.59, 16.36 (d, *J* = 6.3 Hz).
Anal.Calcd. for $C_{21}H_{28}NO_8PS$: C, 51.95; H, 5.81. Found: C, 51.85; H, 5.84.

Diethyl (2-(5-chloro-2-((4-methylphenyl)sulfonamido)phenyl)-2-oxoethyl)phosphonate (**9d**)

Yield: 9.0 g, 65% as a white solid. R_f (chloroform/acetone 30:1) 0.35, mp 71-72°C.
^{31}P NMR (284 MHz, $CDCl_3$) δ 18.15.
^1H NMR (700 MHz, $CDCl_3$) δ 11.06 (s, 1H), 7.86 (d, *J* = 2.5 Hz, 1H), 7.72 – 7.69 (m, 2H), 7.64 (d, *J* = 9.0 Hz, 1H), 7.40 (dd, *J* = 9.0, 2.5 Hz, 1H), 7.24 – 7.21 (m, 2H), 4.17 – 4.06 (m, 4H), 3.51 (d, *J* = 22.8 Hz, 2H), 2.35 (s, 3H), 1.27 (t, *J* = 7.1 Hz, 6H).
^{13}C NMR (176 MHz, $CDCl_3$) δ 195.13 (d, *J* = 6.5 Hz), 144.33, 139.09, 136.29, 135.28, 132.37, 129.87, 127.87, 127.34, 122.93 (d, *J* = 2.0 Hz), 120.31, 62.99 (d, *J* = 6.5 Hz), 40.09 (d, *J* = 129.6 Hz), 21.58, 16.26 (d, *J* = 6.5 Hz).
Anal.Calcd. for $C_{19}H_{23}ClNO_6PS$: C, 49.62; H, 5.04. Found: C, 49.58; H, 5.14.

Diethyl (2-(5-bromo-2-((4-methylphenyl)sulfonamido)phenyl)-2-oxoethyl)phosphonate (**9e**)

Yield: 13.2 g, 87% as a light brown solid. R_f (chloroform/acetone 30:1) 0.37, mp 81-82°C.
^{31}P NMR (284 MHz, $CDCl_3$) δ 18.17.
^1H NMR (700 MHz, $CDCl_3$) δ 11.09 (s, 1H), 8.00 (d, *J* = 2.3 Hz, 1H), 7.74 – 7.70 (m, 2H), 7.58 (d, *J* = 9.0 Hz, 1H), 7.53 (dd, *J* = 9.0, 2.3 Hz, 1H), 7.27 – 7.21 (m, 2H), 4.18 – 4.05 (m, 4H), 3.52 (d, *J* = 22.8 Hz, 2H), 2.36 (s, 3H), 1.27 (t, *J* = 7.1 Hz, 6H).
^{13}C NMR (176 MHz, $CDCl_3$) δ 195.05 (d, *J* = 6.4 Hz), 144.35, 139.59, 138.14, 136.27, 135.32, 129.88, 127.34, 123.21 (d, *J* = 1.9 Hz), 120.47, 115.01, 63.01 (d, *J* = 6.4 Hz), 40.08 (d, *J* = 129.6 Hz), 21.59, 16.28 (d, *J* = 6.4 Hz).
Anal.Calcd. for $C_{19}H_{23}BrNO_6PS$: C, 45.25; H, 4.60. Found: C, 45.36; H, 4.47.

2.2. *Synthesis of diethyl (4-hydroxy-1-sulfonyl-1,2-dihydroquinolin-3-yl)phosphonate* (**10a-t**)

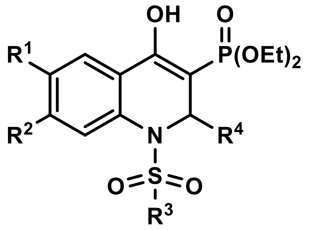

To a solution of corresponding diethyl (2-(2-(sulfonamido)phenyl)-2-oxoethyl)phosphonate (**7a-e**) (2.0 mmol, 1 eq.) and aldehyde (aryl: 2.4 mmol, 1.2 eq.; alkyl: 3.0 mmol, 1.5 eq.) in ethanol (10 mL) was added pyrrolidine acetate (58 mg, 20 mol%) at room temperature. The mixture was stirred for 24-48h. Upon completion of the reaction (monitored by ^{31}P-NMR), the solvent was removed in vacuum. The residue was dissolved in ethyl acetate (20 mL) and washed with water, brine and dried over anhydrous $MgSO_4$. The evaporation of the solvent gave crude product, which was purified by column chromatography (eluent: hexane/ethyl acetate 3:1).

Diethyl (2-ethyl-4-hydroxy-1-phenylsulfonyl-1,2-dihydroquinolin-3-yl)phosphonate (**10a**)

Yield: 420 mg, 46% as a pale yellow solid. R_f (hexane/ethyl acetate 3:1) 0.36, mp 116-118°C.
^{31}P NMR (284 MHz, $CDCl_3$) δ 21.67 (enol 94.0%), 19.21 (ketone *cis* 4.0%), 18.07 (ketone *trans* 2.0%).
^1H NMR (700 MHz, $CDCl_3$) δ 10.58 (d, *J* = 1.0 Hz, 1H), 7.77 (dd, *J* = 8.1, 1.1 Hz, 1H), 7.57 (dd, *J* = 7.7, 1.6 Hz, 1H), 7.45 – 7.41 (m, 4H), 7.27 – 7.22 (m, 3H), 4.81 (ddd, *J* = 11.2, 10.0, 3.5 Hz, 1H), 4.17 – 4.11 (m, 2H), 4.07 (dp, *J* = 10.2, 7.2 Hz, 1H), 3.94 (ddq, *J* = 10.2, 8.1, 7.2 Hz, 1H), 1.54 (dqd, *J* = 14.5, 7.2, 3.5 Hz, 1H), 1.46 (t, *J* = 7.2 Hz, 3H), 1.30 (t, *J* = 7.2 Hz, 3H), 1.30 – 1.24 (m, 1H), 0.96 (t, *J* = 7.2 Hz, 3H).
^{13}C NMR (176 MHz, $CDCl_3$) δ ^{13}C NMR (176 MHz, Chloroform-*d*) δ 160.29 (d, *J* = 6.9 Hz), 138.64, 135.26 (d, *J* = 1.8 Hz), 132.90, 131.19, 128.43, 127.29, 127.22, 126.54, 125.31 (d, *J* = 14.5 Hz), 124.28, 92.04 (d, *J* = 186.4 Hz), 62.53 (d, *J* = 6.5 Hz), 62.37 (d, *J* = 5.1 Hz), 56.02 (d, *J* = 12.2 Hz), 27.04, 16.48 (d, *J* = 6.7 Hz), 16.22 (d, *J* = 6.7 Hz), 10.47.
Anal.Calcd. for $C_{21}H_{26}NO_6PS$: C, 55.87; H, 5.80. Found: C, 56.09; H, 5.77.

Diethyl (4-hydroxy-2-isopropyl-1-phenylsulfonyl-1,2-dihydroquinolin-3-yl)phosphonate (**10b**)

Yield: 670 mg, 72% as a pale yellow solid. R_f (hexane/ethyl acetate 3:1) 0.38, mp 108-110°C.
^{31}P NMR (284 MHz, $CDCl_3$) δ 22.15 (enol 97.0%), 19.21 (ketone *cis* 1.0%), 18.54 (ketone *trans* 2.0%).
^1H NMR (700 MHz, $CDCl_3$) δ 10.78 (s, 1H), 7.75 (dd, *J* = 8.1, 1.2 Hz, 1H), 7.53 (dd, *J* = 7.6, 1.6 Hz, 1H), 7.43 (dt, *J* = 7.6, 1.2 Hz, 1H), 7.42 – 7.39 (m, 3H), 7.26 – 7.23 (m, 2H), 7.21 (td, *J* = 7.6, 1.2 Hz, 1H), 4.92 (dd, *J* = 10.6, 4.3 Hz, 1H), 4.19 – 4.14 (m, 2H), 4.08 (dq, *J* = 10.2, 7.1 Hz, 1H), 3.93 (ddq, *J* = 10.2, 8.1, 7.1 Hz, 1H), 1.87 (pd, *J* = 6.8, 4.2 Hz, 1H), 1.47 (td, *J* = 7.1, 0.5 Hz, 3H), 1.29 (td, *J* = 7.1, 0.5 Hz, 3H), 1.12 (d, *J* = 6.8 Hz, 3H), 0.47 (d, *J* = 6.8 Hz, 3H).

^{13}C NMR (176 MHz, $CDCl_3$) δ 161.17 (d, *J* = 6.4 Hz), 138.15, 137.72 (d, *J* = 1.8 Hz), 132.94, 131.29, 128.41, 127.34, 126.59, 126.32, 125.89 (d, *J* = 14.3 Hz), 124.07, 90.08 (d, *J* = 185.0 Hz), 62.61 (d, *J* = 6.8 Hz), 62.42 (d, *J* = 5.1 Hz), 59.06 (d, *J* = 12.6 Hz), 34.10, 19.46, 16.59, 16.50 (d, *J* = 6.4 Hz), 16.22 (d, *J* = 6.8 Hz).
Anal.Calcd. for $C_{22}H_{28}NO_6PS$: C, 56.77; H, 6.06. Found: C, 56.97; H, 6.10.

Diethyl (4-hydroxy-2-phenyl-1-phenylsulfonyl-1,2-dihydroquinolin-3-yl)phosphonate (**10c**)

Yield: 710 mg, 71% as a white solid. R_f (hexane/ethyl acetate 3:1) 0.38, mp 134-136°C.
^{31}P NMR (284 MHz, $CDCl_3$) δ 20.95 (enol 99.0%), 17.44 (ketone *trans* 1.0%).
^{1}H NMR (700 MHz, $CDCl_3$) δ 10.86 (s, 1H), 7.67 (dd, *J* = 7.6, 1.6 Hz, 1H), 7.58 (dd, *J* = 8.1, 1.2 Hz, 1H), 7.52 – 7.50 (m, 2H), 7.46 (tt, *J* = 7.4, 1.2 Hz, 1H), 7.35 (ddd, *J* = 8.1, 7.6, 1.6 Hz, 1H), 7.31 – 7.27 (m, 2H), 7.25 (td, *J* = 7.7, 1.2 Hz, 1H), 7.21 – 7.16 (m, 5H), 6.12 (d, *J* = 10.6 Hz, 1H), 4.16 (dqd, *J* = 7.9, 7.0, 0.7 Hz, 2H), 3.78 (dqd, *J* = 9.9, 7.0, 6.0 Hz, 1H), 3.31 (ddq, *J* = 9.9, 7.9, 7.0 Hz, 1H), 1.48 (td, *J* = 7.1, 0.7 Hz, 253H), 0.76 (td, *J* = 7.1, 0.7 Hz, 256H).
^{13}C NMR (176 MHz, $CDCl_3$) δ 161.35 (d, *J* = 6.3 Hz), 138.71, 138.27, 135.46, 135.44, 133.07, 131.46, 128.53, 128.45, 128.33, 127.77, 127.40, 127.35, 126.62, 125.57, 125.49, 124.15, 89.93 (d, *J* = 188.1 Hz), 62.62 (d, *J* = 6.5 Hz), 62.46 (d, *J* = 5.1 Hz), 57.11 (d, *J* = 13.0 Hz), 16.49 (d, *J* = 6.4 Hz), 15.48 (d, *J* = 7.5 Hz).
Anal.Calcd. for $C_{25}H_{26}NO_6PS$: C, 60.11; H, 5.25. Found: C, 60.12; H, 5.38.

Diethyl (4-hydroxy-2-(4-methoxyphenyl)-1-phenylsulfonyl-1,2-dihydroquinolin-3-yl)phosphonate (**10d**)

Yield: 920 mg, 87% as a white solid. R_f (hexane/ethyl acetate 3:1) 0.31, mp 142-144°C.
^{31}P NMR (284 MHz, $CDCl_3$) δ 21.06 (enol 99.0%), 17.43 (ketone *trans* 1.0%).
^{1}H NMR (700 MHz, $CDCl_3$) δ 10.84 (d, *J* = 1.1 Hz, 1H), 7.66 (dd, *J* = 7.7, 1.6 Hz, 1H), 7.59 – 7.56 (m, 1H), 7.52 – 7.49 (m, 2H), 7.46 (ddt, *J* = 8.7, 7.4, 1.2 Hz, 1H), 7.35 (ddd, *J* = 8.1, 7.4, 1.6 Hz, 1H), 7.31 – 7.27 (m, 2H), 7.24 (td, *J* = 7.6, 1.2 Hz, 1H), 7.13 – 7.09 (m, 2H), 6.72 – 6.69 (m, 2H), 6.07 (d, *J* = 10.6 Hz, 1H), 4.15 (dq, *J* = 8.0, 7.1 Hz, 2H), 3.80 (dqd, *J* = 10.0, 7.1, 6.2 Hz, 1H), 3.70 (s, 3H), 3.34 (ddq, *J* = 10.0, 8.0, 7.1 Hz, 1H), 1.48 (td, *J* = 7.0, 0.6 Hz, 3H), 0.82 (td, *J* = 7.0, 0.6 Hz, 3H).
^{13}C NMR (176 MHz, $CDCl_3$) δ 161.21 (d, *J* = 6.3 Hz), 159.61, 138.42, 135.47, 133.02, 131.42, 130.97, 129.05, 128.53, 127.41, 127.35, 126.56, 125.61, 125.53, 124.13, 113.77, 90.25 (d, *J* = 187.8 Hz), 62.60 (d, *J* = 6.6 Hz), 62.48 (d, *J* = 5.0 Hz), 56.62 (d, *J* = 13.0 Hz), 55.29, 29.77, 16.51 (d, *J* = 6.7 Hz), 15.64 (d, *J* = 7.0 Hz).
Anal.Calcd. for $C_{26}H_{28}NO_7PS$: C, 58.97; H, 5.33. Found: C, 58.81; H, 5.43.

Diethyl (1-(4-chlorophenyl)sulfonyl-2-ethyl-4-hydroxy-1,2-dihydroquinolin-3-yl)phosphonate (**10e**)

Yield: 780 mg, 80% as a white solid. R_f (hexane/ethyl acetate 3:1) 0.55, mp 108-110°C.
^{31}P NMR (284 MHz, $CDCl_3$) δ 21.50 (enol 96.0%), 18.94 (ketone *cis* 2.5%), 17.96 (ketone *trans* 1.5%).
^{1}H NMR (700 MHz, $CDCl_3$) δ 10.63 (s, 1H), 7.76 (dd, *J* = 8.2, 1.2 Hz, 1H), 7.60 (dd, *J* = 7.8, 1.6 Hz, 1H), 7.44 (td, *J* = 7.8, 1.6 Hz, 1H), 7.40 – 7.36 (m, 2H), 7.27 (td, *J* = 7.1, 6.5, 1.2 Hz, 1H), 7.24 – 7.20 (m, 2H), 4.81 (ddd, *J* = 11.2, 10.0, 3.6 Hz, 1H), 4.17 (p, *J* = 7.1 Hz, 2H), 4.10 (dp, *J* = 10.0, 7.1 Hz, 1H), 4.00 – 3.92 (m, 1H), 1.54 (dqd, *J* = 14.5, 7.3, 3.6 Hz, 1H), 1.47 (t, *J* = 7.1 Hz, 3H), 1.31 (t, *J* = 7.1 Hz, 3H), 1.30 – 1.26 (m, 1H), 0.96 (t, *J* = 7.3 Hz, 3H).
^{13}C NMR (176 MHz, $CDCl_3$) δ 160.35 (d, *J* = 6.5 Hz), 139.44, 137.23, 135.00, 131.33, 128.81, 128.71, 127.18, 126.76, 125.28 (d, *J* = 14.5 Hz), 124.52, 92.06 (d, *J* = 186.8 Hz), 62.61 (d, *J* = 6.5 Hz), 62.54 (d, *J* = 5.1 Hz), 56.18 (d, *J* = 12.6 Hz), 27.07, 16.51 (d, *J* = 6.4 Hz), 16.25 (d, *J* = 6.5 Hz), 10.48.
Anal.Calcd. for $C_{21}H_{25}ClNO_6PS$: C, 51.91; H, 5.19. Found: C, 51.78; H, 5.10.

Diethyl (1-(4-chlorophenyl)sulfonyl-4-hydroxy-2-isopropyl-1,2-dihydroquinolin-3-yl)phosphonate (**10f**)

Yield: 540 mg, 54% as a white solid. R_f (hexane/ethyl acetate 3:1) 0.54, mp 126-128°C.
^{31}P NMR (284 MHz, $CDCl_3$) δ 21.98 (enol 98.0%), 18.42 (ketone *trans* 2.0%).
^{1}H NMR (700 MHz, $CDCl_3$) δ 10.83 (s, 1H), 7.73 (dd, *J* = 8.2, 1.2 Hz, 1H), 7.55 (dd, *J* = 7.8, 1.6 Hz, 1H), 7.41 (td, *J* = 7.7, 1.6 Hz, 1H), 7.37 – 7.33 (m, 2H), 7.23 (dd, *J* = 7.6, 1.2 Hz, 1H), 7.22 – 7.19 (m, 2H), 4.91 (dd, *J* = 10.6, 4.4 Hz, 1H), 4.18 (dtd, *J* = 11.8, 7.2, 3.5 Hz, 2H), 4.11 (dp, *J* = 10.2, 7.2 Hz, 1H), 3.98 – 3.90 (m, 1H), 1.86 (pd, *J* = 6.9, 4.4 Hz, 1H), 1.47 (t, *J* = 7.1 Hz, 3H), 1.30 (t, *J* = 7.1 Hz, 3H), 1.10 (d, *J* = 6.9 Hz, 3H), 0.47 (d, *J* = 6.9 Hz, 3H).
^{13}C NMR (176 MHz, $CDCl_3$) δ 161.21 (d, *J* = 6.4 Hz), 139.46, 137.42 (d, *J* = 1.8 Hz), 136.77, 131.40, 128.82, 128.68, 126.52, 126.50, 125.85 (d, *J* = 14.5 Hz), 124.32, 90.12 (d, *J* = 185.2 Hz), 62.68 (d, *J* = 6.8 Hz), 62.57 (d, *J* = 5.0 Hz), 59.23 (d, *J* = 12.6 Hz), 34.09, 19.44, 16.62, 16.51 (d, *J* = 6.2 Hz), 16.23 (d, *J* = 6.4 Hz).
Anal.Calcd. for $C_{22}H_{27}ClNO_6PS$: C, 52.85; H, 5.44. Found: C, 52.64; H, 5.52.

Diethyl (1-(4-chlorophenyl)sulfonyl-4-hydroxy-2-phenyl-1,2-dihydroquinolin-3-yl)phosphonate (**10g**)

Yield: 780 mg, 73% as a white solid. R_f (hexane/ethyl acetate 3:1) 0.52, mp 150°C.
^{31}P NMR (284 MHz, $CDCl_3$) δ 20.81 (enol 99.0%), 17.42 (ketone *trans* 1.0%).

^{1}H NMR (700 MHz, $CDCl_3$) δ 10.88 (s, 1H), 7.70 (dd, *J* = 7.8, 1.6 Hz, 1H), 7.56 (dd, *J* = 8.1, 1.2 Hz, 1H), 7.48 – 7.43 (m, 2H), 7.36 (td, *J* = 7.8, 1.6 Hz, 1H), 7.27 (ddd, *J* = 8.8, 5.8, 1.2 Hz, 2H), 7.27 (d, *J* = 8.8 Hz, 1H), 7.22 – 7.17 (m, 5H), 6.11 (d, *J* = 10.7 Hz, 1H), 4.23 – 4.13 (m, 2H), 3.81 (dqd, *J* = 9.9, 7.0, 6.0 Hz, 1H), 3.32 (ddq, *J* = 9.9, 7.9, 7.0 Hz, 1H), 1.48 (t, *J* = 7.1 Hz, 3H), 0.77 (t, *J* = 7.1 Hz, 3H).
^{13}C NMR (176 MHz, $CDCl_3$) δ 161.43 (d, *J* = 6.3 Hz), 139.64, 138.52, 136.87, 135.20, 131.59, 128.92, 128.82, 128.51, 128.45, 127.80, 127.32, 126.84, 125.51 (d, *J* = 14.2 Hz), 124.38, 89.94 (d, *J* = 188.3 Hz), 62.72 (d, *J* = 6.8 Hz), 62.63 (d, *J* = 5.0 Hz), 57.24 (d, *J* = 13.3 Hz), 16.52 (d, *J* = 6.4 Hz), 15.50 (d, *J* = 7.3 Hz).
Anal.Calcd. for $C_{22}H_{25}ClNO_6PS$: C, 56.24; H, 4.72. Found: C, 56.16; H, 4.86.

Diethyl (1-(4-chlorophenyl)sulfonyl-4-hydroxy-2-(4-methoxyphenyl)-1,2-dihydroquinolin-3-yl)phosphonate (**10h**)

Yield: 870 mg, 77% as a white solid. R_f (hexane/ethyl acetate 3:1) 0.40, mp 136-138°C.
^{31}P NMR (284 MHz, $CDCl_3$) δ 20.94 (enol 99.0%), 17.33 (ketone *trans* 1.0%).
^{1}H NMR (700 MHz, $CDCl_3$) δ 10.88 (d, *J* = 1.0 Hz, 1H), 7.68 (dd, *J* = 7.8, 1.6 Hz, 1H), 7.55 (dd, *J* = 8.1, 1.2 Hz, 1H), 7.45 – 7.42 (m, 2H), 7.35 (ddd, *J* = 8.1, 7.4, 1.6 Hz, 1H), 7.26 (td, *J* = 7.4, 1.2 Hz, 1H), 7.27 – 7.24 (m, 1H), 7.12 – 7.08 (m, 2H), 6.72 – 6.68 (m, 2H), 6.06 (d, *J* = 10.6 Hz, 1H), 4.21 – 4.12 (m, 2H), 3.85 – 3.77 (m, 1H), 3.70 (s, 3H), 3.34 (ddq, *J* = 9.9, 8.1, 7.1 Hz, 1H), 1.47 (td, *J* = 7.1, 0.6 Hz, 3H), 0.82 (td, *J* = 7.1, 0.6 Hz, 3H).
^{13}C NMR (176 MHz, $CDCl_3$) δ ^{13}C NMR (176 MHz, Chloroform-*d*) δ 161.25 (d, *J* = 6.4 Hz), 159.69, 139.56, 136.95, 135.17, 131.53, 130.71, 129.06, 128.89, 128.80, 127.32, 126.78, 125.52 (d, *J* = 14.1 Hz), 124.33, 113.82, 90.21 (d, *J* = 187.9 Hz), 62.65 (d, *J* = 7.1 Hz), 62.59 (d, *J* = 5.3 Hz), 56.73 (d, *J* = 13.2 Hz), 55.30, 16.53 (d, *J* = 6.4 Hz), 15.65 (d, *J* = 7.2 Hz).
Anal.Calcd. for $C_{26}H_{27}ClNO_7PS$: C, 55.37; H, 4.83. Found: C, 55.53; H, 4.97.

Diethyl (2-ethyl-4-hydroxy-6,7-dimethoxy-1-tosyl-1,2-dihydroquinolin-3-yl)phosphonate (**10i**)

Yield: 680 mg, 65% as a pale yellow solid. R_f (hexane/ethyl acetate 3:1) 0.13, mp 140-142°C.
^{31}P NMR (284 MHz, $CDCl_3$) δ 22.35 (enol 78.0%), 19.96 (ketone *cis* 16.0%), 18.79 (ketone *trans* 6.0%).
^{1}H NMR (700 MHz, $CDCl_3$) δ 10.57 (s, 1H), 7.33 – 7.31 (m, 2H), 7.30 (s, 1H), 7.05 – 7.03 (m, 2H), 7.02 (s, 1H), 4.78 (ddd, *J* = 11.1, 10.0, 3.6 Hz, 1H), 4.14 – 4.09 (m, 2H), 4.08 – 4.03 (m, 1H), 3.95 (s, 3H), 3.94 – 3.89 (m, 1H), 3.85 (s, 3H), 2.29 (s, 3H), 1.50 (ddq, *J* = 11.1, 7.3, 3.6 Hz, 1H), 1.44 (t, *J* = 7.1 Hz, 3H), 1.29 (t, *J* = 7.1 Hz, 3H), 0.95 (t, *J* = 7.3 Hz, 3H).
^{13}C NMR (176 MHz, $CDCl_3$) δ 160.49 (d, *J* = 6.9 Hz), 151.14, 147.45, 143.61, 135.72, 129.00, 127.39, 117.96 (d, *J* = 14.9 Hz), 110.36, 109.07, 106.11, 89.47 (d, *J* = 188.5 Hz), 62.44 (d, *J* = 6.4 Hz), 62.25 (d, *J* = 5.1 Hz), 56.35, 56.10, 56.08 (d, *J* = 12.3 Hz), 26.84, 21.57, 16.45 (d, *J* = 6.6 Hz), 16.22 (d, *J* = 6.5 Hz), 10.38.
Anal.Calcd. for $C_{24}H_{32}NO_8PS$: C, 54.85; H, 6.14. Found: C, 54.90; H, 6.18.

Diethyl (4-hydroxy-2-isopropyl-6,7-dimethoxy-1-tosyl-1,2-dihydroquinolin-3-yl)phosphonate (**10j**)

Yield: 550 mg, 51% as a pale yellow solid. R_f (hexane/ethyl acetate 3:1) 0.21, mp 129-130°C.
^{31}P NMR (284 MHz, $CDCl_3$) δ 22.89 (enol 89.0%), 19.86 (ketone *cis* 3.0%), 19.23 (ketone *trans* 8.0%).
^{1}H NMR (700 MHz, $CDCl_3$) δ 10.81 (s, 1H), 7.32 – 7.28 (m, 2H), 7.28 (s, 1H), 7.05 – 7.01 (m, 2H), 6.98 (s, 1H), 4.87 (dd, *J* = 10.6, 4.2 Hz, 1H), 4.13 (dq, *J* = 8.0, 7.1 Hz, 2H), 4.06 (dp, *J* = 10.2, 7.1 Hz, 1H), 3.94 (s, 3H), 3.93 – 3.88 (m, 1H), 3.84 (s, 3H), 1.83 (pd, *J* = 6.9, 4.2 Hz, 1H), 1.44 (t, *J* = 7.1 Hz, 3H), 1.28 (t, *J* = 7.1 Hz, 3H), 1.09 (d, *J* = 6.9 Hz, 3H), 0.48 (d, *J* = 6.9 Hz, 3H).
^{13}C NMR (176 MHz, $CDCl_3$) δ 161.35 (d, *J* = 6.5 Hz), 151.18, 147.25, 143.61, 135.26, 131.91, 128.97, 127.42, 118.57 (d, *J* = 15.1 Hz), 109.73, 105.83, 87.57 (d, *J* = 186.4 Hz), 62.47 (d, *J* = 6.5 Hz), 62.23 (d, *J* = 5.1 Hz), 59.04 (d, *J* = 13.0 Hz), 56.19 (d, *J* = 57.1 Hz), 33.95, 21.56, 19.36, 16.54, 16.45 (d, *J* = 6.4 Hz), 16.20 (d, *J* = 6.4 Hz).
Anal.Calcd. for $C_{25}H_{34}NO_8PS$: C, 55.65; H, 6.35. Found: C, 55.57; H, 6.49.

Diethyl (4-hydroxy-6,7-dimethoxy-2-phenyl-1-tosyl-1,2-dihydroquinolin-3-yl)phosphonate (**10k**)

Yield: 410 mg, 36% as a white solid. R_f (hexane/ethyl acetate 3:1) 0.20, mp 190°C.
^{31}P NMR (284 MHz, $CDCl_3$) δ 21.68 (enol 95.0%), 18.49 (ketone *cis* 2.0%), 18.09 (ketone *trans* 3.0%).
^{1}H NMR (700 MHz, $CDCl_3$) δ 10.87 (d, *J* = 1.1 Hz, 1H), 7.42 – 7.38 (m, 2H), 7.23 – 7.17 (m, 5H), 7.12 (s, 1H), 7.11 (s, 1H), 7.10 – 7.07 (m, 2H), 6.06 (d, *J* = 10.6 Hz, 1H), 4.17 – 4.08 (m, 2H), 3.88 (s, 3H), 3.84 (s, 3H), 3.76 (dqd, *J* = 9.9, 7.0, 6.0 Hz, 1H), 3.31 – 3.23 (m, 1H), 2.33 (s, 3H), 1.46 (t, *J* = 7.0 Hz, 3H), 0.74 (t, *J* = 7.0 Hz, 3H).
^{13}C NMR (176 MHz, $CDCl_3$) δ 161.56 (d, *J* = 6.8 Hz), 151.22, 147.47, 143.79, 139.10, 135.31, 129.70, 129.08, 128.40, 128.20, 127.82, 127.53, 118.30 (d, *J* = 14.6 Hz), 110.65, 105.86, 87.40 (d, *J* = 189.6 Hz), 62.47 (d, *J* = 6.5 Hz), 62.30 (d, *J* = 5.0 Hz), 57.14 (d, *J* = 13.4 Hz), 56.21, 56.07, 21.61, 16.47 (d, *J* = 6.4 Hz), 15.47 (d, *J* = 7.5 Hz).
Anal.Calcd. for $C_{28}H_{32}NO_8PS$: C, 58.63; H, 5.62. Found: C, 58.85; H, 5.69.

Diethyl (4-hydroxy-6,7-dimethoxy-2-(4-methoxyphenyl)-1-tosyl-1,2-dihydroquinolin-3-yl)phosphonate (**10l**)

Yield: 690 mg, 57% as a white solid. R_f (hexane/ethyl acetate 3:1) 0.15, mp 148-150°C.
^{31}P NMR (284 MHz, $CDCl_3$) δ 21.78 (enol 96.0%), 18.66 (ketone *cis* 2.0%), 18.13 (ketone *trans* 2.0%).

^{1}H NMR (700 MHz, $CDCl_3$) δ 10.85 (d, *J* = 1.1 Hz, 1H), 7.41 – 7.37 (m, 2H), 7.14 – 7.09 (m, 2H), 7.12 (s, 1H), 7.10 (s, 1H), 7.09 – 7.06 (m, 2H), 6.73 – 6.69 (m, 2H), 6.01 (d, *J* = 10.6 Hz, 1H), 4.12 (p, *J* = 7.2 Hz, 2H), 3.87 (s, 3H), 3.84 (s, 3H), 3.79 – 3.75 (m, 1H), 3.70 (s, 3H), 3.31 (ddt, *J* = 15.0, 9.9, 7.1 Hz, 1H), 2.32 (s, 3H), 1.45 (t, *J* = 7.0 Hz, 3H), 0.80 (t, *J* = 7.0 Hz 3H).
^{13}C NMR (176 MHz, $CDCl_3$) δ 161.38 (d, *J* = 6.6 Hz), 159.52, 151.19, 147.43, 143.73, 135.38, 131.33, 129.67, 129.06, 127.50, 118.29 (d, *J* = 14.6 Hz), 113.70, 110.64, 105.82, 87.66 (d, *J* = 189.7 Hz), 62.43 (d, *J* = 6.8 Hz), 62.31 (d, *J* = 5.0 Hz), 56.65, 56.57, 56.21, 56.07, 55.26, 21.60, 16.47 (d, *J* = 6.5 Hz), 15.61 (d, *J* = 7.1 Hz).
Anal.Calcd. for $C_{29}H_{34}NO_9PS$: C, 57.70; H, 5.68. Found: C, 57.72; H, 5.65.

Diethyl (6-chloro-2-ethyl-4-hydroxy-1-tosyl-1,2-dihydroquinolin-3-yl)phosphonate (**10m**)

Yield: 880 mg, 88% as a white solid. R_f (hexane/ethyl acetate 3:1) 0.41, mp 134-136°C.
^{31}P NMR (284 MHz, $CDCl_3$) δ 21.22 (enol 97.0%), 18.75 (ketone *cis* 2.0%), 17.62 (ketone *trans* 1.0%).
^{1}H NMR (700 MHz, $CDCl_3$) δ 10.62 (s, 1H), 7.72 (d, *J* = 8.6 Hz, 1H), 7.56 (d, *J* = 2.5 Hz, 1H), 7.37 (dd, *J* = 8.6, 2.5 Hz, 1H), 7.36 – 7.33 (m, 2H), 7.10 – 7.06 (m, 2H), 4.82 (ddd, *J* = 11.2, 10.2, 3.6 Hz, 1H), 4.19 – 4.10 (m, 2H), 4.11 – 4.05 (m, 1H), 3.96 (m, 1H), 2.32 (s, 3H), 1.55 (dqd, *J* = 14.4, 7.3, 3.6 Hz, 1H), 1.46 (td, *J* = 7.0, 0.5 Hz, 3H), 1.32 (td, *J* = 7.0, 0.5 Hz, 3H), 1.27 (dtd, *J* = 14.4, 7.3, 3.6 Hz, 1H), 0.94 (t, *J* = 7.3 Hz, 3H).
^{13}C NMR (176 MHz, $CDCl_3$) δ 159.12 (d, *J* = 6.9 Hz), 143.99, 135.68, 133.93 (d, *J* = 1.9 Hz), 132.21 (d, *J* = 2.3 Hz), 131.02, 129.24, 128.38, 127.36, 126.54 (d, *J* = 14.7 Hz), 124.35, 93.30 (d, *J* = 186.1 Hz), 62.69 (d, *J* = 6.6 Hz), 62.58 (d, *J* = 5.2 Hz), 56.04 (d, *J* = 12.1 Hz), 27.21, 21.62, 16.49 (d, *J* = 6.4 Hz), 16.26 (d, *J* = 6.6 Hz), 10.46.
Anal.Calcd. for $C_{22}H_{27}ClNO_6PS$: C, 52.85; H, 5.44. Found: C, 52.64; H, 5.32.

Diethyl (6-chloro-4-hydroxy-2-isopropyl-1-tosyl-1,2-dihydroquinolin-3-yl)phosphonate (**10n**)

Yield: 870 mg, 85% as a white solid. R_f (hexane/ethyl acetate 3:1) 0.48, mp 96-98°C.
^{31}P NMR (284 MHz, $CDCl_3$) δ 21.68 (enol 98.0%), 18.72 (ketone *cis* 0.5%), 18.09 (ketone *trans* 1.5%).
^{1}H NMR (700 MHz, $CDCl_3$) δ 10.81 (s, 1H), 7.69 (d, *J* = 8.6 Hz, 1H), 7.51 (d, *J* = 2.5 Hz, 1H), 7.34 (dd, *J* = 8.6, 2.5 Hz, 1H), 7.33 – 7.30 (m, 2H), 7.09 – 7.05 (m, 2H), 4.92 (dd, *J* = 10.6, 4.2 Hz, 1H), 4.17 (dq, *J* = 8.0, 7.1 Hz, 2H), 4.14 – 4.06 (m, 1H), 3.95 (ddq, *J* = 10.2, 8.0, 7.1 Hz, 1H), 2.31 (s, 3H), 1.86 (heptd, *J* = 7.0, 4.2 Hz, 1H), 1.47 (t, *J* = 7.1 Hz, 3H), 1.30 (t, *J* = 7.1 Hz, 3H), 1.10 (d, *J* = 7.0 Hz, 3H), 0.47 (d, *J* = 7.0 Hz, 3H).
^{13}C NMR (176 MHz, $CDCl_3$) δ 159.92 (d, *J* = 6.6 Hz), 144.02, 136.40 (d, *J* = 1.8 Hz), 135.17, 131.94 (d, *J* = 2.3 Hz), 131.07, 129.21, 127.74, 127.38, 127.10 (d, *J* = 15.1 Hz), 124.12, 91.31 (d, *J* = 184.4 Hz), 62.75 (d, *J* = 6.8 Hz), 62.61 (d, *J* = 5.1 Hz), 59.12 (d, *J* = 12.2 Hz), 34.23, 21.62, 19.44, 16.64, 16.49 (d, *J* = 6.4 Hz), 16.24 (d, *J* = 6.5 Hz).
Anal.Calcd. for $C_{23}H_{29}ClNO_6PS$: C, 53.75; H, 5.69. Found: C, 53.68; H, 5.61.

Diethyl (6-chloro-4-hydroxy-2-phenyl-1-tosyl-1,2-dihydroquinolin-3-yl)phosphonate (**10o**)

Yield: 820 mg, 75% as a pale yellow solid. R_f (hexane/ethyl acetate 3:1) 0.49, mp 158=160°C.
^{31}P NMR (284 MHz, $CDCl_3$) δ 20.48 (enol 99.0%), 16.94 (ketone *trans* 1.0%).
^{1}H NMR (700 MHz, $CDCl_3$) δ 10.90 (d, *J* = 1.1 Hz, 1H), 7.65 (d, *J* = 2.5 Hz, 1H), 7.52 (d, *J* = 8.7 Hz, 1H), 7.44 – 7.40 (m, 2H), 7.29 (dd, *J* = 8.7, 2.5 Hz, 1H), 7.23 – 7.17 (m, 5H), 7.13 – 7.10 (m, 2H), 6.11 (d, *J* = 10.6 Hz, 1H), 4.16 (dq, *J* = 7.9, 7.0 Hz, 2H), 3.79 (dqd, *J* = 9.9, 7.0, 6.1 Hz, 1H), 3.32 (ddq, *J* = 9.9, 7.9, 7.0 Hz, 1H), 2.35 (s, 3H), 1.48 (t, *J* = 7.0 Hz, 3H), 0.77 (t, *J* = 7.0 Hz, 3H).
^{13}C NMR (176 MHz, $CDCl_3$) δ 160.18 (d, *J* = 6.8 Hz), 144.20, 138.54 (d, *J* = 1.7 Hz), 135.36, 134.11 (d, *J* = 1.9 Hz), 132.29 (d, *J* = 2.3 Hz), 131.28, 129.37, 128.58, 128.55, 128.51, 127.75, 127.48, 126.74 (d, *J* = 14.4 Hz), 124.22, 91.19 (d, *J* = 187.4 Hz), 62.76 (d, *J* = 6.6 Hz), 62.66 (d, *J* = 5.1 Hz), 57.13 (d, *J* = 12.9 Hz), 21.68, 16.52 (d, *J* = 6.5 Hz), 15.53 (d, *J* = 7.4 Hz).
Anal.Calcd. for $C_{26}H_{27}ClNO_6PS$: C, 56.99; H, 4.97. Found: C, 56.94; H, 5.00.

Diethyl (6-chloro-4-hydroxy-2-(4-methoxyphenyl)-1-tosyl-1,2-dihydroquinolin-3-yl)phosphonate (**10p**)

Yield: 730 mg, 63% as a white solid. R_f (hexane/ethyl acetate 3:1) 0.33, mp 138-140°C.
^{31}P NMR (284 MHz, $CDCl_3$) δ 20.58 (enol 99.0%), 16.98 (ketone *trans* 1.0%).
^{1}H NMR (700 MHz, $CDCl_3$) δ 10.88 (d, *J* = 1.0 Hz, 1H), 7.64 (d, *J* = 2.6 Hz, 1H), 7.52 (d, *J* = 8.6 Hz, 1H), 7.42 – 7.39 (m, 2H), 7.29 (dd, *J* = 8.6, 2.6 Hz, 1H), 7.13 – 7.10 (m, 2H), 7.13 – 7.10 (m, 2H), 7.12 – 7.08 (m, 2H), 6.74 – 6.71 (m, 2H), 6.07 (d, *J* = 10.6 Hz, 1H), 4.15 (dq, *J* = 8.0, 7.0 Hz, 2H), 3.83 – 3.77 (m, 1H), 3.72 (s, 3H), 3.35 (ddq, *J* = 10.0, 8.0, 7.0 Hz, 1H), 2.34 (s, 3H), 1.47 (td, *J* = 7.1, 0.6 Hz, 3H), 0.82 (td, *J* = 7.1, 0.6 Hz, 3H).
^{13}C NMR (176 MHz, $CDCl_3$) δ 159.99 (d, *J* = 6.4 Hz), 159.71, 144.11, 135.41, 134.06 (d, *J* = 1.8 Hz), 132.19 (d, *J* = 2.2 Hz), 131.21, 130.71 (d, *J* = 1.7 Hz), 129.33, 129.00, 128.52, 127.43, 126.72 (d, *J* = 14.6 Hz), 124.15, 113.86, 91.41 (d, *J* = 187.1 Hz), 62.71 (d, *J* = 6.5 Hz), 62.66 (d, *J* = 5.1 Hz), 56.59 (d, *J* = 12.8 Hz), 55.32, 21.65, 16.50 (d, *J* = 6.4 Hz), 15.65 (d, *J* = 7.0 Hz).
Anal.Calcd. for $C_{27}H_{29}ClNO_7PS$: C, 56.11; H, 5.06. Found: C, 55.95; H, 5.20.

Diethyl (6-bromo-2-ethyl-4-hydroxy-1-tosyl-1,2-dihydroquinolin-3-yl)phosphonate (**10q**)

Yield: 650 mg, 60% as a white solid. R_f (hexane/ethyl acetate 3:1) 0.40, mp 138-140°C.
^{31}P NMR (284 MHz, $CDCl_3$) δ 21.15 (enol 96.0%), 18.71 (ketone *cis* 3.0%), 17.61 (ketone *trans* 1.0%).
^{1}H NMR (700 MHz, $CDCl_3$) δ 10.61 (s, 1H), 7.71 (d, *J* = 2.4 Hz, 1H), 7.65 (d, *J* = 8.6 Hz, 1H), 7.52 (dd, *J* = 8.6, 2.4 Hz, 1H), 7.37 – 7.32 (m, 2H), 7.11 – 7.05 (m, 2H), 4.81 (td, *J* = 10.7, 3.4 Hz, 1H), 4.15 (dq, *J* = 15.9, 7.9, 7.3 Hz, 1H), 4.09 (dt, *J* = 10.2, 7.3 Hz, 1H), 3.96 (dt, *J* = 10.1, 7.3 Hz, 1H), 2.32 (s, 3H), 1.55 (dqd, *J* = 14.6, 7.2, 3.4 Hz, 1H), 1.46 (t, *J* = 7.3 Hz, 3H), 1.31 (t, *J* = 7.3 Hz, 3H), 1.29 – 1.22 (m, 1H), 0.94 (t, *J* = 7.2 Hz, 3H).
^{13}C NMR (176 MHz, $CDCl_3$) δ 159.01 (d, *J* = 6.8 Hz), 144.00, 135.68, 134.47, 133.96, 129.26, 128.59, 127.34, 127.28, 126.76 (d, *J* = 14.7 Hz), 119.92 (d, *J* = 2.3 Hz), 93.31 (d, *J* = 185.9 Hz), 62.69 (d, *J* = 6.4 Hz), 62.58 (d, *J* = 5.1 Hz), 56.00 (d, *J* = 12.4 Hz), 27.23, 21.62, 16.48 (d, *J* = 6.4 Hz), 16.26 (d, *J* = 6.6 Hz), 10.46.
Anal.Calcd. for $C_{22}H_{27}BrNO_6PS$: C, 48.54; H, 5.00. Found: C, 48.69; H, 5.04.

Diethyl (6-bromo-4-hydroxy-2-isopropyl-1-tosyl-1,2-dihydroquinolin-3-yl)phosphonate (**10r**)

Yield: 860 mg, 77% as a pale yellow solid. R_f (hexane/ethyl acetate 3:1) 0.42, mp 126-128°C.
^{31}P NMR (284 MHz, $CDCl_3$) δ 21.62 (enol 98.0%), 18.68 (ketone *cis* 0.5%), 18.07 (ketone *trans* 1.5%).
^{1}H NMR (700 MHz, $CDCl_3$) δ 10.81 (s, 1H), 7.67 (d, *J* = 2.4 Hz, 1H), 7.63 (d, *J* = 8.6 Hz, 1H), 7.49 (dd, *J* = 8.6, 2.4 Hz, 1H), 7.34 – 7.30 (m, 2H), 7.09 – 7.05 (m, 2H), 4.92 (dd, *J* = 10.4, 4.2 Hz, 1H), 4.17 (dq, *J* = 8.0, 7.1 Hz, 2H), 4.13 – 4.06 (m, 1H), 3.95 (ddq, *J* = 10.4, 8.0, 7.1 Hz, 1H), 2.32 (s, 3H), 1.86 (pd, *J* = 6.9, 4.2 Hz, 1H), 1.47 (td, *J* = 7.1, 0.5 Hz, 3H), 1.30 (td, *J* = 7.1, 0.5 Hz, 3H), 1.10 (d, *J* = 6.9 Hz, 3H), 0.48 (d, *J* = 6.9 Hz, 3H).
^{13}C NMR (176 MHz, $CDCl_3$) δ 159.82 (d, *J* = 6.7 Hz), 144.03, 136.93 (d, *J* = 1.8 Hz), 135.18, 134.02, 129.23, 127.96, 127.37, 127.30, 127.07, 119.66 (d, *J* = 1.5 Hz), 91.33 (d, *J* = 184.4 Hz), 62.75 (d, *J* = 6.5 Hz), 62.62 (d, *J* = 5.4 Hz), 59.10 (d, *J* = 12.2 Hz), 34.25, 21.63, 19.43, 16.68, 16.49 (d, *J* = 6.4 Hz), 16.24 (d, *J* = 6.4 Hz).
Anal.Calcd. for $C_{23}H_{29}BrNO_6PS$: C, 49.47; H, 5.23. Found: C, 49.38; H, 5.34.

Diethyl (6-bromo-4-hydroxy-2-phenyl-1-tosyl-1,2-dihydroquinolin-3-yl)phosphonate (**10s**)

Yield: 900 mg, 76% as a white solid. R_f (hexane/ethyl acetate 3:1) 0.43, mp 170-172°C.
^{31}P NMR (284 MHz, $CDCl_3$) δ 20.43 (enol 99.0%), 16.92 (ketone *trans* 1.0%).
^{1}H NMR (700 MHz, $CDCl_3$) δ 10.90 (d, *J* = 1.0 Hz, 1H), 7.80 (d, *J* = 2.2 Hz, 1H), 7.46 (d, *J* = 8.6 Hz, 1H), 7.44 (dd, *J* = 8.6, 2.2 Hz, 1H), 7.43 – 7.41 (m, 2H), 7.23 – 7.17 (m, 5H), 7.14 – 7.10 (m, 2H), 6.11 (d, *J* = 10.6 Hz, 1H), 4.15 (dq, *J* = 7.9, 7.0 Hz, 2H), 3.79 (dqd, *J* = 9.9, 7.0, 6.1 Hz, 1H), 3.32 (ddq, *J* = 9.9, 7.9, 7.0 Hz, 1H), 2.35 (s, 3H), 1.47 (td, *J* = 7.0, 0.5 Hz, 3H), 0.77 (td, *J* = 7.0, 0.5 Hz, 3H).
^{13}C NMR (176 MHz, $CDCl_3$) δ 160.04 (d, *J* = 6.5 Hz), 144.19, 138.53, 135.35, 134.63 (d, *J* = 1.8 Hz), 134.19, 129.36, 128.72, 128.56, 128.50, 127.71, 127.44, 127.13, 126.98, 126.90, 120.01, 120.00, 91.18 (d, *J* = 187.1 Hz), 62.74 (d, *J* = 6.4 Hz), 62.64 (d, *J* = 5.1 Hz), 57.07 (d, *J* = 13.2 Hz), 21.66, 16.50 (d, *J* = 6.8 Hz), 15.51 (d, *J* = 7.1 Hz).
Anal.Calcd. for $C_{26}H_{27}BrNO_6PS$: C, 52.71; H, 4.59. Found: C, 52.49; H, 4.68.

Diethyl (6-bromo-4-hydroxy-2-(4-methoxyphenyl)-1-tosyl-1,2-dihydroquinolin-3-yl)phosphonate (**10t**)

Yield: 910 mg, 73% as a pale yellow solid. R_f (hexane/ethyl acetate 3:1) 0.37, mp 146-148°C.
^{31}P NMR (284 MHz, $CDCl_3$) δ 20.53 (enol 99.0%), 16.96 (ketone *trans* 1.0%).
^{1}H NMR (700 MHz, $CDCl_3$) δ 10.88 (d, *J* = 0.9 Hz, 1H), 7.79 (d, *J* = 2.2 Hz, 1H), 7.46 (d, *J* = 8.6 Hz, 1H), 7.43 (dd, *J* = 8.6, 2.2 Hz, 1H), 7.43 – 7.39 (m, 2H), 7.14 – 7.08 (m, 4H), 6.75 – 6.70 (m, 2H), 6.07 (d, *J* = 10.6 Hz, 1H), 4.14 (dqd, *J* = 8.0, 7.0, 0.7 Hz, 2H), 3.84 – 3.76 (m, 1H), 3.72 (s, 3H), 3.35 (ddq, *J* = 10.0, 8.0, 7.0 Hz, 1H), 2.35 (s, 3H), 1.47 (t, *J* = 7.1 Hz, 3H), 0.82 (td, *J* = 7.1, 0.7 Hz, 3H).
^{13}C NMR (176 MHz, $CDCl_3$) δ 159.87 (d, *J* = 6.4 Hz), 159.71, 144.13, 135.43, 134.60 (d, *J* = 1.7 Hz), 134.14, 130.72, 129.35, 128.99, 128.71, 127.41, 127.09, 126.98, 126.90, 119.94, 119.92, 113.86, 91.42 (d, *J* = 187.0 Hz), 62.71 (d, *J* = 6.5 Hz), 62.66 (d, *J* = 5.1 Hz), 56.55 (d, *J* = 12.9 Hz), 55.32, 21.65, 16.50 (d, *J* = 6.6 Hz), 15.65 (d, *J* = 7.1 Hz).
Anal.Calcd. for $C_{27}H_{29}BrNO_7PS$: C, 52.10; H, 4.70. Found: C, 51.97; H, 4.61.

3. Copies of NMR spectra of 3-methylidene-1-sulfonyl-2,3-dihydroquinolin-4(1*H*)-ones (5a-t)

2-Ethyl-3-methylidene-1-(phenylsulfonyl)-2,3-dihydroquinolin-4(1H)-one (**5a**)

^{1}H-NMR (700 MHz, $CDCl_3$)

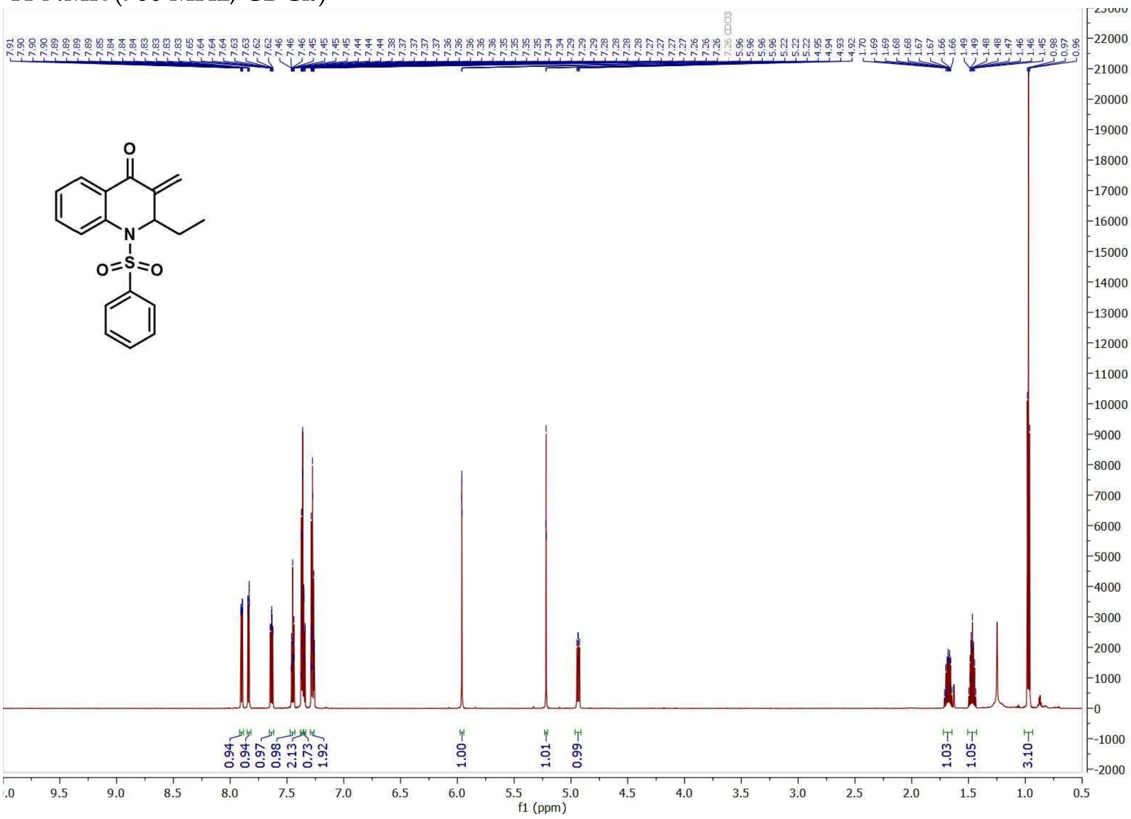

^{13}C NMR (176 MHz, $CDCl_3$)

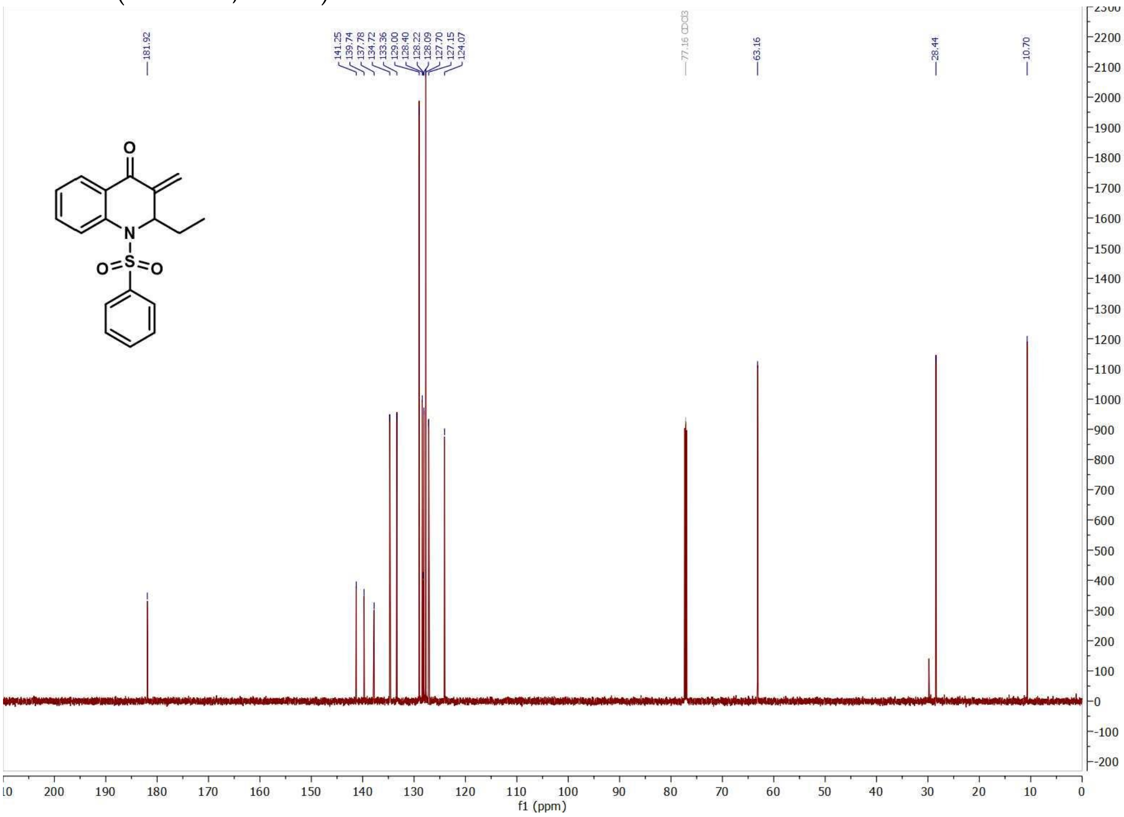

3-Methylidene-1-(phenylsulfonyl)-2-(propan-2-yl)-2,3-dihydroquinolin-4(1H)-one (**5b**)

^{1}H-NMR (700 MHz, $CDCl_3$)

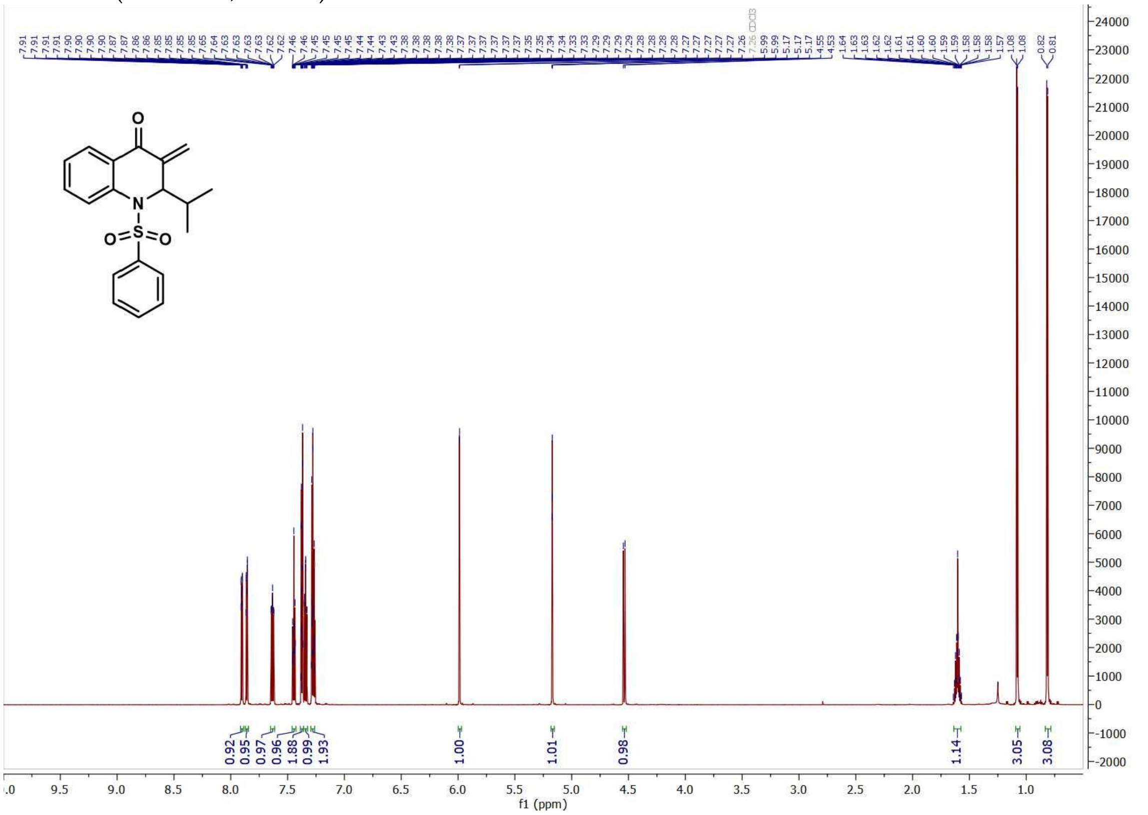

^{13}C NMR (176 MHz, $CDCl_3$)

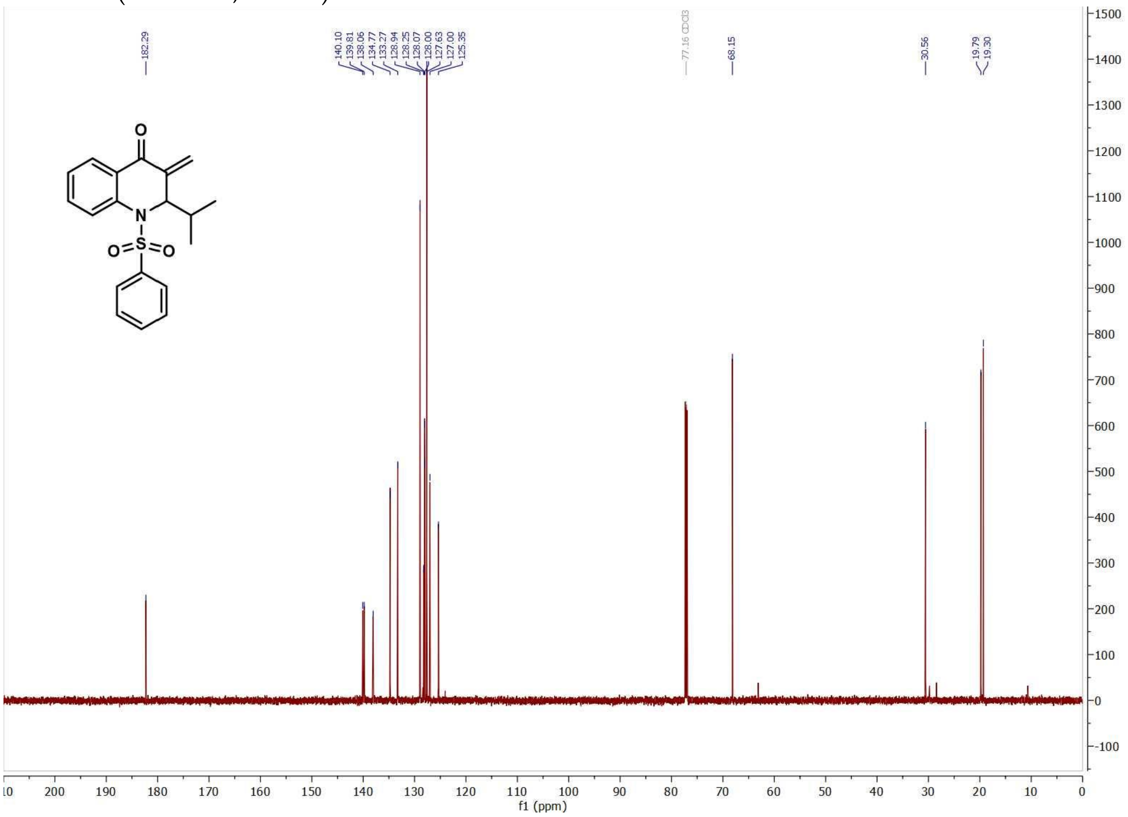

3-Methylidene-2-phenyl-1-(phenylsulfonyl)-2,3-dihydroquinolin-4(1H)-one (**5c**)

^{1}H-NMR (700 MHz, $CDCl_3$)

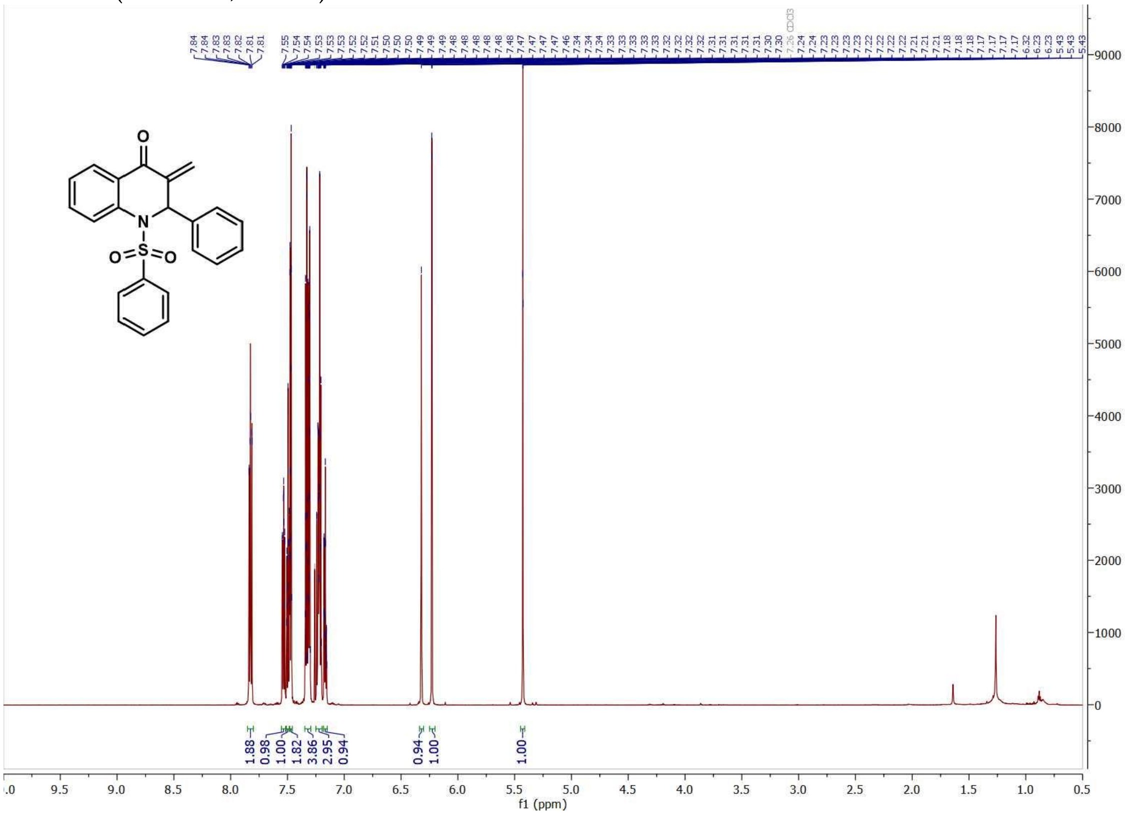

^{13}C NMR (176 MHz, $CDCl_3$)

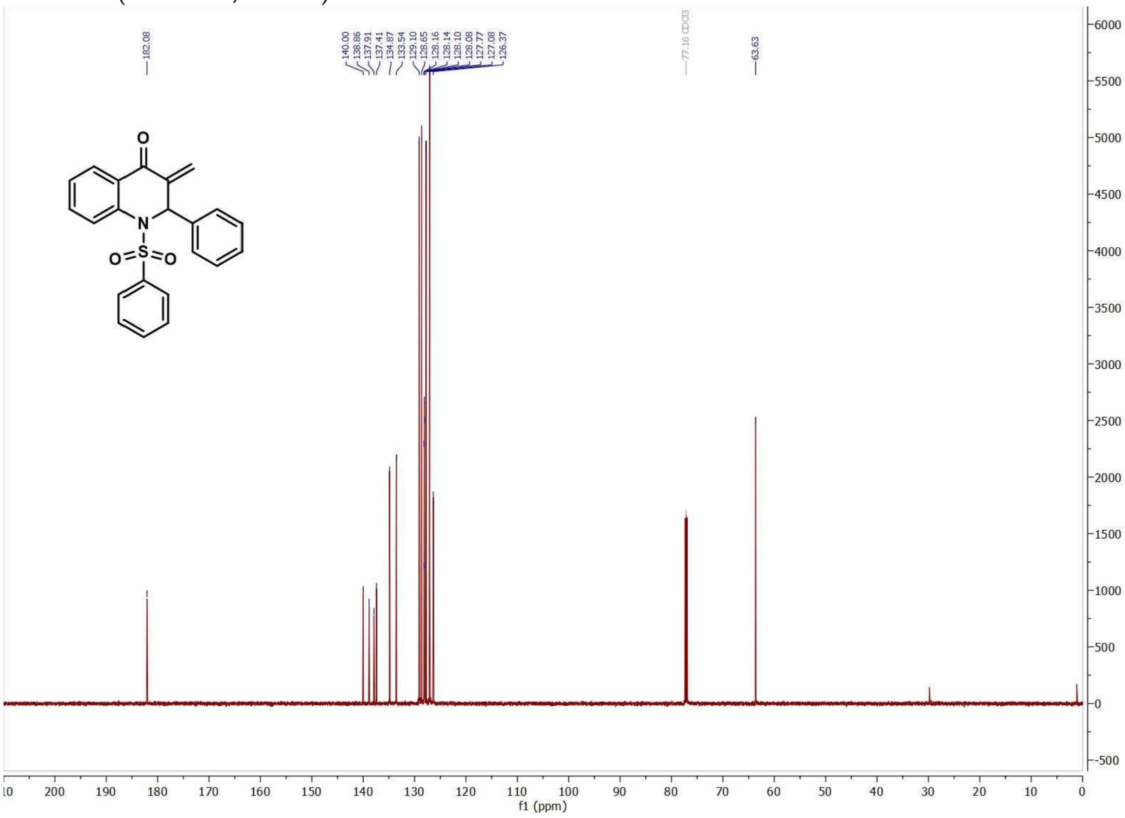

2-(4-Methoxyphenyl)-3-methylidene-1-(phenylsulfonyl)-2,3-dihydroquinolin-4(1H)-one (**5d**)

^{1}H-NMR (700 MHz, $CDCl_3$)

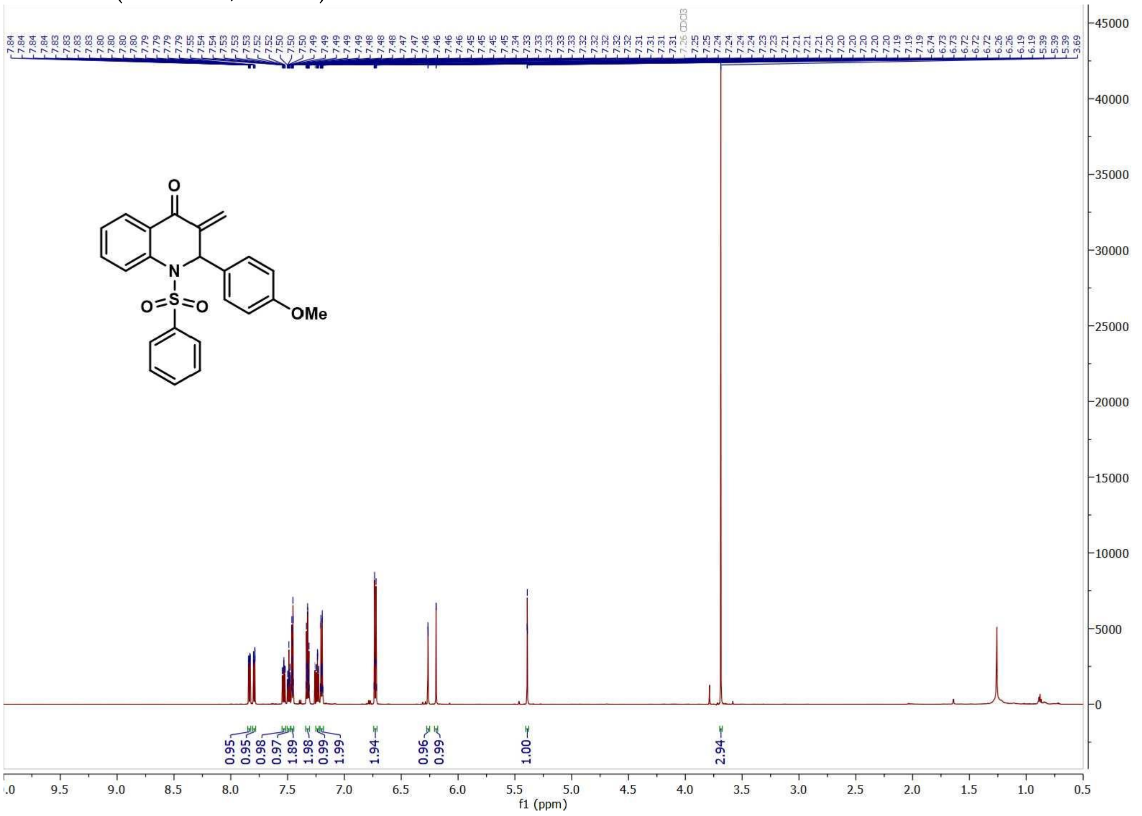

^{13}C NMR (176 MHz, $CDCl_3$)

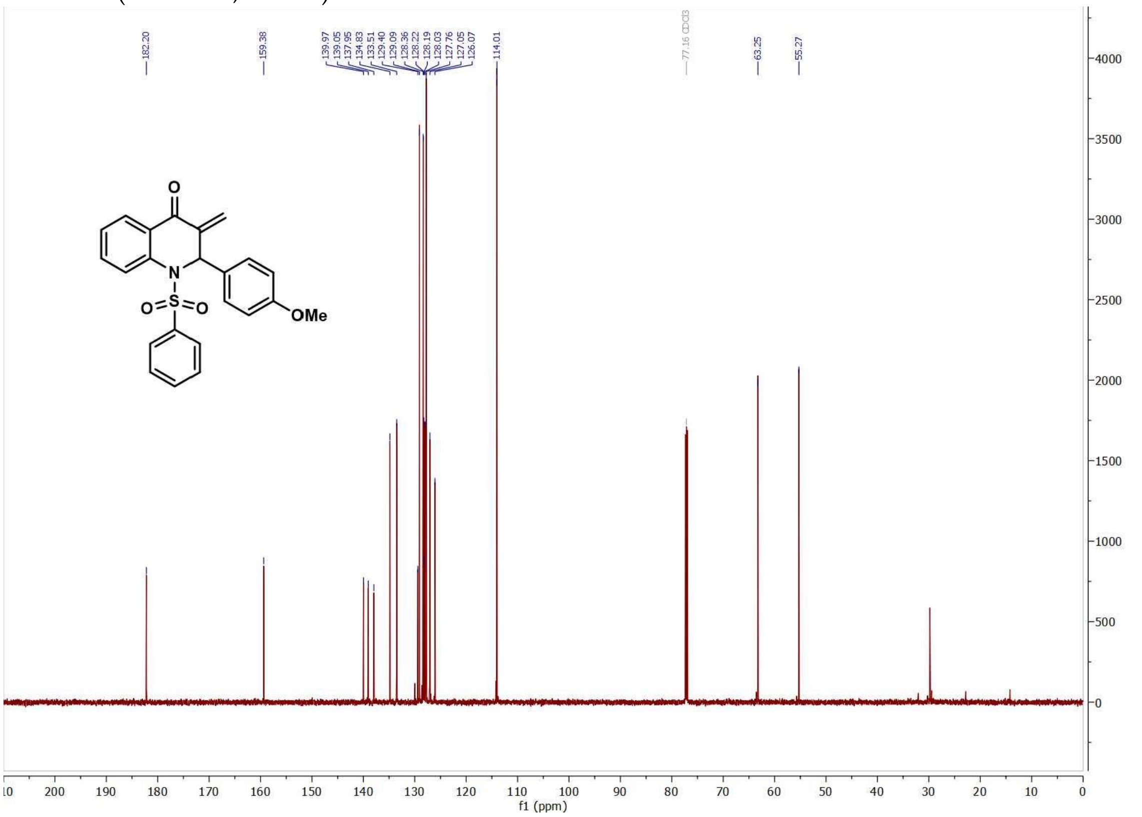

1-((4-Chlorophenyl)sulfonyl)-2-ethyl-3-methylidene-2,3-dihydroquinolin-4(1H)-one (**5e**)

^{1}H-NMR (700 MHz, CDCl$_3$)

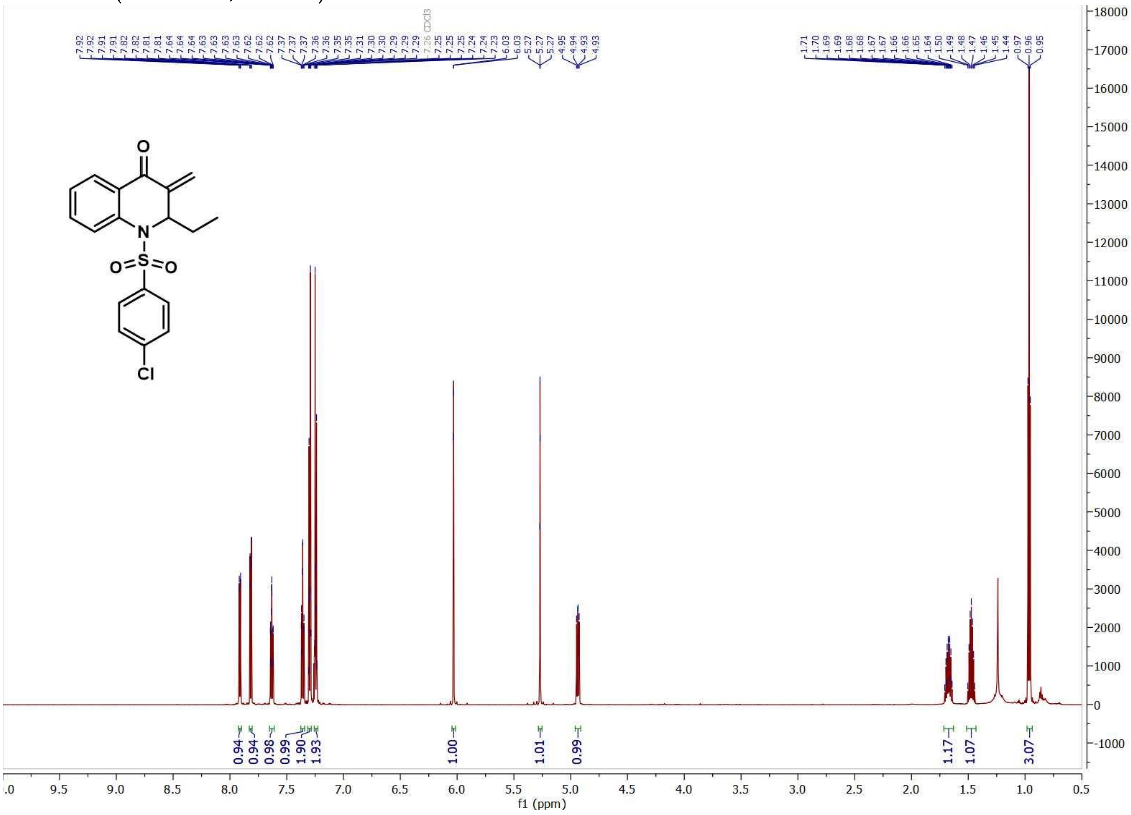

^{13}C NMR (176 MHz, CDCl$_3$)

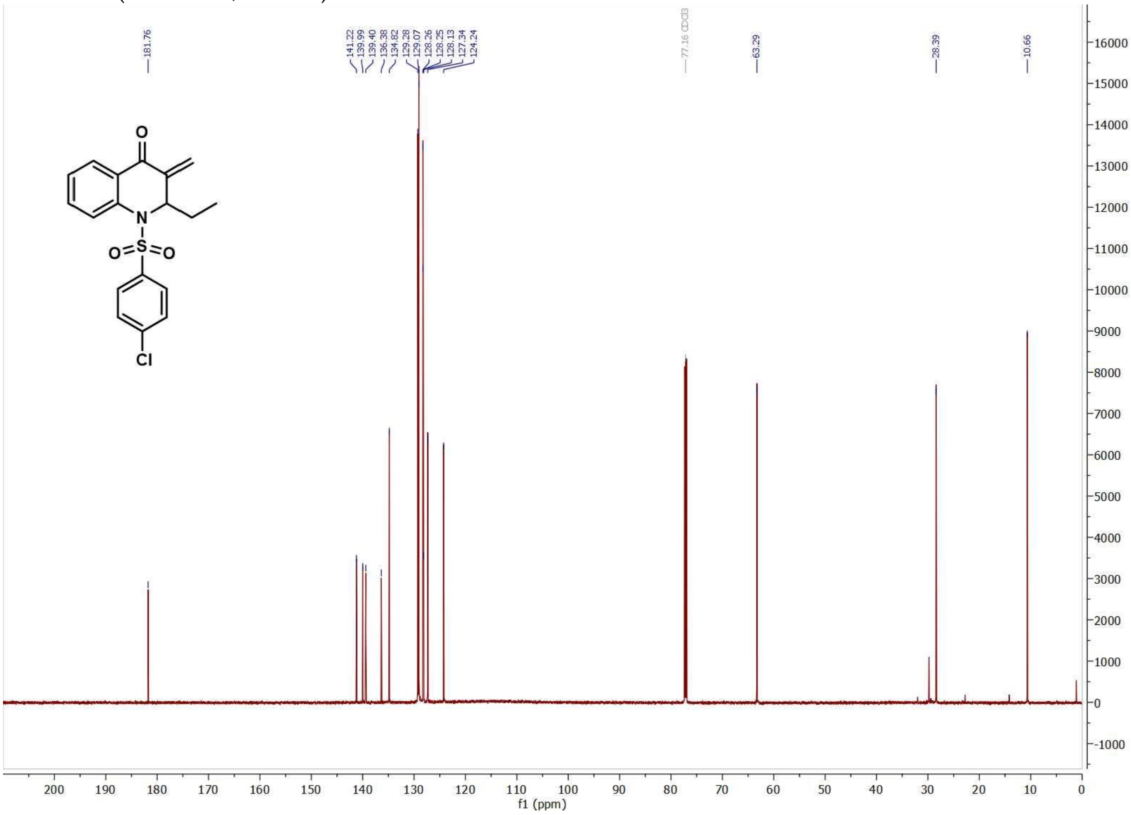

1-((4-Chlorophenyl)sulfonyl)-3-methylidene-2-(propan-2-yl)-2,3-dihydroquinolin-4(1H)-one (**5f**)

^{1}H-NMR (700 MHz, $CDCl_3$)

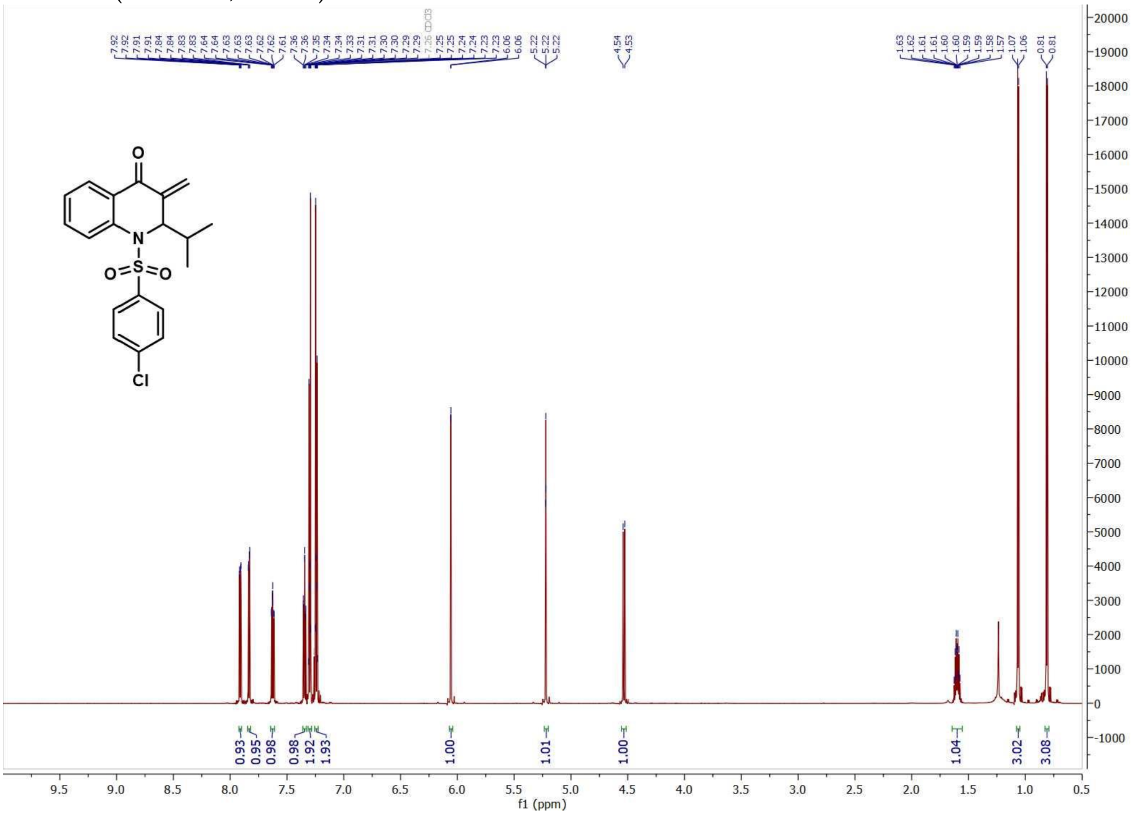

^{13}C NMR (176 MHz, $CDCl_3$)

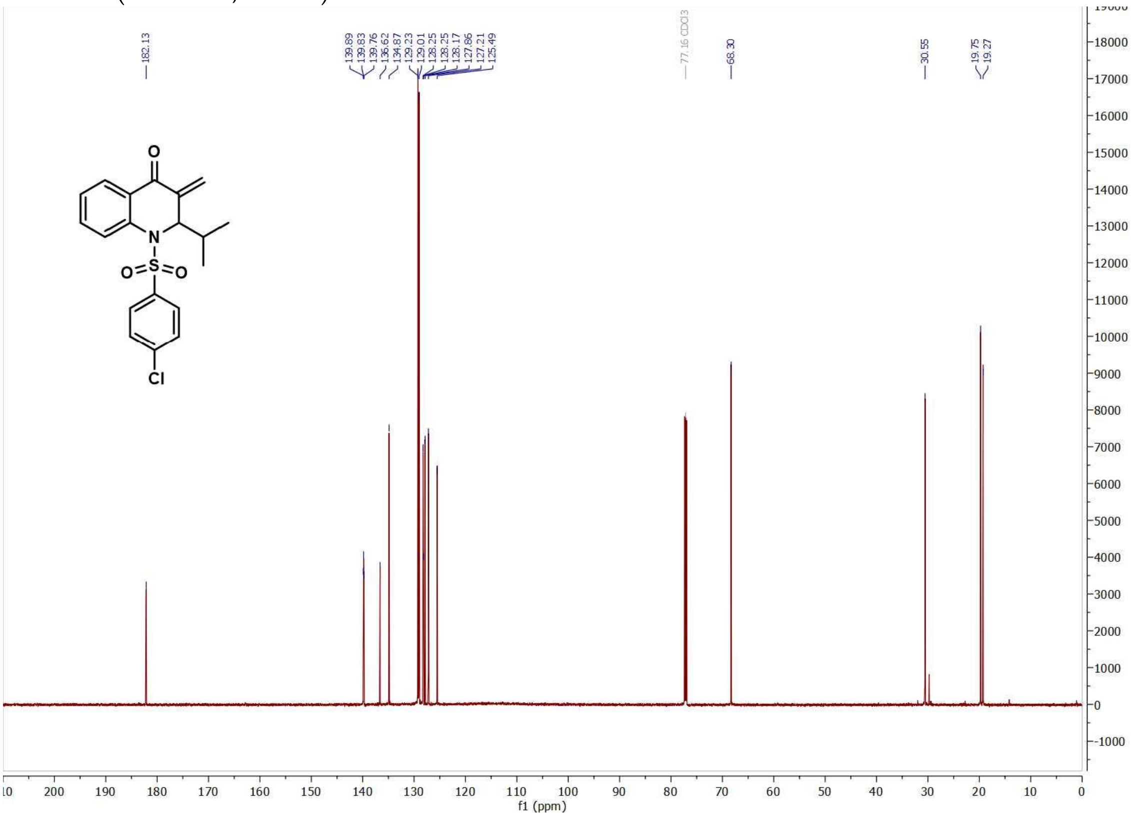

1-((4-Chlorophenyl)sulfonyl)-3-methylidene-2-phenyl-1-(phenylsulfonyl)-2,3-dihydroquinolin-4(1H)-one (**5g**)

^{1}H-NMR (700 MHz, $CDCl_3$)

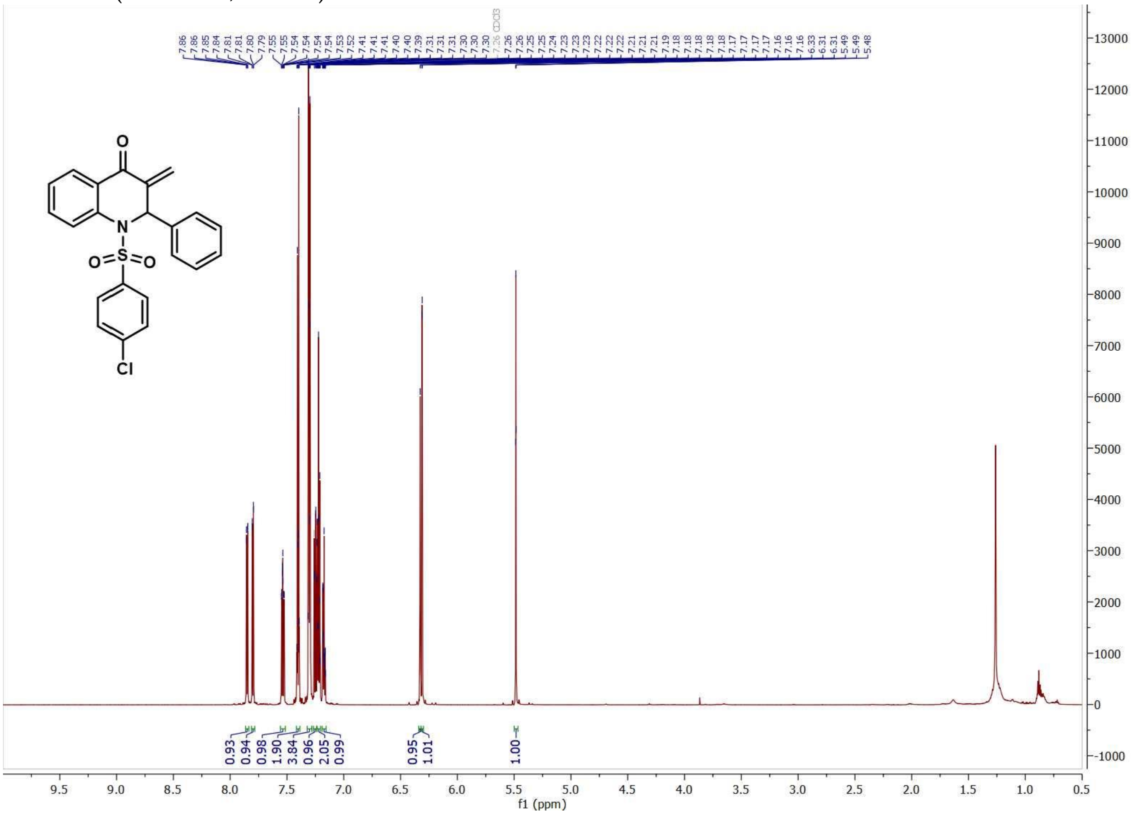

^{13}C NMR (176 MHz, $CDCl_3$)

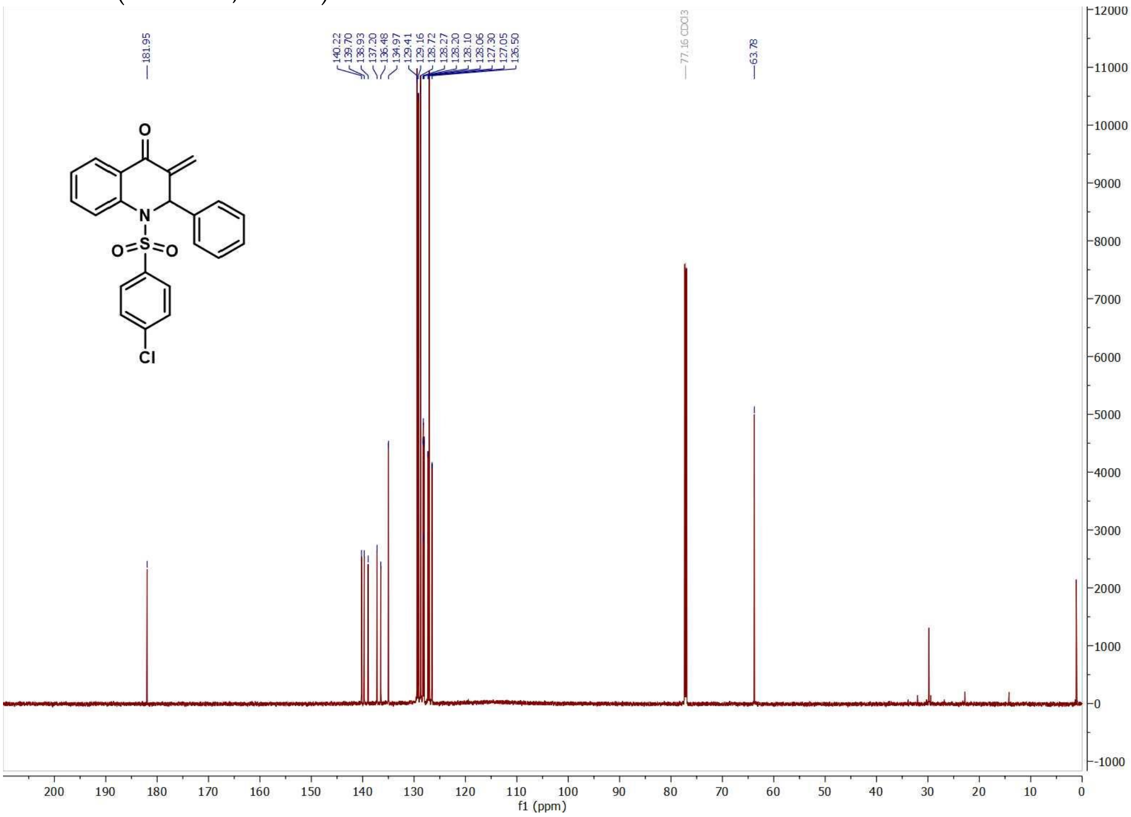

1-((4-Chlorophenyl)sulfonyl)-2-(4-methoxyphenyl)-3-methylidene-2,3-dihydroquinolin-4(1H)-one (**5h**)

^{1}H-NMR (700 MHz, $CDCl_3$)

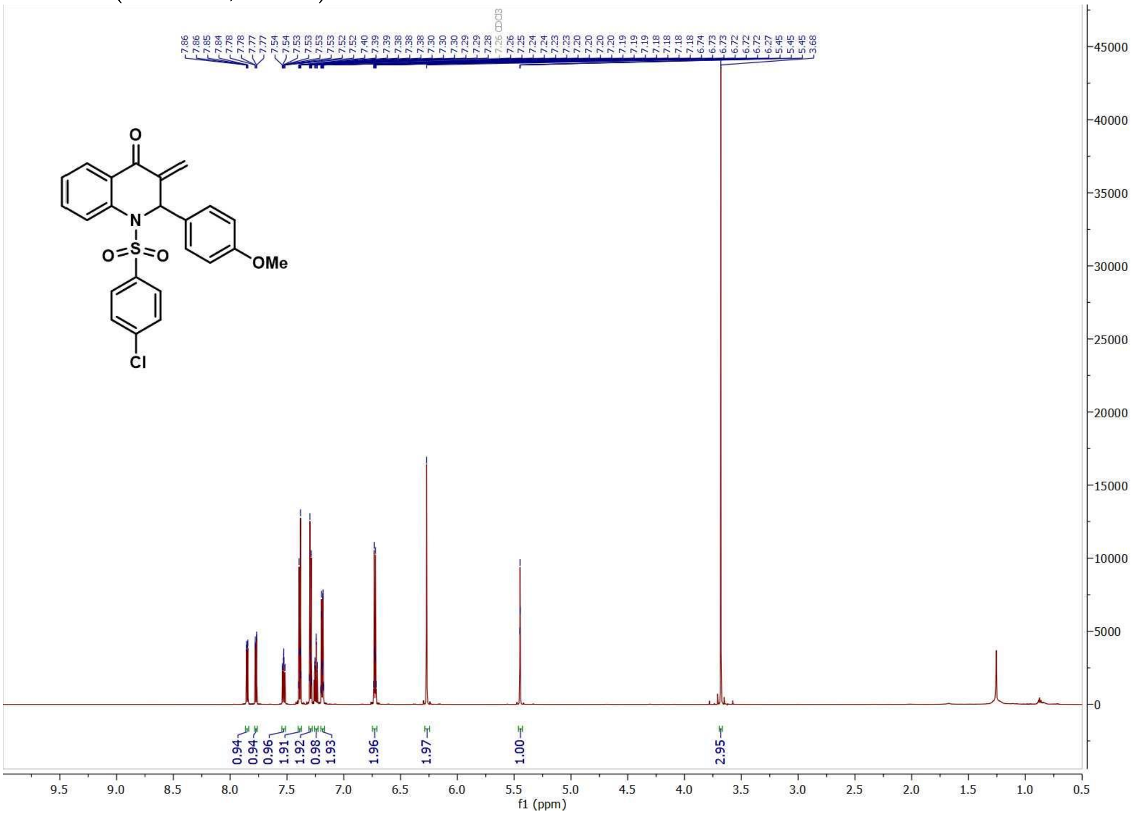

^{13}C NMR (176 MHz, $CDCl_3$)

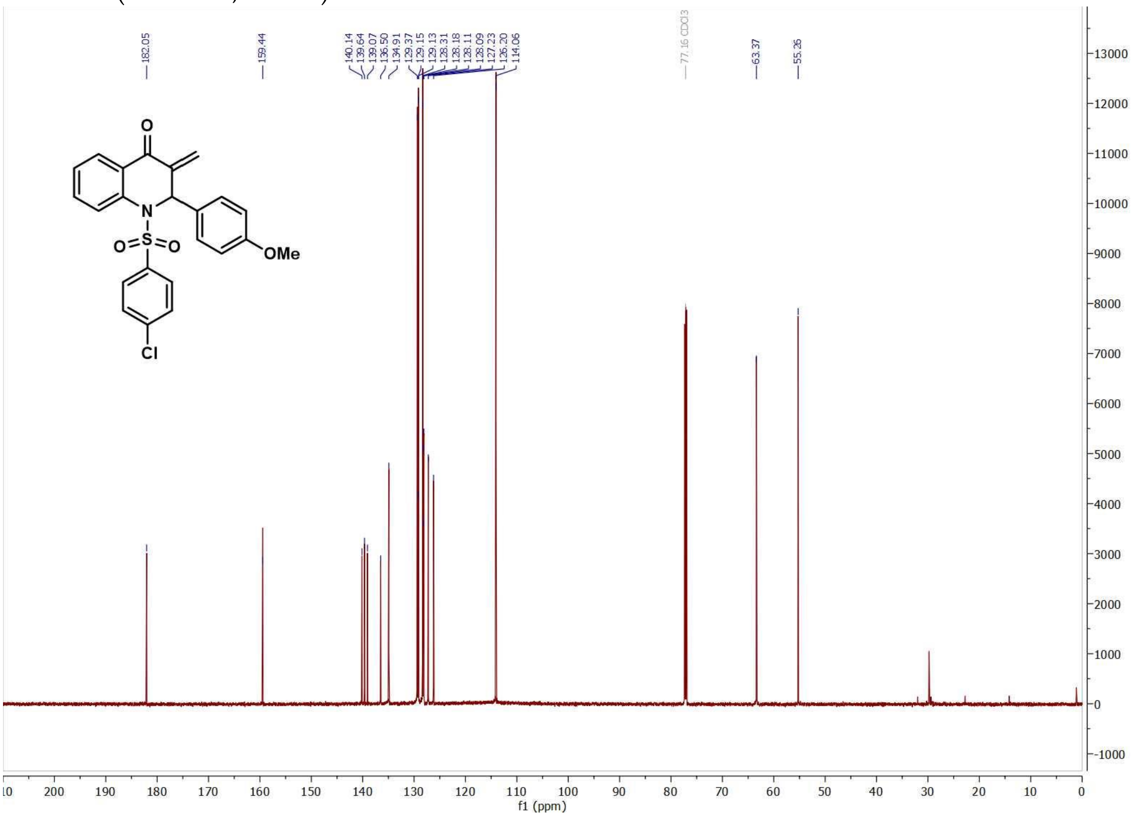

2-Ethyl-6,7-dimethoxy-3-methylidene-1-tosyl-2,3-dihydroquinolin-4(1H)-one (**5i**)

^{1}H-NMR (700 MHz, CDCl$_3$)

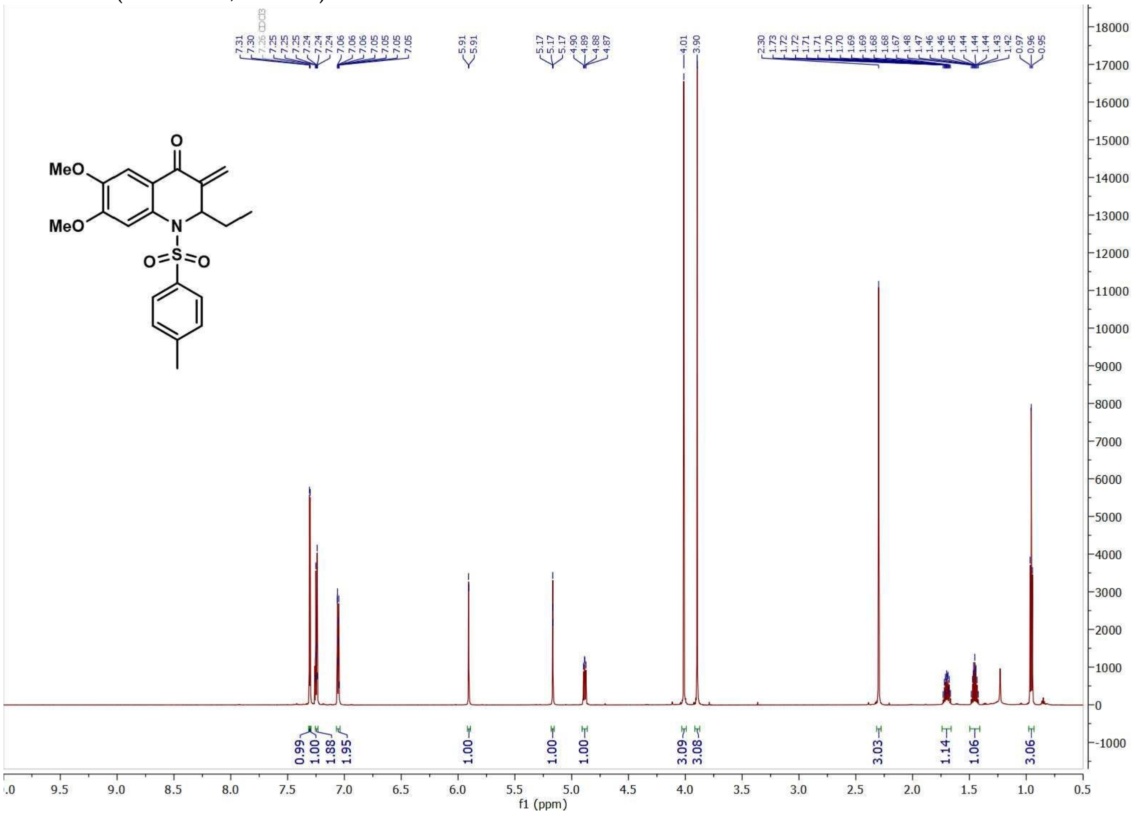

^{13}C NMR (176 MHz, CDCl$_3$)

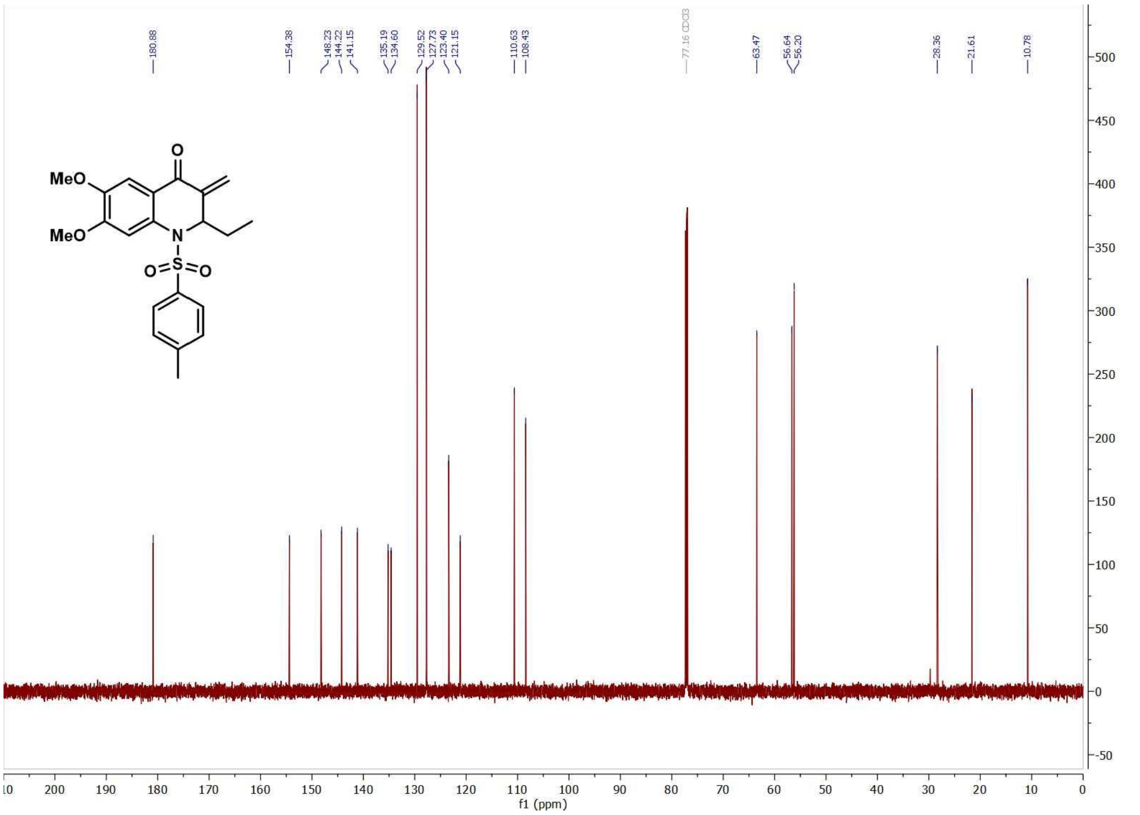

6,7-Dimethoxy-3-methylidene-2-(propan-2-yl)-1-tosyl-2,3-dihydroquinolin-4(1H)-one (**5j**)

^{1}H-NMR (700 MHz, $CDCl_3$)

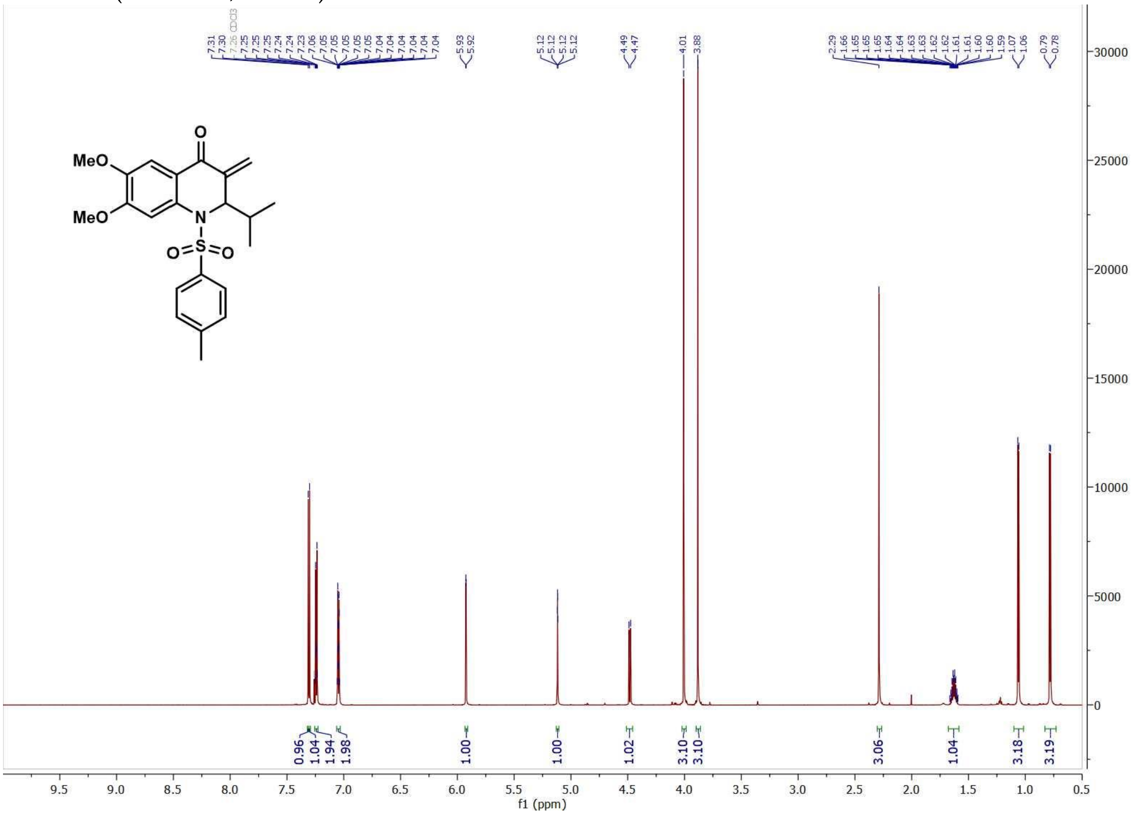

^{13}C NMR (176 MHz, $CDCl_3$)

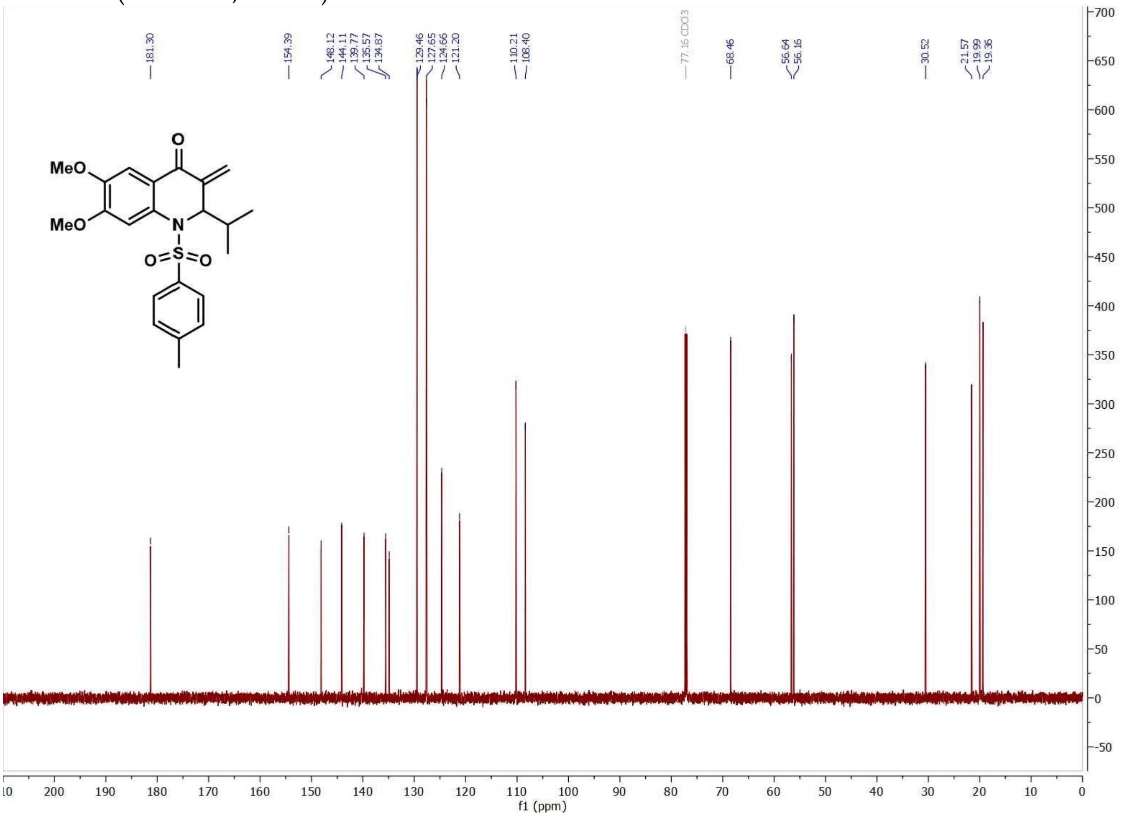

6,7-Dimethoxy-3-methylidene-2-phenyl-1-tosyl-2,3-dihydroquinolin-4(1H)-one (**5k**)

^{1}H-NMR (700 MHz, $CDCl_3$)

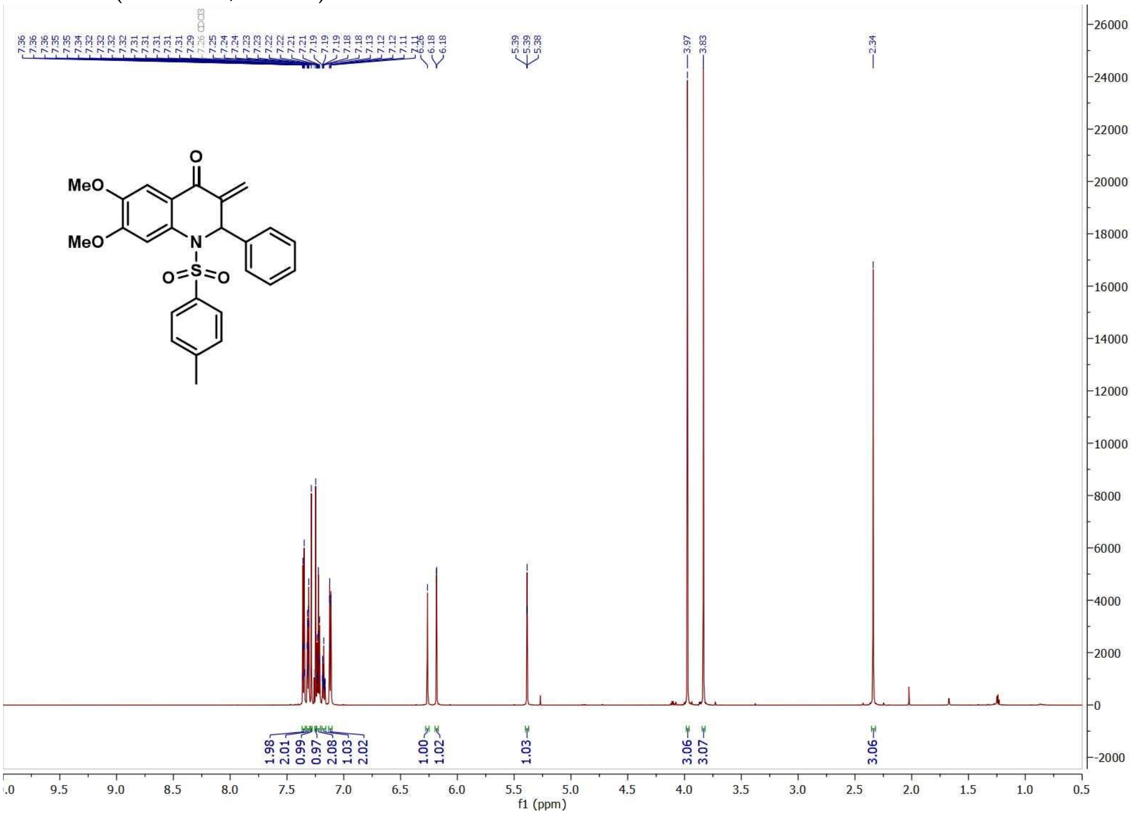

^{13}C NMR (176 MHz, $CDCl_3$)

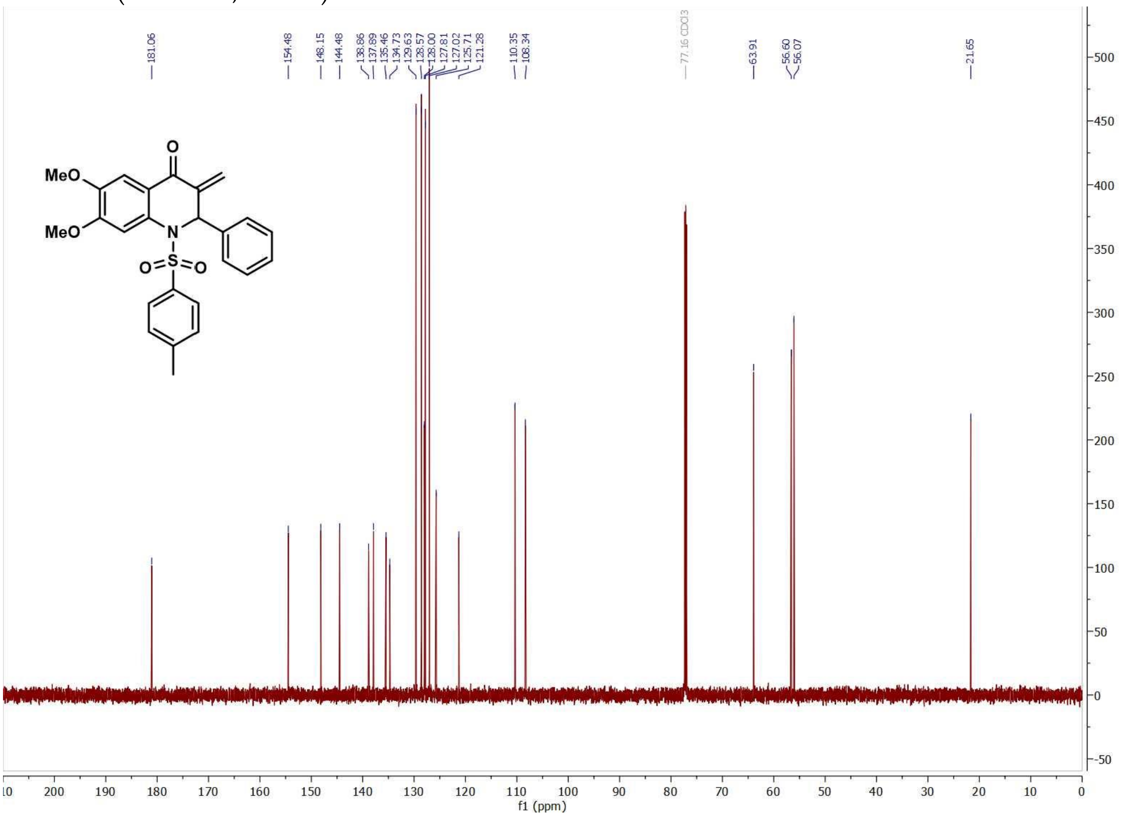

6,7-Dimethoxy-2-(4-methoxyphenyl)-3-methylidene-1-tosyl-2,3-dihydroquinolin-4(1H)-one (**5l**)

^{1}H-NMR (700 MHz, CDCl$_3$)

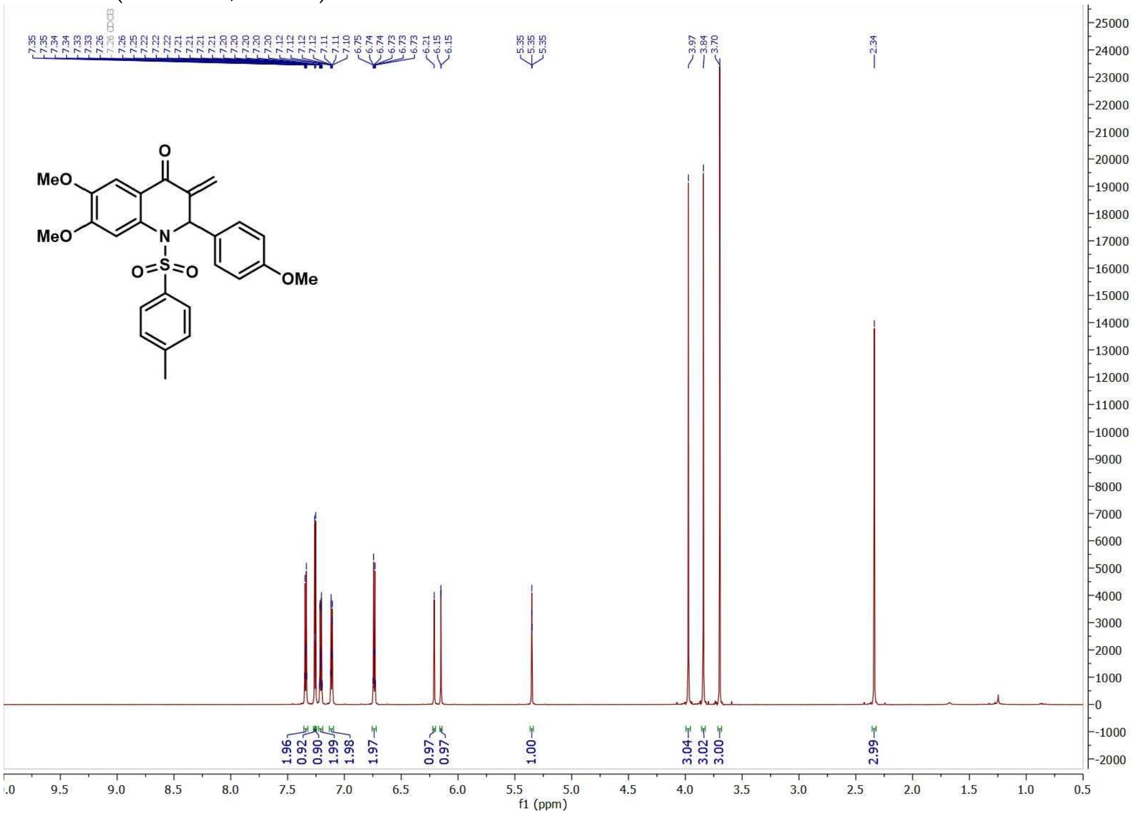

^{13}C NMR (176 MHz, CDCl$_3$)

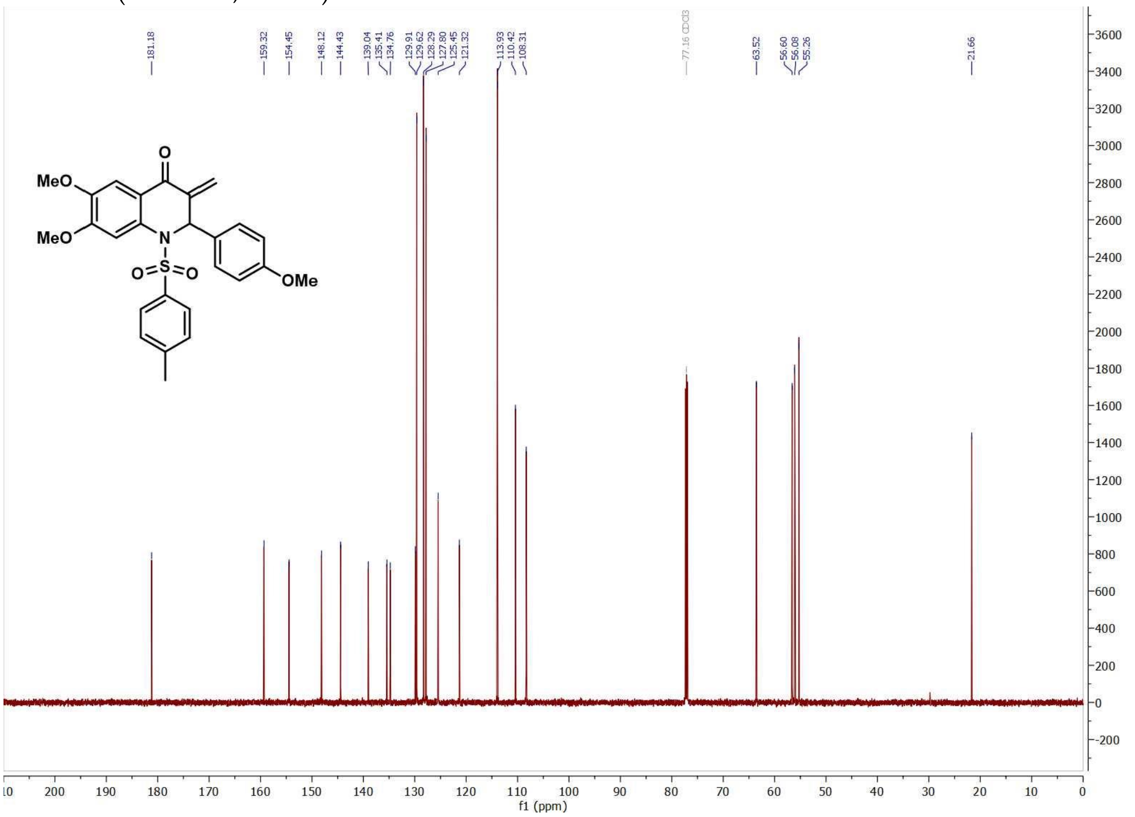

6-Chloro-2-ethyl-3-methylidene-1-tosyl-2,3-dihydroquinolin-4(1H)-one (**5m**)

^{1}H-NMR (700 MHz, CDCl$_3$)

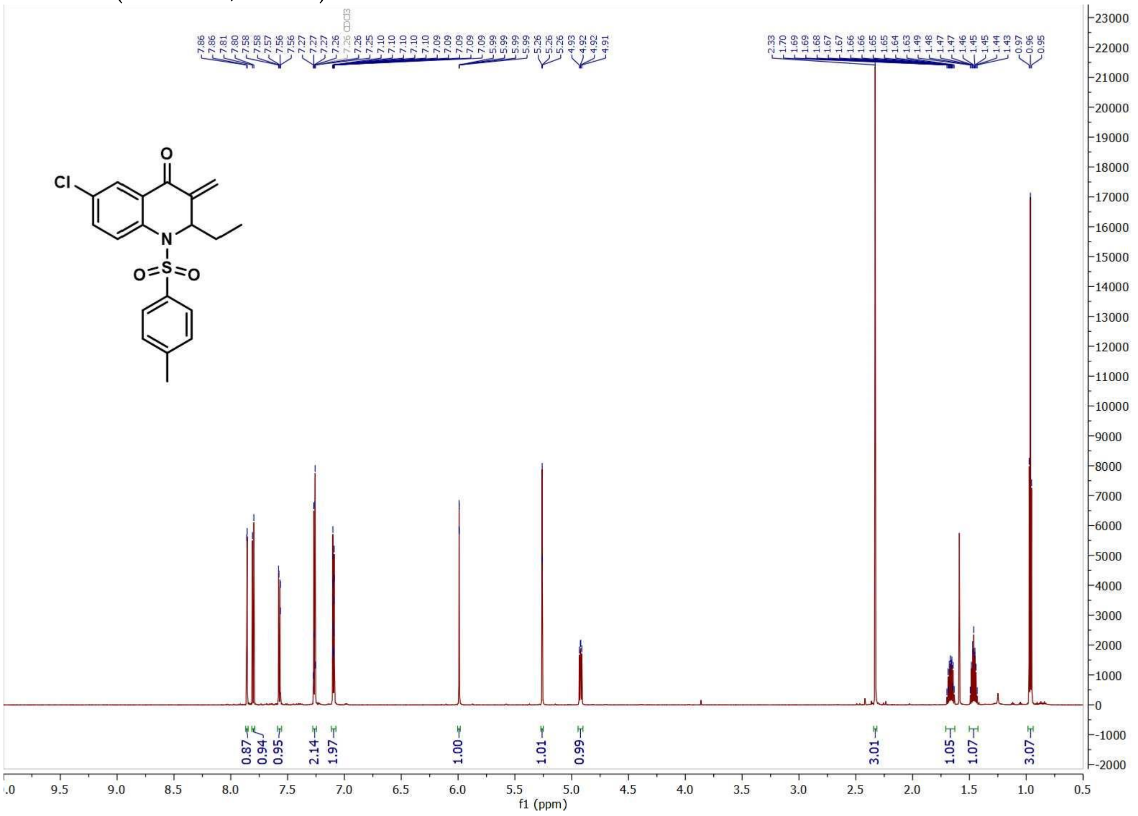

^{13}C NMR (176 MHz, CDCl$_3$)

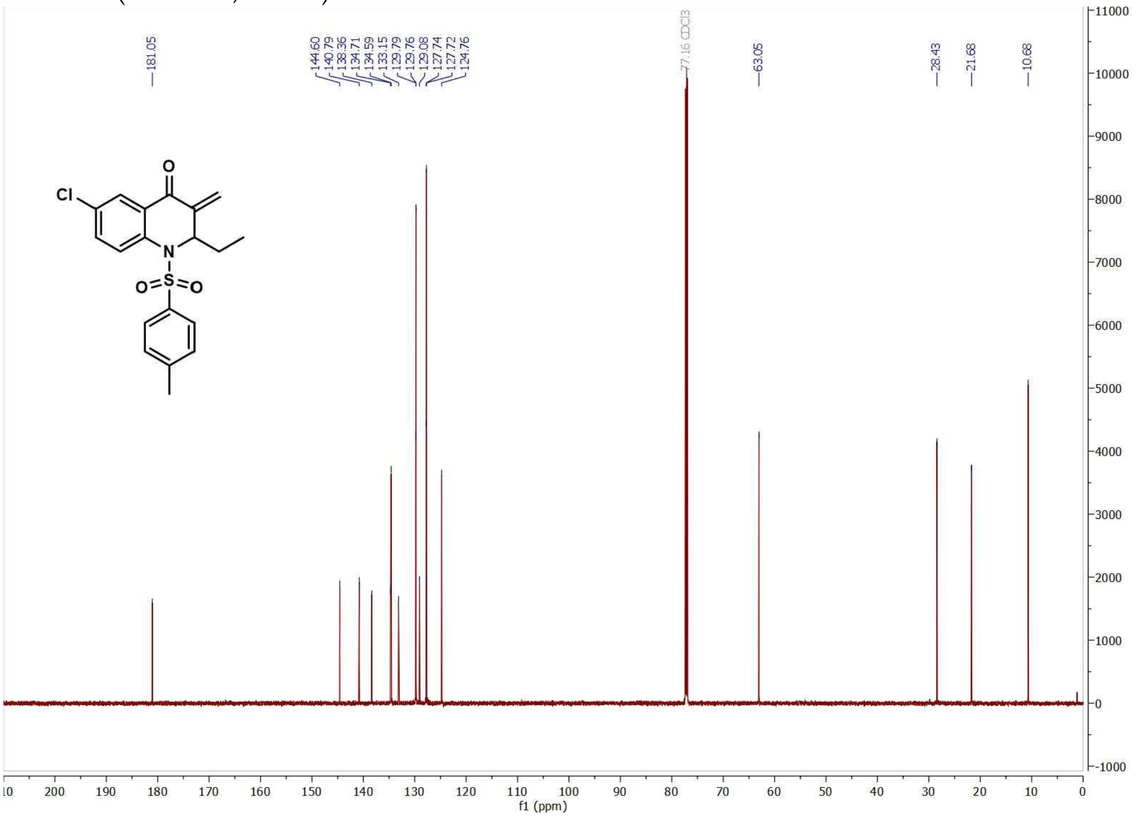

6-Chloro-3-methylidene-2-(propan-2-yl)-1-tosyl-2,3-dihydroquinolin-4(1H)-one (**5n**)

^{1}H-NMR (700 MHz, $CDCl_3$)

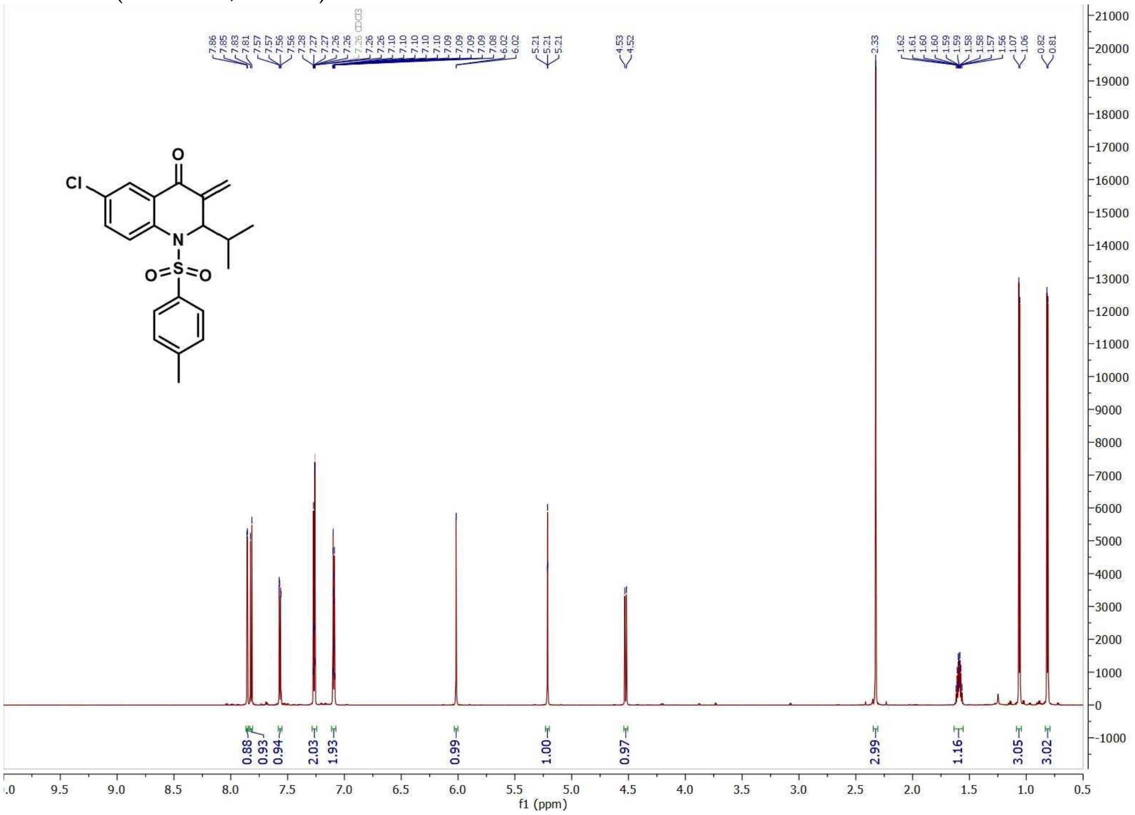

^{13}C NMR (176 MHz, $CDCl_3$)

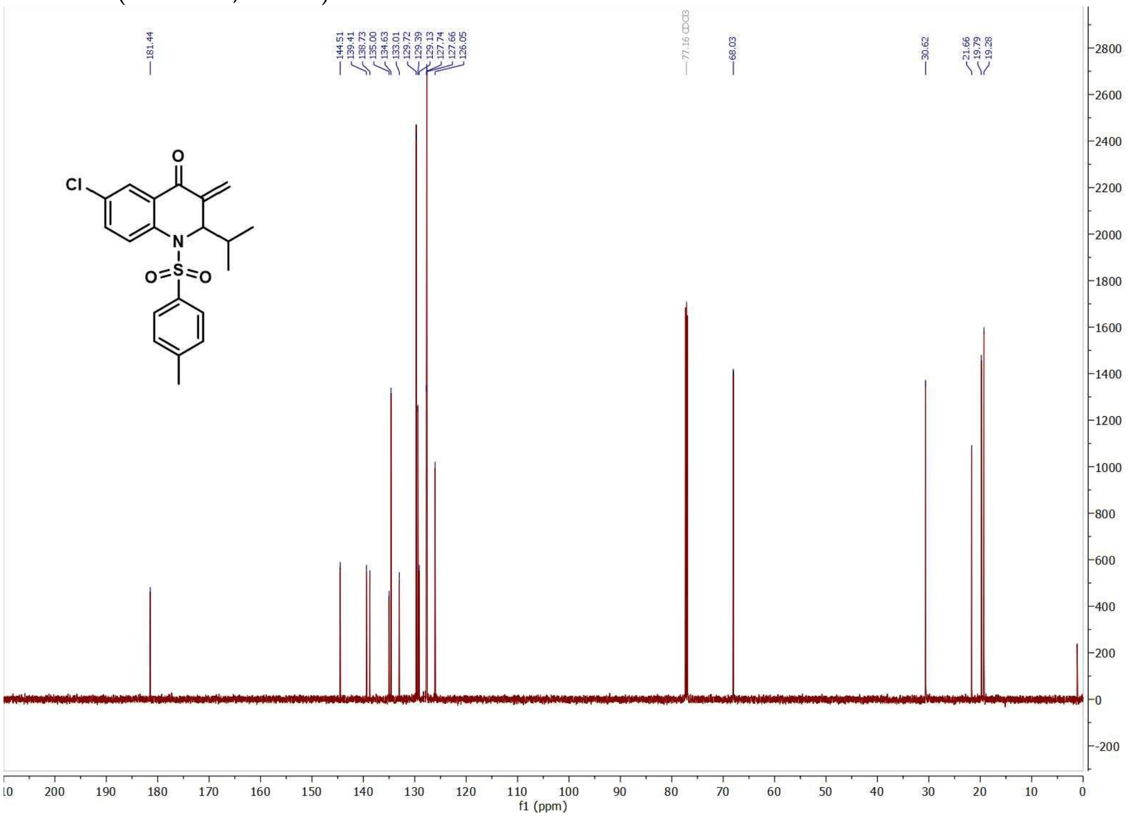

6-Chloro-3-methylidene-2-phenyl-1-tosyl-2,3-dihydroquinolin-4(1H)-one (**5o**)

^{1}H-NMR (700 MHz, $CDCl_3$)

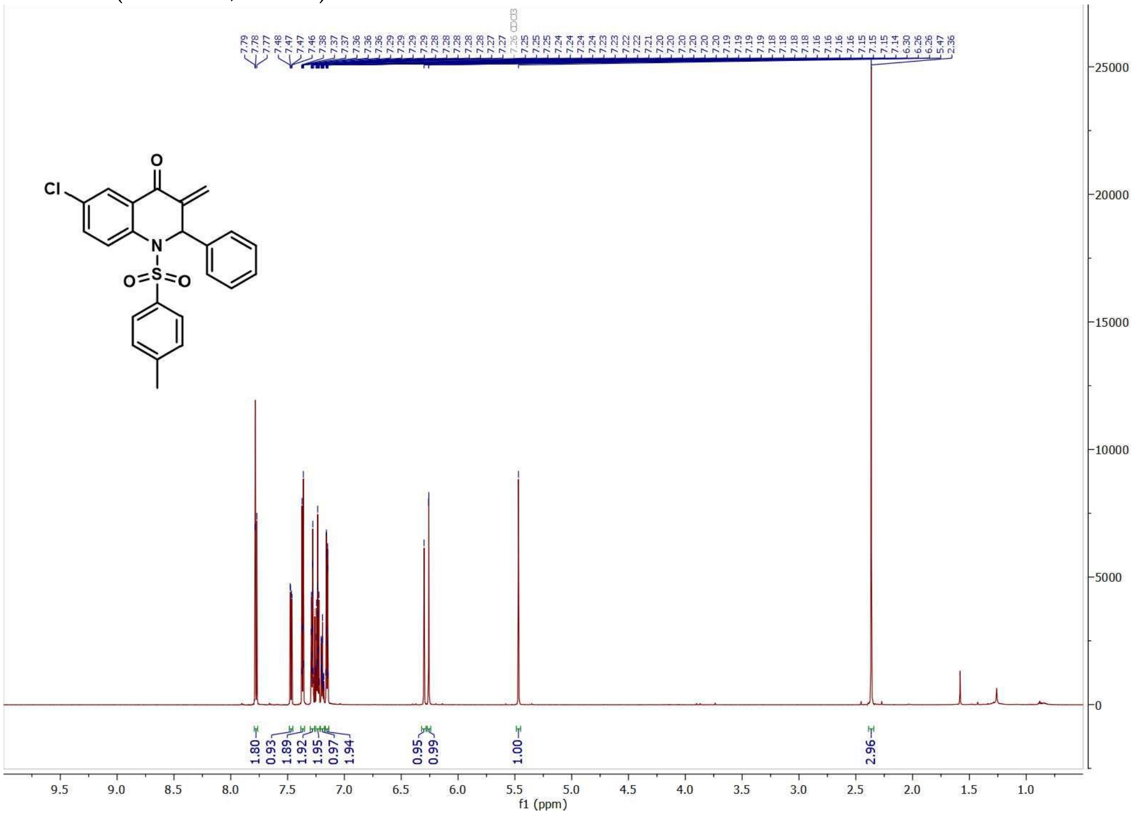

^{13}C NMR (176 MHz, $CDCl_3$)

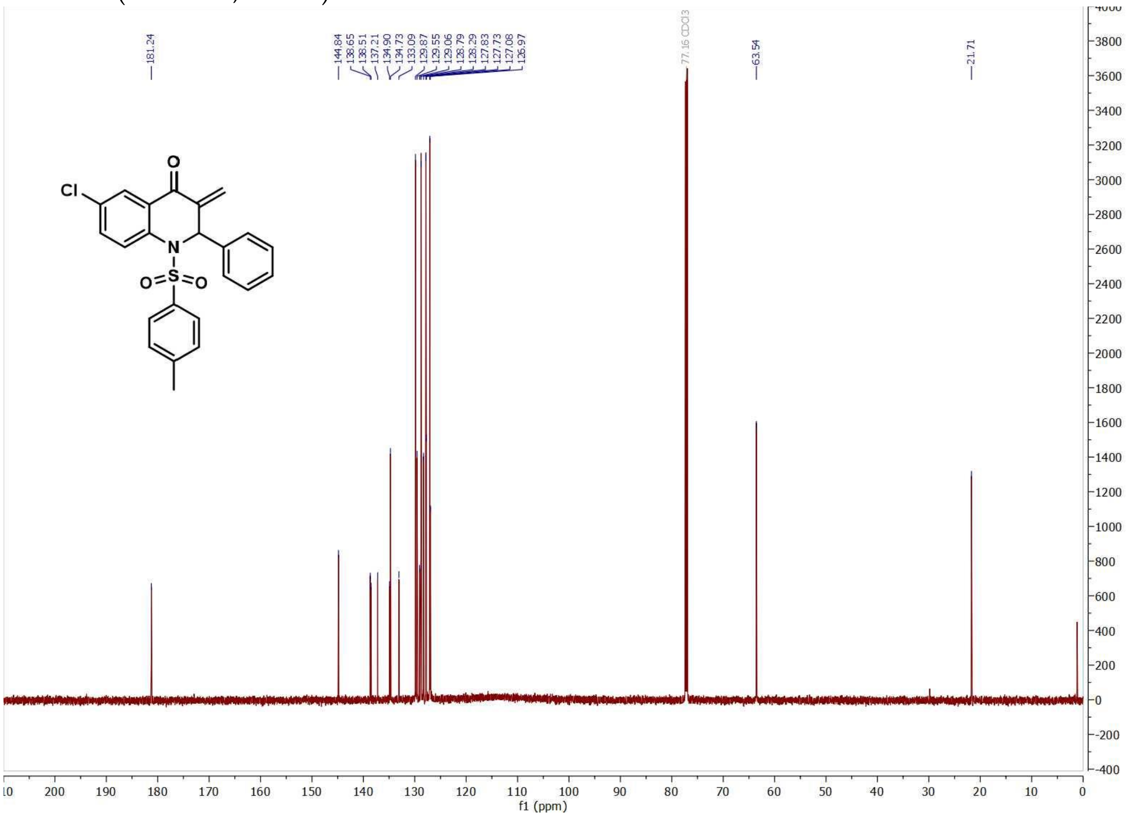

6-Chloro-2-(4-methoxyphenyl)-3-methylidene-1-tosyl-2,3-dihydroquinolin-4(1H)-one (**5p**)

^{1}H-NMR (700 MHz, $CDCl_3$)

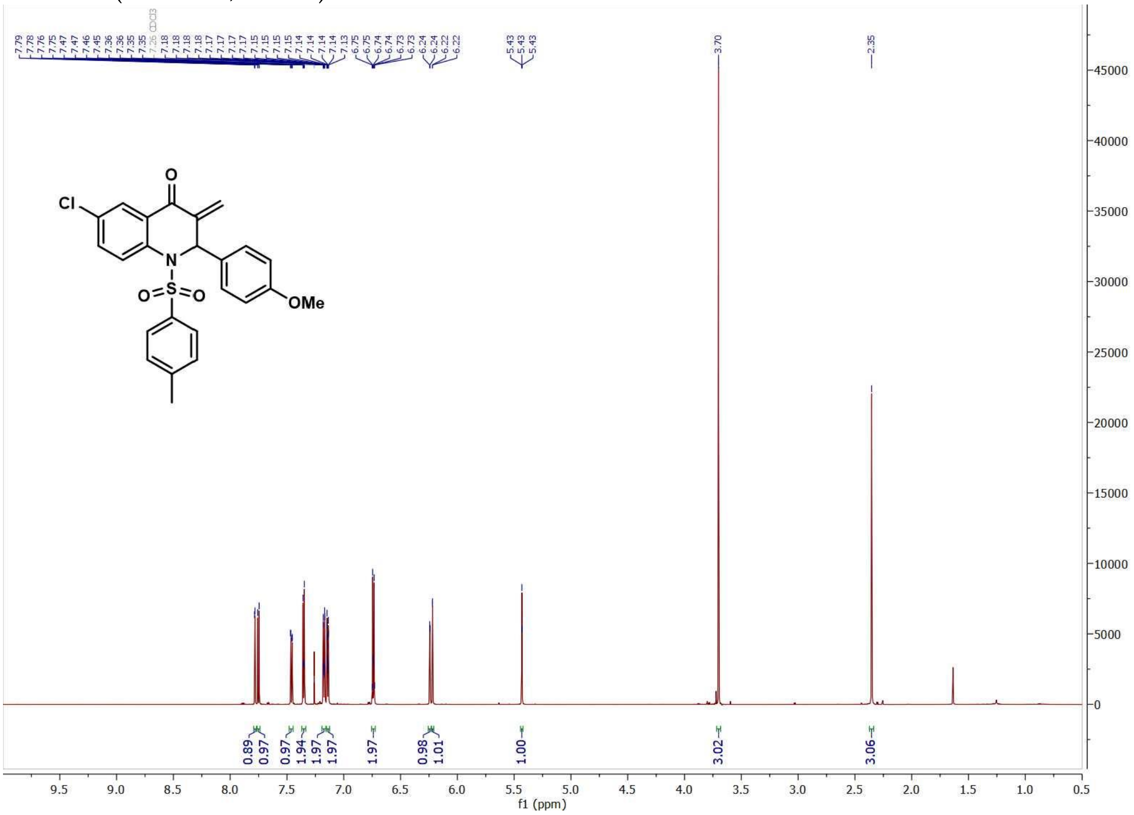

^{13}C NMR (176 MHz, $CDCl_3$)

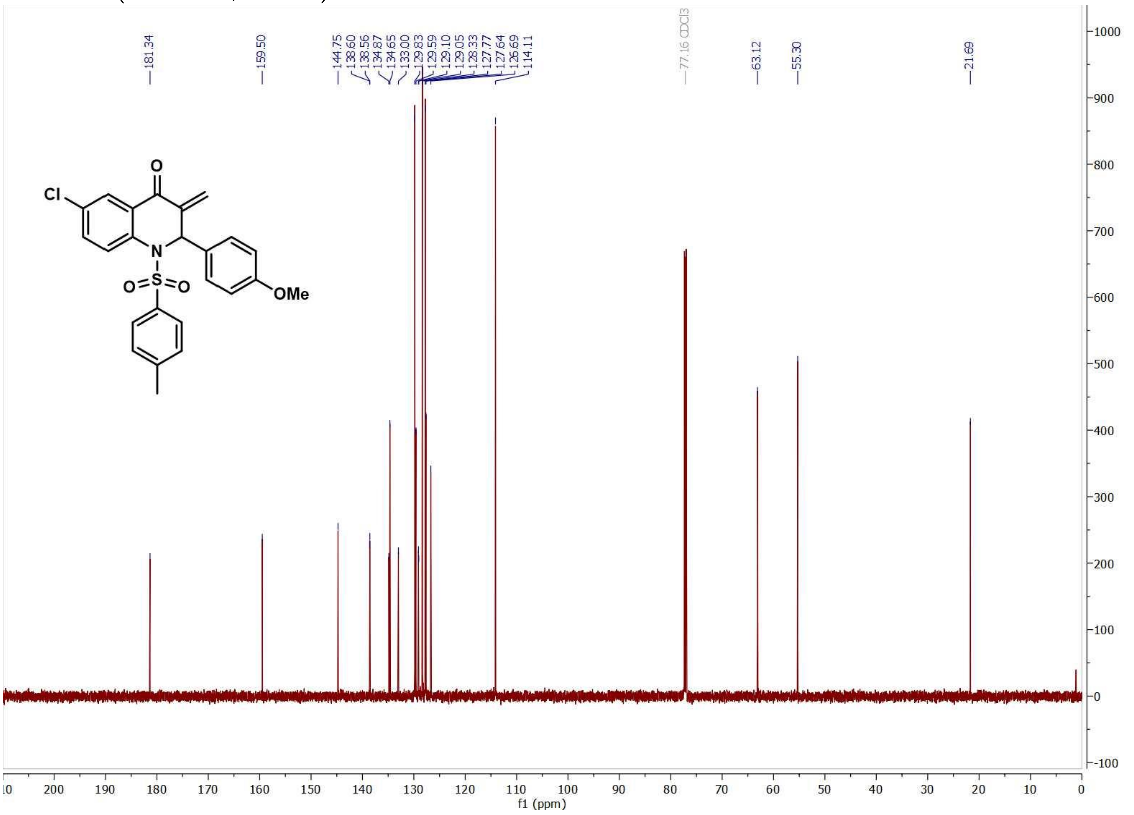

6-Bromo-2-ethyl-3-methylidene-1-tosyl-2,3-dihydroquinolin-4(1H)-one (**5q**)

^{1}H-NMR (700 MHz, $CDCl_3$)

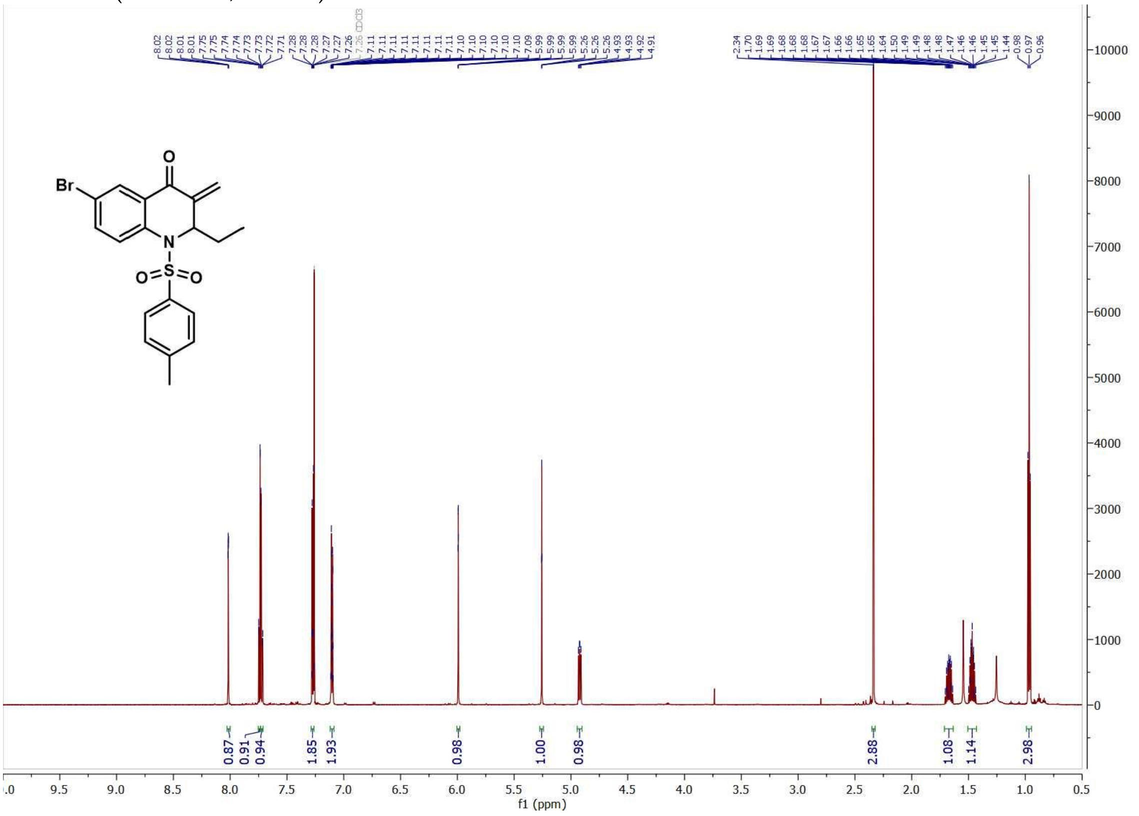

^{13}C NMR (176 MHz, $CDCl_3$)

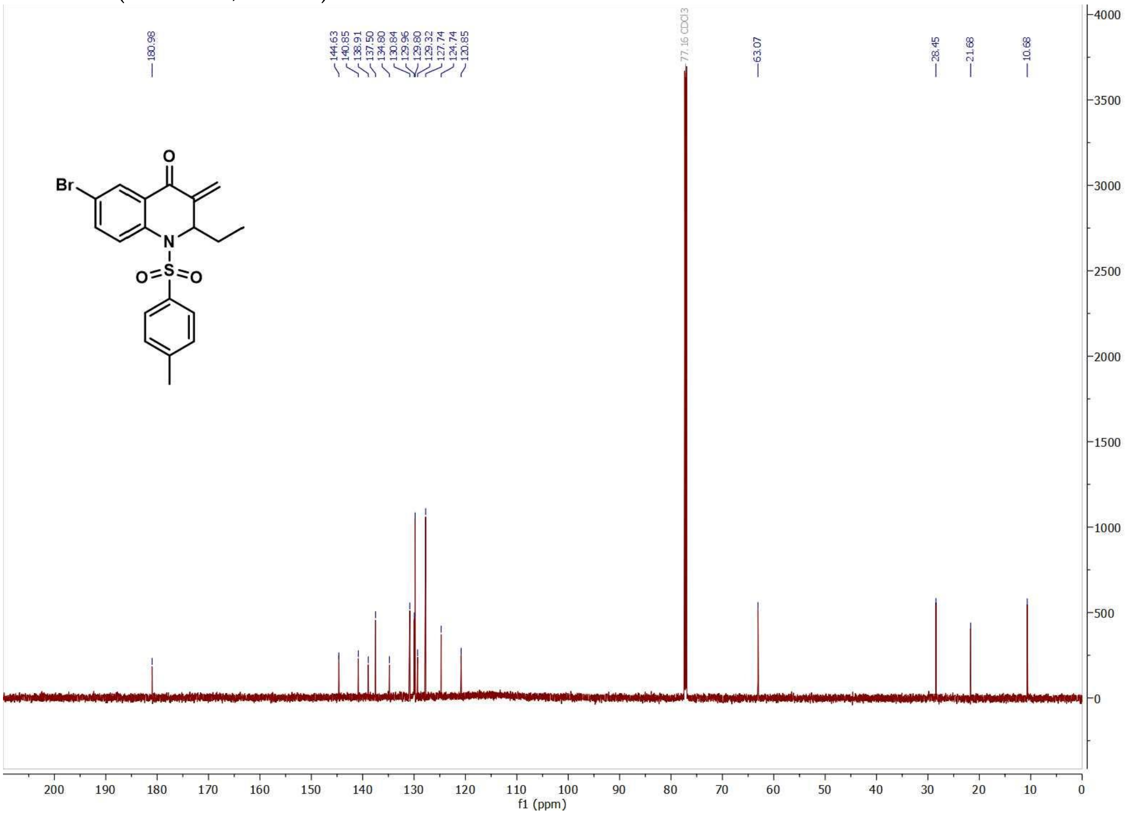

6-Bromo-3-methylidene-2-(propan-2-yl)-1-tosyl-2,3-dihydroquinolin-4(1H)-one (**5r**)

^{1}H-NMR (700 MHz, $CDCl_3$)

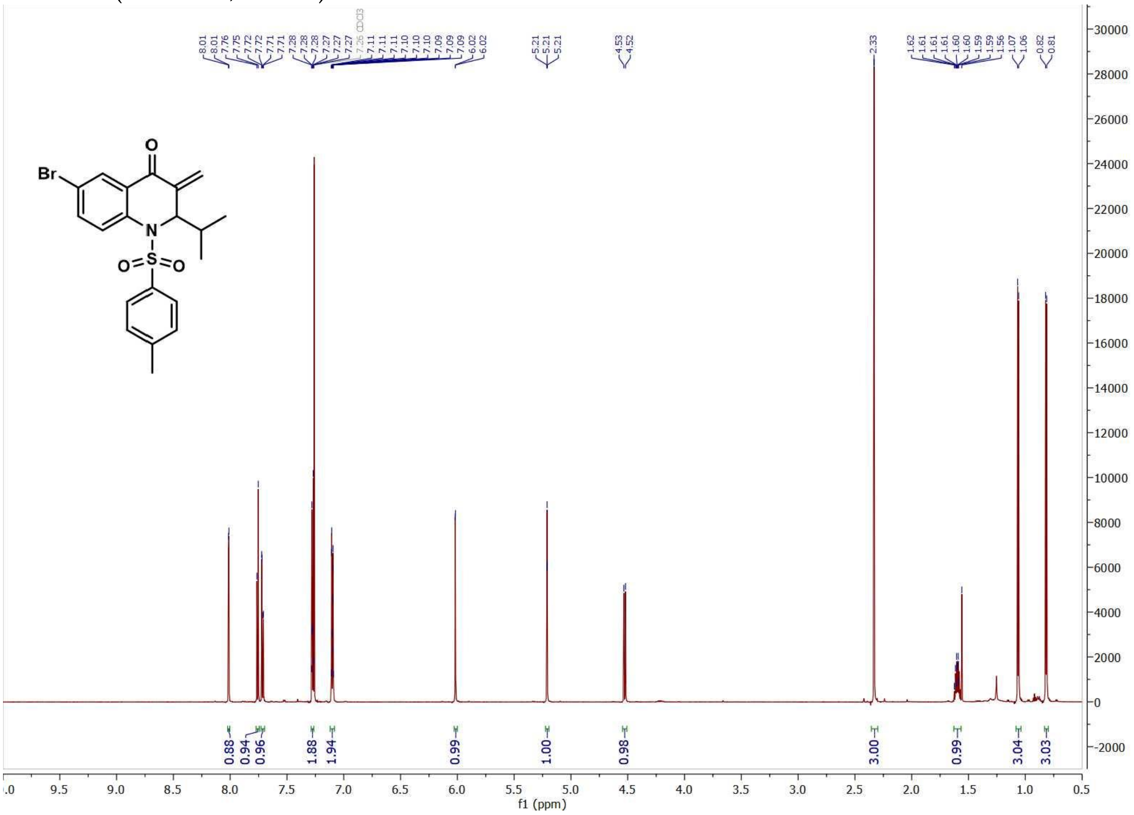

^{13}C NMR (176 MHz, $CDCl_3$)

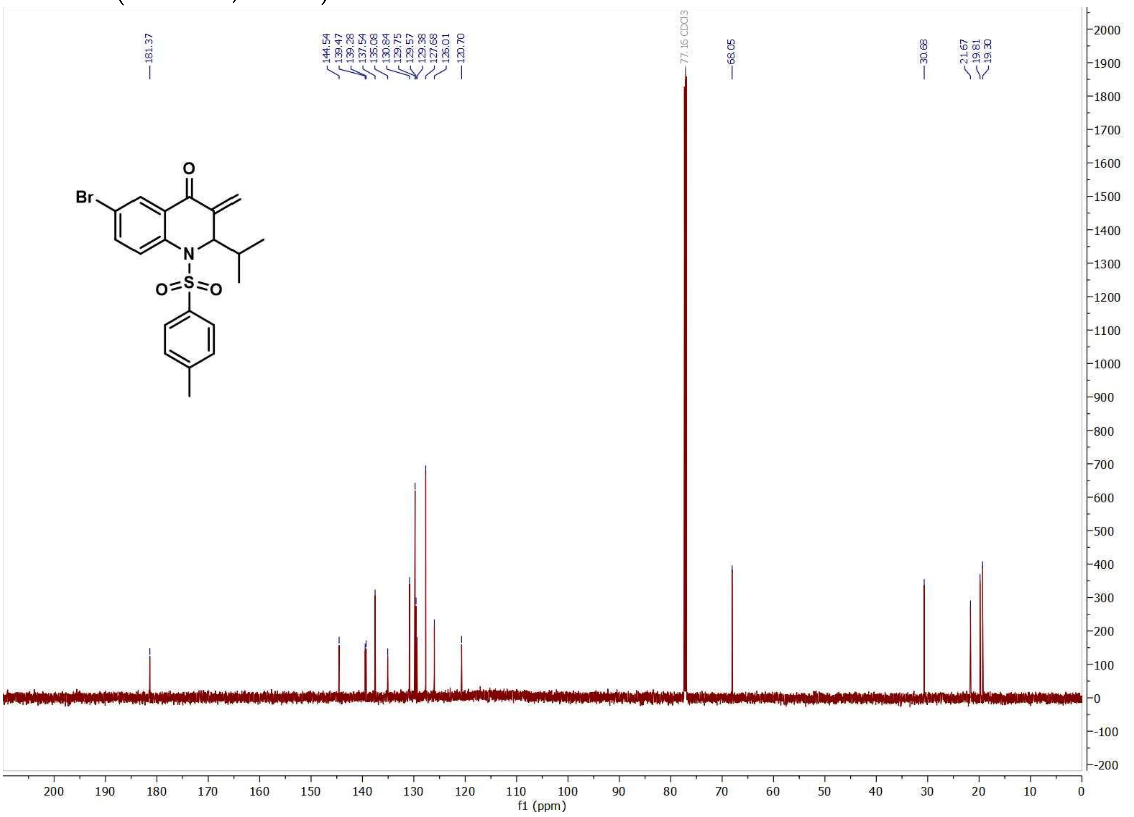

6-Bromo-3-methylidene-2-phenyl-1-tosyl-2,3-dihydroquinolin-4(1H)-one (**5s**)

^{1}H-NMR (700 MHz, $CDCl_3$)

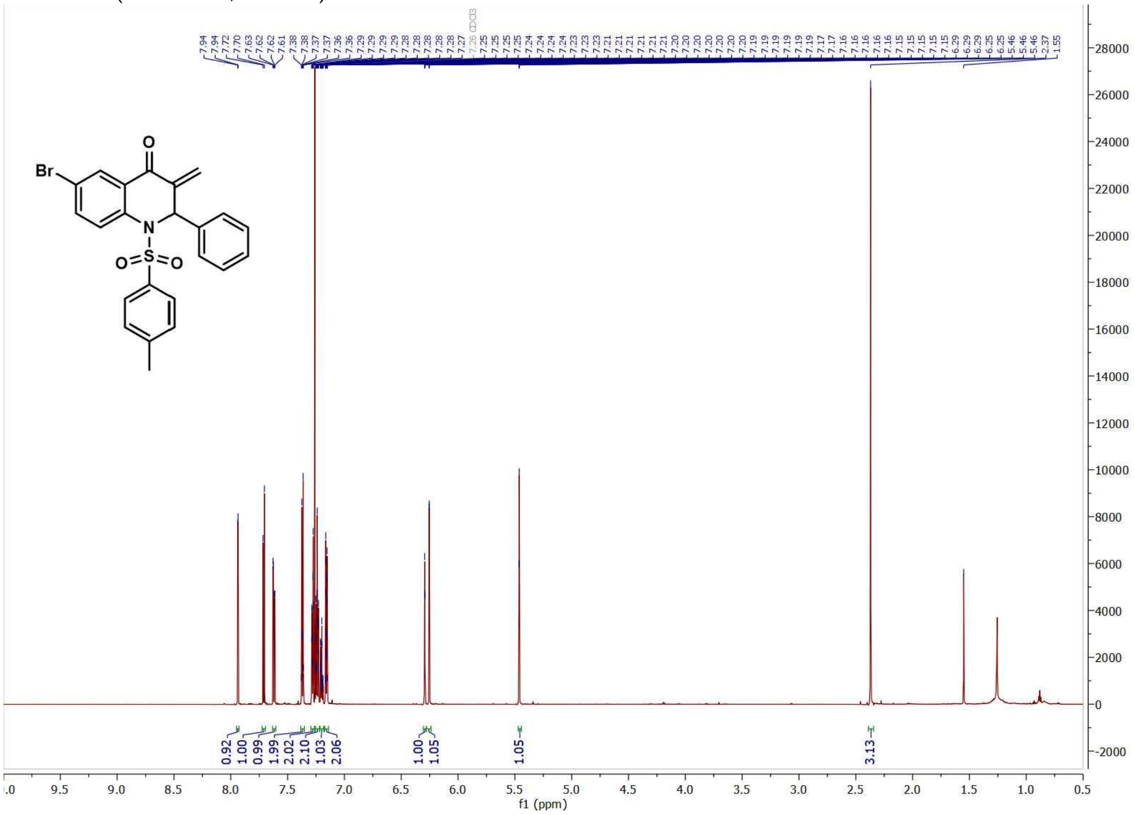

^{13}C NMR (176 MHz, $CDCl_3$)

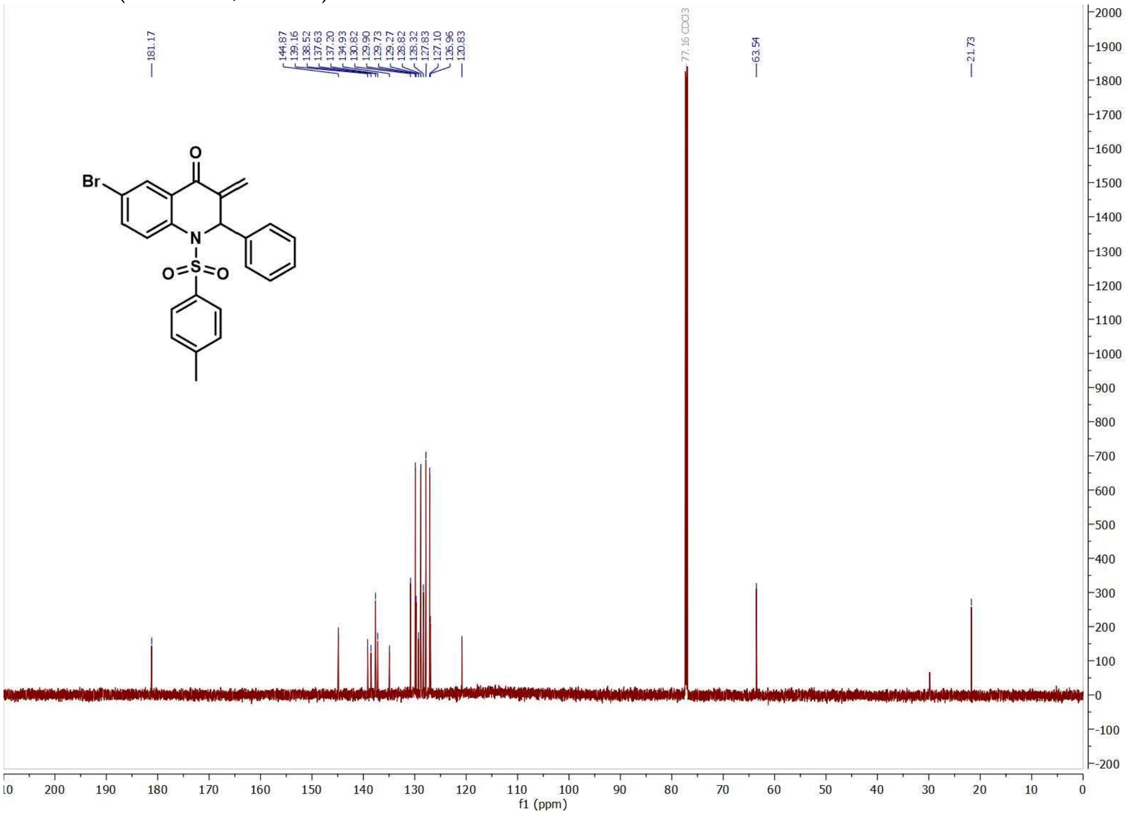

6-Bromo-2-(4-methoxyphenyl)-3-methylidene-1-tosyl-2,3-dihydroquinolin-4(1H)-one (**5t**)

^{1}H-NMR (700 MHz, $CDCl_3$)

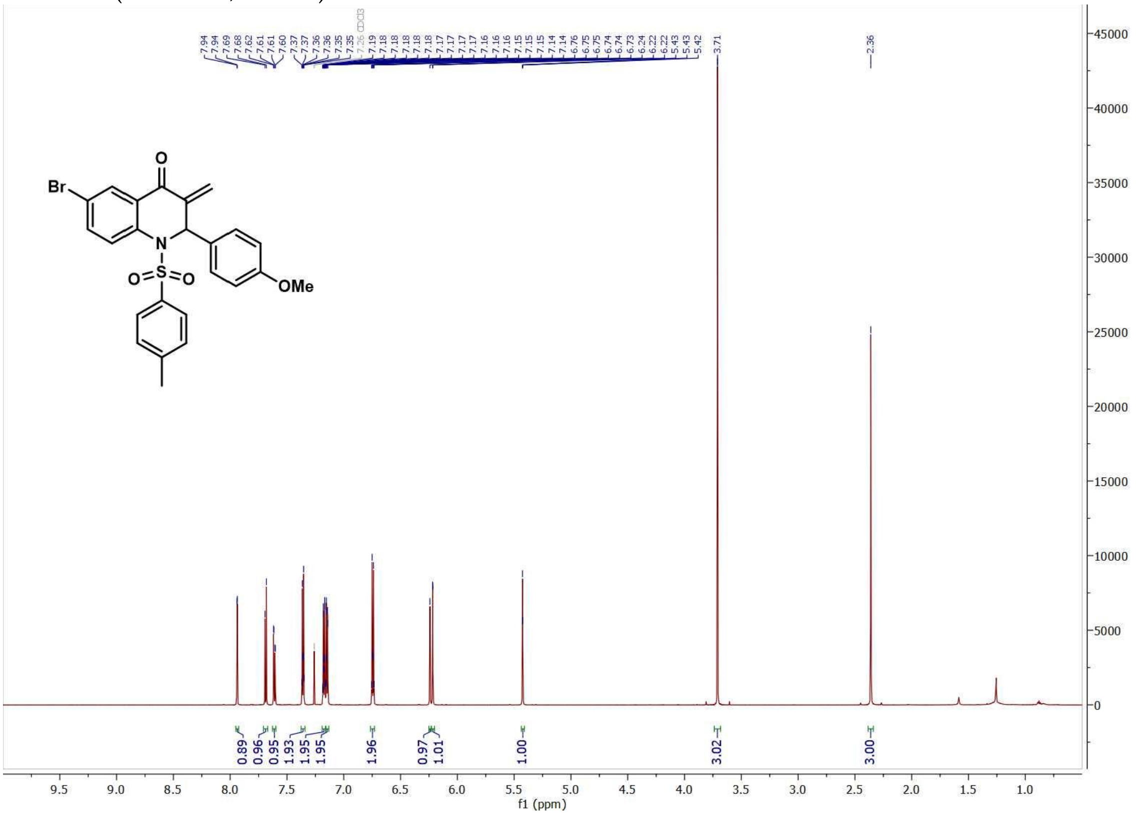

^{13}C NMR (176 MHz, $CDCl_3$)

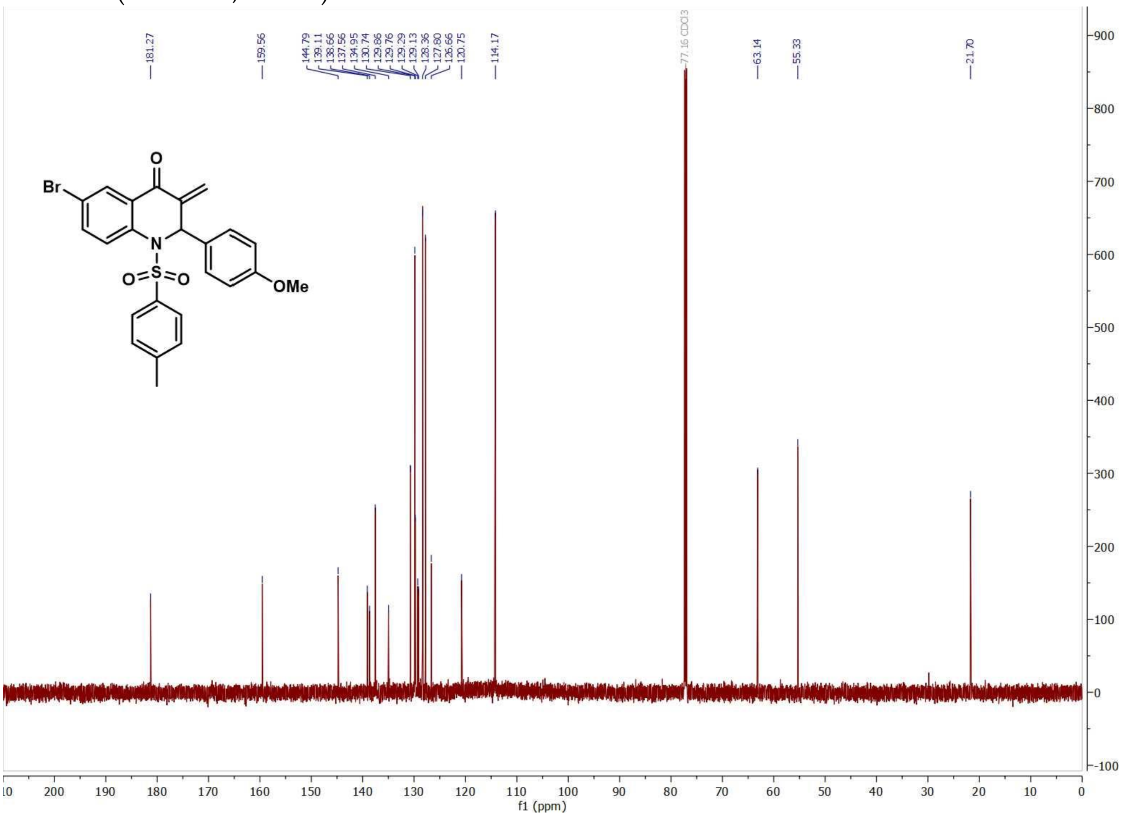

Supplement: Supplementary file 1 [file molecules-27-03597-s001.zip › molecules-1685920-supplementary.pdf]
